# Cholesterol at the Center of Alzheimer’s Disease: A Unifying Hypothesis on the Pathogenic Mechanism

**DOI:** 10.3390/molecules31142418

**Published:** 2026-07-09

**Authors:** Bao Ting Zhu

**Affiliations:** Shenzhen Key Laboratory of Steroid Drug Discovery and Development, School of Medicine, The Chinese University of Hong Kong, Shenzhen 518172, China; BTZhu@CUHK.edu.cn

**Keywords:** Alzheimer’s disease, pathogenic mechanism, cholesterol, neuronal cholesterol dysregulation, apolipoprotein E, amyloid *β*, tauopathy

## Abstract

It is hypothesized that in most cases of sporadic late-onset Alzheimer’s disease (LOAD), the abnormally elevated cholesterol level in brain neurons represents a critical causative factor that drives the pathogenic processes of LOAD. Specifically, it is hypothesized that the abnormally elevated neuronal cholesterol will disrupt mitochondrial structure and metabolic activity, resulting in ATP deficiency as well as reduced formation of neuroactive metabolic intermediates (such as mevalonate and geranylgeraniol) along the cholesterol synthesis pathway in brain neurons. In addition, the abnormally elevated neuronal cholesterol will cause direct neuronal damage as well as other pathogenic changes in the brain, including increased formation and deposition of amyloid *β* (A*β*) plaques. It is speculated that A*β* accumulation and plaque formation in most LOAD cases only represent characteristic secondary pathological changes and are usually not the main force driving the pathogenesis of LOAD. As discussed in detail in this paper, abnormally elevated neuronal cholesterol in conjunction with ATP deficiency and lack of neuroactive metabolic intermediates will not only cause learning and memory impairment, but will also induce tauopathy and reduce the formation of cholinergic vesicles. It is expected that these pathogenic changes are more readily seen initially in ischemia-sensitive neurons in hippocampus and posterior parietal cortex, which are then followed by neurodegenerative and atrophic changes in other brain regions along with progressive cognitive decline. As explained in this paper, ApoE4 is a major risk factor in LOAD because it has a drastically reduced ability than ApoE2 and ApoE3 to efflux excess cholesterol out of neurons. Overall, there is a large body of direct, indirect and circumstantial clinical and experimental evidence which jointly offers strong support for the cholesterol-centered hypothesis on the etiology and pathogenesis of LOAD. Considerable efforts are made to apply the proposed hypothesis to offer a better mechanistic explanation for many of the poorly understood experimental and/or clinical observations related to AD (mostly LOAD).

## 1. Introduction

Alzheimer’s disease (AD), first described by German physician Alois Alzheimer in the early part of the 20th century [1], is now recognized as the most prevalent irreversible neurodegenerative dementia among the elderly [2]. Presently, there are approximately 50 million people affected worldwide, and this figure is projected to triple by 2050 [2]. Clinically, AD can be categorized into the following two common forms: early-onset AD (EOAD) has an age of onset before 65 years, and late-onset AD (LOAD) has an age of onset ≥65 years [1]. Although these two forms of AD share similar core neuropathology, i.e., amyloid *β* (A*β*) plaques and tau neurofibrillary tangles (NFTs) [3,4,5,6], they differ sharply in genetics, clinical features, cognition, progression, and comorbidity [4,5]. Studies have revealed that LOAD is a memory-dominant sporadic AD subtype driven by polygenic risk and aging, with slow progression, frequent mixed brain pathologies and vascular comorbidities. In comparison, EOAD is a genetically-driven, non-amnestic cortical-predominant AD subtype usually with rapid decline, pure AD neuropathology, atypical clinical presentations, and frequent familial monogenic inheritance. Major characteristics and differences in LOAD vs. EOAD are summarized in Table 1 below.

The causes of AD are multifactorial, arising from a combination of genetic, aging, pathological, lifestyle and environmental factors [4,5]. Genetically, EOAD is closely linked to mutations in *APP*, *PSEN1*/*PSEN2* [7,8,9], while the *APOE ε4* allele is the strongest genetic risk for LOAD [5,10]. Advanced age is the greatest non-modifiable risk factor in LOAD, driving brain atrophy and neuronal vulnerability. Environmental factors, such as long-term air pollution, head trauma, chronic exposure to heavy metals (aluminum, lead, and mercury), and certain pesticides/herbicides, are among the important risk factors for AD [11,12,13].

In the past two decades, epidemiological studies have led to the suggestion that hypercholesterolemia is an important modifiable risk factor of LOAD [14,15,16,17,18,19]. Similarly, alterations in brain cholesterol metabolism were also found to be associated with increased LOAD risk [19,20,21,22]. Animal studies have more clearly shown that hypercholesterolemia is associated with increased production, aggregation, and cerebral deposition of A*β* peptides, along with learning and memory impairment [23,24]. Further in vitro studies found that elevated cholesterol in neuronal plasma membrane can activate *γ*-secretase-catalyzed A*β* formation [25,26,27,28,29,30]. Similarly, high levels of free cholesterol in cultured neurons are associated with increased NFTs in these neurons [31].

The above observations indicate that abnormally elevated brain cholesterol constitutes an important risk factor in AD, but the precise role and particularly the mechanism of action of cholesterol in AD pathogenesis are still not clearly understood. This dilemma is partly complicated by the fact that cholesterol is an important endogenous compound that is closely involved in many normal brain functions. In this paper, a unifying hypothesis is proposed, which postulates that in most cases of AD (LOAD in particular), abnormally elevated cholesterol in brain neurons constitutes a crucial causative factor, which may drive many key pathogenic processes of AD, including learning and memory impairment, cholinergic deficiency, A*β* plaque formation, tauopathy, and ultimately, neuronal death. This hypothesis was partly prompted by our recent observation that free unmetabolized cholesterol, when present at even very low concentrations (<1 µM), can selectively disrupt mitochondrial structure and function, resulting in reduced ATP synthesis in cultured hippocampal neurons [32]. In this paper, a number of detailed hypotheses are proposed to provide a better mechanistic explanation that abnormally elevated neuronal cholesterol contributes to certain aspects of the AD pathogenic process. An in-depth analysis of the proposed hypotheses, along with a discussion of the supporting experimental and clinical evidence, is provided below.

## 2. Methods: Literature Source and Selection

In this conceptual hypothesis paper, all literature consulted (both cited and uncited) was retrieved from publicly accessible academic databases, with PubMed and Web of Science serving as the primary sources. A large set of customized search terms and term combinations was adopted to screen and identify the peer-reviewed literature relevant to the research topic under discussion. The initial literature search often was primarily restricted to publications published within the past 5–10 years, with the time frame flexibly adjusted according to the total volume of available relevant studies on a given topic. Furthermore, backward snowballing of references from selected recent publications was performed to supplement key earlier studies associated with the research topic of interest.

As this is a hypothesis-driven review article, the core practical criterion for literature selection is the identification and inclusion of credible scientific studies that provide direct, indirect, or circumstantial evidence to support or refute the proposed hypothesis. When the relevant literature was identified, it was subjected to further careful reading and subsequent formal citation in this paper. For a given topic with many published studies, only the representative literature was selected for citation, covering both original foundational observations and recent high-quality studies with more robust novel evidence. Accordingly, the citations presented in this paper are not intended to constitute a comprehensive inventory of the existing literature; instead, their inclusion is aimed at enabling a balanced discussion of the available evidence both for and against the proposed hypothesis. Here, I would like to apologize for the fact that many equally valuable studies were not included for citation in the reference list due to journal space consideration (quite a lot of cited references were later removed during revisions).

**Figure 1:**
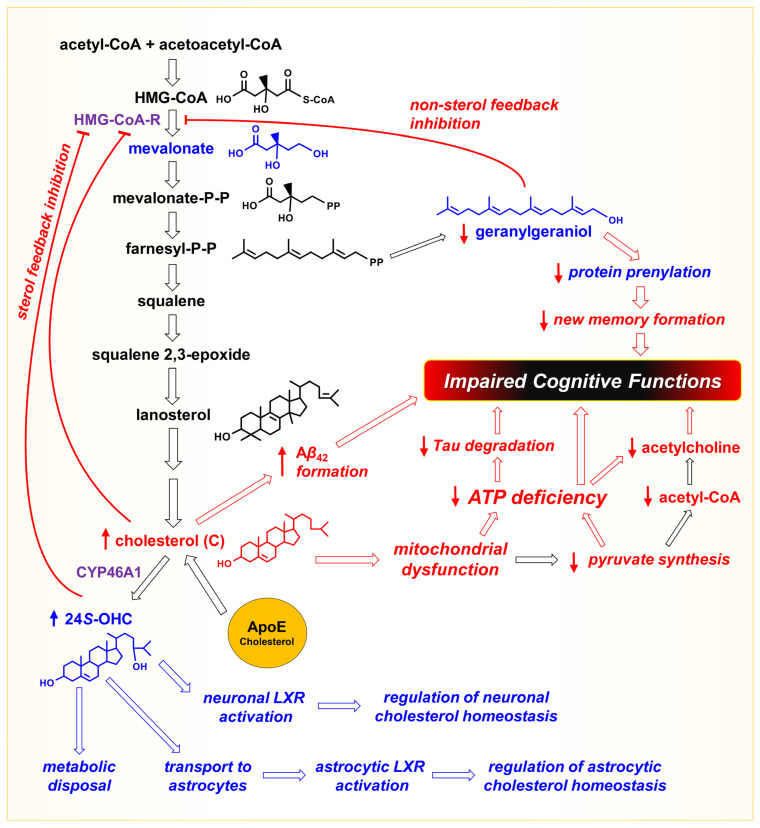
A hypothesis on the etiological role of neuronal cholesterol dysregulation in LOAD. As depicted in the upper left (in black color), cholesterol is synthesized using acetyl-CoA as the initial substrate (all the enzymes involved in cholesterol synthesis and metabolism are separately listed in Figure 2). It is hypothesized that elevated free cholesterol (i.e., unmetabolized cholesterol) can disrupt mitochondrial structure and function, resulting in ATP deficiency in brain neurons as well as other cells. In addition, high levels of neuronal cholesterol will also suppress the cholesterol synthesis pathway through feedback inhibition of HMG-CoA reductase (HMG-CoA-R or HMGR), which then decreases the formation of key neuroactive metabolic intermediates (such as mevalonate and geranylgeraniol) in neurons. These neuroactive metabolic intermediates play a vital role in protein prenylation and synaptogenesis. Collectively, these biochemical changes resulting from elevated neuronal cholesterol will gradually lead to learning and memory impairment (explained in detail later). Additionally, abnormally elevated neuronal cholesterol will drive the formation and aggregation of A*β*_42_ and A*β*_40_ (i.e., amyloid plaque formation) in the brain. Mitochondrial dysfunction and ATP deficiency will also contribute to reduced degradation of the hyperphosphorylated tau proteins as well as reduced synthesis and release of the cholinergic vesicles. Please refer to the manuscript for a detailed explanation of each of the above hypothetical elements.

Lastly, I would like to note that when a piece of “conflicting observation or evidence” is found in the literature, I would usually spend a lot more time carefully assessing the original study (or studies) reporting the observation, and would also try to find more relevant information surrounding that particular study. Special efforts would be made to find a scientifically sound alternative explanation for the seemingly conflicting observation. If a credible, scientifically sound alternative mechanistic explanation could be successfully found for it, then it would usually help me gain a deeper and better understanding of the proposed hypothesis. Under such circumstances, the seemingly “conflicting observation or evidence” is sometimes being turned into a unique piece of supporting evidence for the proposed hypothesis. On the other hand, if no satisfactory explanation for the conflicting observation could be found, then the original hypothesis or explanation was usually shelved for the time being or permanently abandoned. Here, it is worth noting that during the preparation of this cholesterol-centered hypothesis over the past 10 years or so, some of the experimental or clinical observations were initially found to cast serious doubts in my mind on the proposed hypothesis. Later, through in-depth logical thinking and extensive literature searching and reading, I was able to find some scientifically sound, better alternative explanations for these seemingly “conflicting observations” on the basis of the proposed hypothesis. This is also one of the reasons that this hypothesis paper is much longer than usual, as I have tried to offer a better mechanistic explanation for many of the poorly-understood or even puzzling experimental or clinical observations contained in the literature on the basis of the proposed cholesterol-centered hypothesis on AD etiology and pathogenesis.

## 3. Hypothesis

Cholesterol is involved in many important functions in the central nervous system (CNS), such as learning and memory formation [33,34,35,36], synaptogenesis [37], axonal growth and neuronal regeneration [38]. During these processes, brain neurons require an increased supply of cholesterol. It is generally thought that the neighboring astrocytes are the main source of cholesterol for brain neurons [39], although neurons also have the ability to synthesize and supply small amounts of cholesterol for their own needs under certain conditions. The cholesterol molecules synthesized in brain astrocytes are carried mostly in lipidated ApoE particles, although other apolipoproteins such as ApoA-I, ApoA-II, ApoA-IV, ApoJ, ApoD and ApoH [40,41] are also involved in carrying, to varying degrees, cholesterol in the brain. These lipoproteins are delivered to neurons through receptor-mediated endocytosis. A number of receptors and proteins are involved in mediating the endocytosis of lipidated ApoE particles by neurons (discussed later).

As cholesterol is required for maintaining the normal structure and function of brain neurons and their synapses, it is understood that if neuronal cholesterol supply becomes severely inadequate, some of its normal functions will be compromised. In line with this suggestion, an earlier study reported that when cholesterol content in hippocampal region is reduced by statin treatment, synaptic density is reduced and synaptic vesicular release is impaired [42]. Similarly, selective loss of astrocytic cholesterol synthesis, which is the main source of cholesterol in the brain [39], alters brain development and synaptic functions, including reduced synaptic vesicle numbers and defective synaptic plasticity [43,44].

Here, it should also be clearly pointed out that when neuronal cholesterol content is abnormally elevated, it can become strongly cytotoxic [32,45,46,47,48]. It is hypothesized that abnormally elevated cholesterol content in brain neurons constitutes a major causative factor, which drives the pathogenic processes in most cases of LOAD (schematically depicted in Figure 1). Specifically, based on the observations made recently [32] and earlier [45,46,47,48], it is hypothesized that elevated free cholesterol (i.e., unmetabolized cholesterol) can disrupt mitochondrial structure and function, resulting in ATP deficiency in neurons as well as other cells. In addition, high levels of neuronal cholesterol will suppress the cholesterol synthesis pathway through feedback inhibition of the HMG-CoA reductase (HMG-CoA-R or HMGR) [49,50], which then reduces the formation of key neuroactive metabolic intermediates (such as mevalonate and geranylgeraniol) in neuronal cells [50,51]. These neuroactive metabolic intermediates play a vital role in protein prenylation and synaptogenesis [51]. Jointly, these biochemical changes due to elevated neuronal cholesterol gradually will lead to learning and memory impairment and tauopathy (explained in later sections). Additionally, abnormally elevated neuronal cholesterol is known to drive the formation and aggregation of A*β*_42_ and A*β*_40_ (i.e., amyloid plaque formation) in the brain (reviewed in [25]). As discussed in later sections, A*β* accumulation and amyloid plaque formation in most LOAD cases only represent characteristic secondary pathological changes and are not the dominant force driving the pathogenic process of LOAD.

As briefly outlined below, the above cholesterol-centered hypothesis on the pathogenic mechanism of LOAD has the following major hypothetical elements (a detailed discussion of the supporting experimental evidence for each hypothetical element is provided separately in Section 4, Section 5, Section 6, Section 7, Section 8, Section 9 and Section 10):

***i.*** It is hypothesized that in brain neurons, chronically elevated cholesterol (in particular mitochondrial cholesterol) will cause disruption of mitochondrial structure and metabolic function, resulting in reduced ATP synthesis. This hypothesis is proposed on the basis of recent experimental findings [32].

It is known that an adequate supply of cellular ATP is important for maintaining cognitive function and new memory formation in the brain. It is hypothesized that cholesterol-induced decrease in mitochondrial ATP synthesis will create an energy deficiency in neurons, particularly in certain regions of the brain where neurons have a higher demand for oxygen and ATP supply to maintain their normal physiological functions. Inhibition of neuronal mitochondrial metabolic activity by cholesterol is an important early event that subsequently triggers a series of pathogenic changes (discussed in detail later), culminating in learning and memory impairment. Based on this understanding, it is speculated that most LOAD cases begin in brain regions that have a particularly high demand for oxygen and energy supply (ATP synthesis), and then gradually spread to other brain regions with a relatively lower demand for energy supply, and eventually to most regions of the brain.

***ii.*** The normal learning and memory process also requires the supply of certain neuroactive metabolic intermediates (such as mevalonate and geranylgeraniol) [35] for prenylation of proteins [51]. Offering support for the important role of these neuroactive metabolic intermediates in learning and memory, an earlier study demonstrated that impairments in learning and memory functions in an animal model could be restored by supplying geranylgeraniol, but not cholesterol [50].

Notably, both mevalonate and geranylgeraniol are metabolic intermediates formed in brain neurons as part of the cholesterol synthesis pathway [49,52,53] (see Figure 1). Elevated cholesterol levels in neurons will inhibit HMGR and thus suppress the cholesterol synthesis pathway. As a result, it will markedly reduce the levels of these neuroactive metabolic intermediates in brain neurons. Understandably, it is crucial that the upper half of the cholesterol synthesis pathway (depicted in Figure 1) in brain neurons needs to remain active for normal learning and memory function as it will provide key neuroactive metabolic intermediates required for the formation of new synaptic connections. Here, it should be noted that activation of the entire cholesterol synthesis pathway will lead to production of cholesterol, which may be used by neurons to fulfill certain physiological functions when it is so needed; however, when cholesterol is actually not needed by neurons or is already adequately supplied by neighboring astrocytes, the upper half of the cholesterol synthesis pathway in brain neurons will still need to be kept active, which is not for the purpose of synthesizing more cholesterol, but for the purpose of synthesizing neuroactive metabolic intermediates. To achieve this unique function without producing excess cholesterol in neurons, the upper half of the cholesterol synthesis pathway can be, in fact, effectively regulated by geranylgeraniol, which serves as a non-sterol feedback inhibitor of HMGR [49,52] (see Figure 1). In this way, when neurons have already synthesized sufficient amount of mevalonate and geranylgeraniol for protein prenylation, it can effectively shut down HMGR when further synthesis of cholesterol is not needed.

Understandably, when cholesterol level in a neuron is abnormally elevated (for whatever reason), it will always impose an inhibition of its HMGR, a rate-limiting enzyme in the cholesterol synthesis pathway. This inhibition will not only suppress the synthesis of cholesterol but will also suppress the production of mevalonate and geranylgeraniol. Additionally, elevated neuronal cholesterol will also disrupt mitochondrial structure and metabolic function and inhibit ATP synthesis (already described above). These pathogenic effects caused by elevated neuronal cholesterol will jointly hamper the normal process of learning and memory formation.

***iii.*** It is known that when neuronal cholesterol is abnormally elevated, it will lead to increased formation of A*β* plaques (mechanistic explanation is discussed later). In addition, elevated extracellular A*β*_42_ and A*β*_40_ levels can disrupt mitochondrial function, which contributes to reduced mitochondrial ATP synthesis [54,55].

***iv.*** It is well known that the intracellular protein tau is involved in AD pathogenesis by forming intracellular NFTs (discussed in Section 7). Tau protein degradation in neurons is mediated by the ubiquitin system in an ATP-dependent manner (discussed in Section 7). Elevated neuronal cholesterol will lead to reduced ATP synthesis, and it is, therefore, hypothesized that severe cellular ATP deficiency will not only disrupt the process of neurotransmission, but will also slow down proteasome-mediated degradation of tau proteins, which eventually results in the accumulation of hyperphosphorylated tau proteins in neurons, along with the formation of characteristic NFTs.

***v***. It is known that the cholinergic neurotransmitter vesicles contain high levels of ATP, which is a key component of the cholinergic vesicles [56,57]. It is hypothesized that severe deficiency of cholinergic vesicles seen in AD patients is, in part, due to disruption of mitochondrial metabolic activity by cholesterol, which reduces ATP synthesis, and the reduced neuronal ATP level is an important factor for the reduced formation of cholinergic vesicles. In addition, it is hypothesized that disruption of mitochondrial metabolic activity by cholesterol also reduces the synthesis and cross-mitochondrial transport of acetyl-CoA, a precursor for the synthesis of acetylcholine. It is speculated that these two factors jointly contribute to the reduced synthesis of acetylcholine and the reduced formation and release of cholinergic vesicles in many LOAD cases.

**Figure 2:**
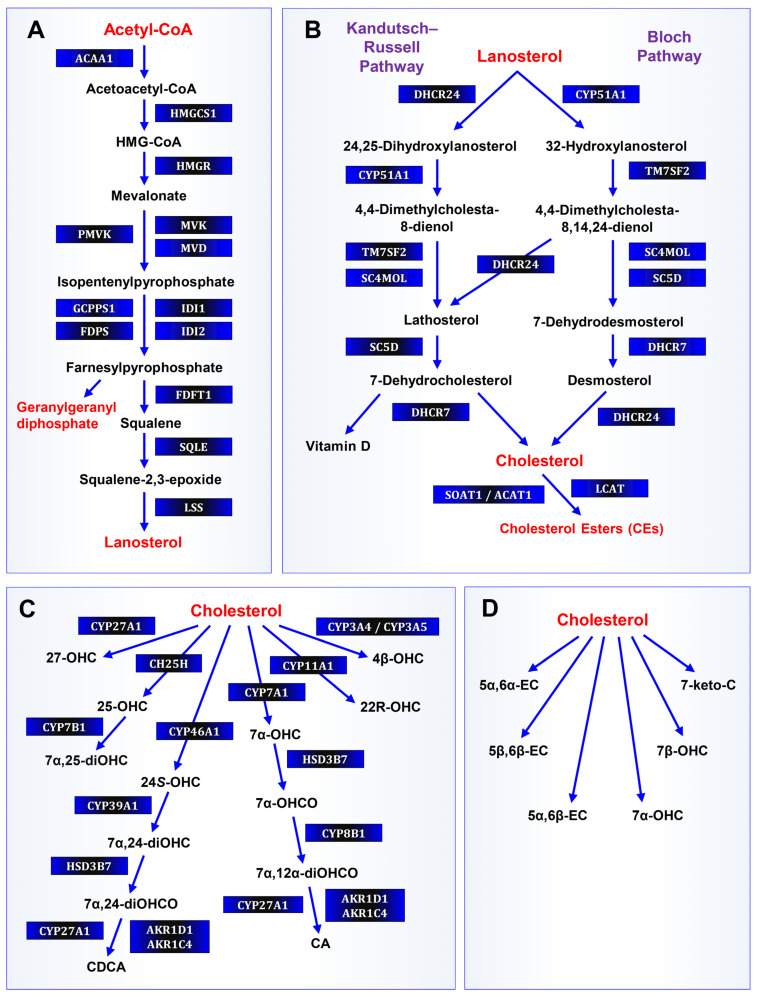
Cholesterol biosynthetic and metabolic pathways. (**A**). **De novo cholesterol biosynthesis (pre-squalene mevalonate pathway)**. **Acetyl-CoA**: acetyl-coenzyme A; **ACAA**: acetyl-coenzyme A acetyltransferase; **acetoacetyl-CoA**: acetoacetyl-coenzyme A; **HMGCS1**: 3-hydroxy-3-methylglutaryl-coenzyme A synthase 1; **HMG-CoA**: 3-hydroxy-3-methylglutaryl-coenzyme A; **HMGR**: HMG-CoA reductase; **PMVK**: phosphomevalonate kinase; **MVK**: mevalonate kinase; **GGPPS1**: geranylgeranyl diphosphate synthase 1; **IDI1**: isopentenyl-diphosphate delta isomerase 1; **FDPS**: farnesyl-diphosphate synthase; **IDI2**: isopentenyl-diphosphate delta isomerase 2; **FDFT1**: farnesyl-diphosphate farnesyltransferase 1; **SQLE**: squalene epoxidase; **LSS**: lanosterol synthase. (**B**). **De novo cholesterol biosynthesis (post-squalene mevalonate pathway, including the Bloch and Kandutsch–Russell pathways) and cholesterol esterification**. **DHCR24**: 24-dehydrocholesterol reductase; **CYP51A1**: cytochrome P450 51A1; **24,25 DHLan**: 24,25-dihydrolanosterol; **TM7SF2**: transmembrane 7 superfamily member 2; **SC4MOL**: methylsterol monooxygenase 1; **SC5D**: sterol-C5-desaturase; **DHCR7**: 7-dehydrocholesterol reductase; **SOAT1** (also called **ACAT1**): sterol O-acyltransferase 1 (acyl-CoA:cholesterol acyltransferase 1); **LCAT:** lecithin:cholesterol acyltransferase. (**C**). **Enzymatic cholesterol catabolism**. **CYP27A1**: cytochrome P450 27A1; **CYP3A4** or **CYP3A5**: cytochrome P450 3A4 or 3A5, respectively; **4β-OHC**: 4β-hydroxycholesterol; **27-OHC**: 27-hydroxycholesterol; **CH25H**: cholesterol 25-hydroxylase; **CYP11A1**: cytochrome P450 11A1; **22*R*-OHC**: 22*R*-hydroxycholesterol; **25-OHC**: 25-hydroxycholesterol; **CYP7B1**: cytochrome P450 7B1; **7α,24-diOHC**: 7α,24-dihydroxycholesterol; **CYP46A1**: cytochrome P450 46A1; **CYP7A1**: cytochrome P450 7A1; **CYP7B1**: cytochrome P450 7B1; **24S-OHC**: 24*S*-hydroxycholesterol; **CYP39A1**: cytochrome P450 39A1; **7α-OHC**: 7α-hydroxycholesterol; **CYP8B1**: cytochrome P450 8B1; **7α,12α-diOHCO:** 7α,12α-dihydroxycholestenone; **HSD3B7**: 3β-hydroxysteroid dehydrogenase type 7; **7α-OHCO**: 7α-hydroxycholestenone; **CA**: cholic acid; **CDCA**, chenodeoxycholic acid. (**D**). **Non-enzymatic cholesterol catabolism**. **7β-OHC**: 7β-hydroxycholesterol; **5α,6α-EC**: 5α,6α-epoxycholesterol; **5β,6β-EC**: 5β,6β-epoxycholesterol; **5α,6β-EC**: 5α,6β-epoxycholesterol.

Provided below is an explanation of each of the hypothetical elements outlined above, along with a critical analysis of the available supporting evidence, most of which is scattered in the unrelated scientific literature in bits and pieces. In addition, efforts are made to apply the newly developed hypothesis to provide a better mechanistic explanation for some of the interesting, yet poorly understood, experimental and/or clinical observations related to AD (mostly LOAD).

## 4. Pathogenic Roles of Neuronal Cholesterol Dysregulation in LOAD

### 4.1. A Brief Review of Cholesterol in Normal Brain Function

A brief review of the relevant knowledge points related to the synthesis, regulation and function of cholesterol in the CNS is provided in this section; it is intended to help non-expert readers more readily understand the proposed hypothesis and the relevant explanations provided in later sections.

*Brain cholesterol content.* The human brain is enriched with cholesterol compared with other tissues. It is estimated that while the brain only makes up, on average, 2.1% of body mass, it contains ~23% of total body cholesterol [58,59]. The cholesterol levels in most animal tissues are around 2 mg/g tissue, but its level in the brain is 15–20 mg/g tissue [58].

Most of the cholesterol (70–90%) in the CNS is associated with myelin surrounding the axons. The highest biosynthesis of cholesterol is found in oligodendrocytes in the brain during developmental stages (which involves active myelination) and decreases by ~90% in adults after myelination is completed [60].

It is generally thought that due to the presence of the blood–brain barrier (BBB), CNS cholesterol does not readily equilibrate with lipoprotein-associated cholesterol in the periphery [61,62]. Therefore, cholesterol in the brain is believed to be produced locally [58,63], and is synthesized primarily in glial cells, such as astrocytes and oligodendrocytes, although cholesterol can also be synthesized in smaller quantities in neurons [47,63]. Cholesterol transport among different cell types in the CNS is mostly carried out by ApoE-containing lipoproteins [64] (discussed in detail in Section 5.1).

Cholesterol serves as a precursor for the synthesis of neurosteroids, oxysterols and bile acids in the brain [65,66,67]. Importantly, cholesterol also functions as a membrane reinforcer, regulating neuronal signaling via lipid rafts. The fraction of cholesterol that binds tightly with sphingolipids forms a sterol-/sphingolipid-rich domain, commonly referred to as the lipid raft domain, serves as the platform to host various membrane proteins involved in cell signaling and neurotransmission [68,69]. Notably, the amyloid precursor protein (APP) is also richly contained in the lipid raft domain, where enzymatic cleavage of APP by different secretases takes place [70]. In fact, it is known that increasing neuronal cholesterol level can alter APP cleavage, favoring the formation of A*β*_42_ and A*β*_40_ fragments (discussed in detail in Section 6).

*Brain cholesterol synthesis and regulation.* Cholesterol synthesis in the brain is precisely regulated [49,52]. Cholesterol is synthesized from acetyl-CoA via a complex pathway involving over 30 enzymatic steps (depicted in Figure 1 and Figure 2). Acetyl-CoA, which is transported from mitochondria to cytoplasm, is the building block for cholesterol synthesis. The enzyme acetyl-CoA acyltransferase (ACAA) uses acetyl-CoA to generate acetoacetyl-CoA, which is then converted to HMG-CoA by HMG-CoA synthase. The next step is the reduction in HMG-CoA to mevalonate (or mevalonic acid), which is catalyzed by HMG-CoA reductase (HMG-CoA-R or simply as HMGR), a rate-limiting step in sterol synthesis. Mevalonate contains five carbons and is converted via a series of reactions to lanosterol, which is a sterol precursor with 30 carbons. After going through additional enzymatic reactions at the endoplasmic reticulum (ER) membrane, lanosterol is converted to cholesterol (27 carbons). In addition, mevalonate is a precursor for the synthesis of geranylgeraniol, which is needed for covalent modifications of many neuronal proteins via enzymatic prenylation [51]. Mevalonate and geranylgeraniol are considered crucial neuroactive metabolic intermediates [53] and play an important role in learning and memory formation (discussed later).

Feedback control of cholesterol synthesis includes both sterol and nonsterol-mediated degradation of HMGR (Figure 1; reviewed in [71,72]). HMGR is a multi-span membrane protein in ER, and its degradation occurs when sterols accumulate in ER membranes. Sterols will cause the binding of HMGR to a pair of ER membrane proteins (Insig-1 and Insig-2), and Insig binding leads to ubiquitination of HMGR, which then undergoes proteasome-mediated degradation in the cytosol.

In addition to sterol’s regulation of HMGR degradation via Insig proteins, sterol-dependent transcription factor (SREBP2) is another sterol-dependent regulator, causing down-regulation of genes involved in both sterol synthesis (e.g., HMGR) and transport (e.g., LDL receptor) [73].

Geranylgeraniol, which is a nonsterol formed during cholesterol synthesis, can also exert a feedback inhibition of HMGR. Feedback inhibition of HMGR by geranylgeraniol provides a precise additional regulation of the cholesterol synthesis pathway when it is activated solely for the purpose of producing the neuroactive metabolic intermediates (mavelonate and geranylgeraniol) but not for the synthesis of more cholesterol.

Additionally, the HMGR activity can be further regulated by metabolic states in other sterol-independent manners as follows: the enzyme can undergo phosphorylation or dephosphorylation in a reversible manner via the actions of protein kinases and phosphoprotein phosphatases, which results in enzyme inactivation and activation [74].

**Figure 3:**
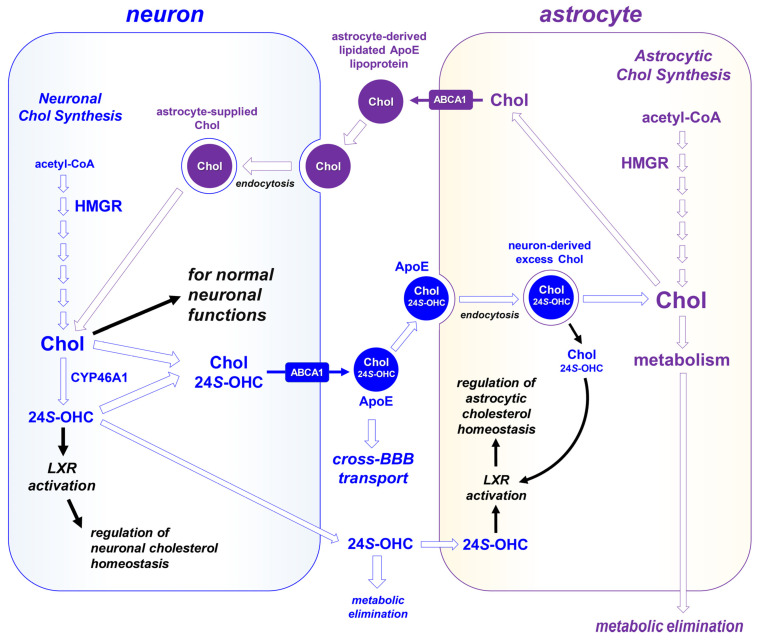
Regulation of cholesterol synthesis and transport in brain neurons and astrocytes. As depicted in the right panel, astrocytes are the main site in the brain for the de novo synthesis of cholesterol (abbreviated as Chol) using acetyl-CoA as the starting material. In addition, brain neurons also have the ability to perform de novo synthesis of a smaller amount of cholesterol using acetyl-CoA as the starting material (depicted in the left panel). Brain ApoE-containing lipoproteins are the main carriers that can transport astrocyte-derived lipids (rich in cholesterol and CEs) to neurons through ApoE receptor-mediated endocytosis. Once internalized, ApoE lipoprotein particles supply cholesterol (and other lipids) to neurons, which are usually required for fulfilling certain neuronal functions. Importantly, ApoE can also efflux excess neuronal cholesterol to astrocytes for disposal in an ABCB1-dependent manner. As depicted, neurons selectively express CYP46A1; when neuronal cholesterol supply is in excess, CYP46A1 expression will be upregulated, which will convert cholesterol to 24*S*-OHC. 24*S*-OHC is a crucial neuronal regulator, which can activate neuronal LXR to regulate neuronal cholesterol homeostasis. Specifically, under the feedback regulation of 24*S*-OHC, neurons will stop endocytosing astrocyte-produced, cholesterol-rich ApoE particles, and in the meantime, they will enhance the efflux of excess neuronal cholesterol in an ABCA1-/ApoE-dependent manner. While this effluxed neuronal cholesterol (in ApoE lipoprotein particles) can be taken up by astrocytes for metabolism and disposal, it can also undergo cross-BBB transport (discussed in Section 9.1). As an important part of the feedback regulation, the neuronally produced 24S-OHC can be transported to the neighboring astrocytes (via simple diffusion or ApoE-/ABCA1-dependent efflux), which will then activate the LXR in astrocytes to suppress the astrocytic synthesis and release cholesterol and will also enhance cholesterol metabolism and disposition by astrocytes.

*Sources of neuronal cholesterol and its intracellular trafficking.* As depicted in Figure 3, cholesterol in brain neurons is synthesized primarily in glial cells (e.g., astrocytes and oligodendrocytes) [44,45,51], although a smaller quantity of cholesterol can also be synthesized in neurons [47,52]. Cholesterol transport among different cell types in the CNS is mostly carried out by ApoE-containing lipoproteins [53] (discussed in Section 5.1). The movement of cholesterol through different subcellular compartments inside a neuron involves multiple metabolic pathways, resulting in different intracellular cholesterol pools that are in slow equilibrium with one another.

***i.*** *Supply by cholesterol-rich lipoproteins.* In the brain, ApoE is a major cholesterol carrier, and astrocyte-derived ApoE lipoproteins enter a neuron through receptor-mediated endocytosis [49,52,75] (Figure 3). After endocytosis, lipoproteins first enter a distinct early endocytic compartment enriched with acid lipase for hydrolysis of cholesterol esters (CEs) [76]. Cholesterol released from lipoprotein-derived CEs will move to endosomes which contain a pair of cholesterol-binding proteins called Niemann–Pick type C1 (NPC1) and NPC2. NPC2 (a soluble protein located in the luminal side of endosomes and lysosomes [77]) binds cholesterol for its transfer to NPC1 [78,79], a protein with multiple transmembrane domains, including a sterol-sensing domain [80]. NPC1 then exports cholesterol to the exterior of late endosomes and lysosomes. Cholesterol exiting from the late endosomes will end up in other membrane compartments, including the plasma membrane, ER, trans-Golgi network, mitochondria, and peroxisomes [81].

***ii.*** *De novo biosynthesis.* Inside a neuron, the newly synthesized sterols, including cholesterol, lanosterol and other precursor sterols, move quickly from the ER to the plasma membrane. Upon arriving at the plasma membrane, part of the synthesized sterols is released to the exterior by ABCA1 and apolipoprotein-dependent sterol efflux [82,83]. Those sterols (i.e., cholesterol, lanosterol and other precursor sterols) remaining at the plasma membrane will recycle between the plasma membrane and various internal compartments, including endosomes and lysosomes [84,85].

***iii.*** *SOAT1-mediated esterification.* Cholesterol inside a neuron is also a substrate for sterol *O*-acyltransferase 1 (SOAT1), which is also called acyl-CoA:cholesterol acyltransferase 1 (ACAT1), resulting in the formation of CEs (Figure 2B). CEs are then sequestered in cellular lipid droplets, which are subject to hydrolysis by enzymes collectively designated as CE hydrolases [86]. When SOAT1 is inhibited, part of the cholesterol pool destined for storage as CEs may be shuffled to the plasma membrane where cholesterol may serve as substrate for ABCA1-mediated efflux [83].

***iv.*** *Cholesterol in mitochondria.* Mitochondrial cholesterol comes from at least three sources as follows: the first source is the plasma membrane. While the mechanism of the plasma membrane–mitochondria cholesterol movement is presently still unclear, this process does not depend on NPC2 [87]. When reaching the mitochondria, the transfer of cholesterol from the outer membrane to the inner membrane is largely mediated by the StAR protein (steroidogenic acute regulatory protein) [88]. The second source is from late endosomes and lysosomes. In cells with low levels of StAR protein, the STARD3 (StAR-related lipid transfer protein domain 3) in the late endosomes [89,90] works along with NPC2 to transport cholesterol from the late endosomes and lysosomes to the mitochondria [91,92]. This process may explain that in cells with mutant NPC1, cholesterol overloading will result in cholesterol buildup in mitochondria [93,94]. This information aids in the understanding that mitochondrial toxicity and cellular ATP depletion are crucial causative changes in NCP disease (discussed in Section 9.4). The third source of mitochondrial cholesterol comes from a specialized membrane region designated as the mitochondria-associated membranes, which are part of the ER membranes in close physical contact with the mitochondrial membrane [95]. These membranes are enriched with cholesterol and SOAT1 (the enzyme that converts cholesterol to CEs) [96,97].

*Cholesterol in normal brain functions.* Cholesterol is involved in many important brain functions, such as synthesis of neurosteroids [65,66,67], learning and memory formation [33,34,35,36], synaptogenesis [37,38] and axonal growth [98]. Being an important component of the plasma membrane, cholesterol is also involved in regulating ion permeability [99] and signal transduction in neurons [100]. An earlier in vitro study showed that cholesterol depletion in cultured rat hippocampal neurons with methyl-*β*-cyclodextrin (a cholesterol-sequestering agent) reduced excitatory postsynaptic currents (EPSCs) and long-term potentiation (LTP) [35]. Other similar studies also reported that dysregulation of brain cholesterol homeostasis affects synaptic functions [101,102,103].

In the brain, astrocytes are the main source of neuronal cholesterol [39] (depicted in Figure 3). Selective loss of astrocytic cholesterol synthesis alters brain development and synaptic functions in vivo, with reduced synaptic vesicle numbers and defective synaptic plasticity [43,44]. As cholesterol is enriched in myelin [104] and required for its growth [105,106], oligodendrocytes support neuronal function partly by enwrapping neuronal axons with cholesterol-enriched myelin.

### 4.2. Review of Evidence for Neuronal Cholesterol Dyshomeostasis in LOAD

As cholesterol has important functions in the brain, some of its neuronal functions will be compromised if the supply of astrocyte-derived cholesterol is severely inadequate (more discussion on this subject is provided later). However, when cholesterol level inside neurons is abnormally elevated, it will also become pathogenic. Human studies have shown that elevated blood cholesterol is associated with increased LOAD risk in the elderly [14,15,16,17,18]. Similarly, cholesterol metabolism in LOAD brains is altered compared to normal brains [16,19,20], and alterations in brain cholesterol metabolism are associated with increased LOAD risk [18,20,23].

Consistent with the human observations, animal studies have more clearly demonstrated that feeding rabbits a cholesterol-rich diet can cause learning and memory impairment, along with increased A*β* production in their brains [24].

The link between disrupted cholesterol homeostasis in the CNS and neurodegeneration is perhaps best exemplified in the NPC disease, which results from mutations in either NPC1 or NPC2 gene. NPC1 and NPC2 each can bind cholesterol and act in tandem in late endosomes/lysosomes to mediate the exit of CEs derived from endocytosed lipoproteins [107,108]. In NPC1- or NPC2-deficient neurons [109] and glial cells, unesterified cholesterol and other lipids will be sequestered in late endosomes/lysosomes, and the amount of cholesterol in the plasma membrane and ER (the cellular sites at which cholesterol homeostasis is regulated) is reduced. In the *NPC1*^−/−^ neurons, it was reported that there is an increase in cholesterol content in neuronal cell bodies (especially in the mitochondria) but a decrease in cholesterol content in the distal axons [109,110].

While the NCP disease is a relatively rare inherited disorder (autosomal recessive) in humans, it causes progressive neurodegeneration and premature death (along with hepatosplenomegaly and lung disease) [111]. A characteristic histological finding in the brain is a massive loss of neurons, particularly cerebellum Purkinje cells, which is consistent with the impairment of motor functions [112], although neurons in other parts of the brain are also affected to varying degrees. The precise mechanism underlying the pathogenesis of the NCP disease is still not fully understood at present, but one thing is certain—dysregulation of the neuronal cholesterol homeostasis (e.g., transport) is a critical pathogenic change that directly results in massive neuronal injury (a more detailed mechanistic explanation on the NCP disease is provided in Section 9.4).

In addition to AD and NPC disease, other neurodegenerative diseases (e.g., Huntington’s disease and Smith–Lemli–Opitz syndrome) are also associated with cholesterol dyshomeostasis [113,114]. The apparent link between these disease conditions and neuronal cholesterol abnormality underscores the clinical relevance of the proposed cholesterol-centered hypothesis on pathogenesis. A mechanistic explanation on the pathogenic role of cholesterol in LOAD is provided below.

### 4.3. Mechanistic Explanation: How Does Neuronal Cholesterol Dysregulation Contribute to LOAD Pathogenesis?

In addition to its involvement in neurosteroid synthesis [65,66,67], synaptogenesis [37] and axonal growth [38,98], cholesterol also has a unique role in regulating neuronal membrane functions, including membrane fluidity, vesicle formation and fusion, ion channel function, and formation of specialized microdomains involved in neural communication [115,116]. Indeed, cholesterol is required presynaptically for formation of neurotransmitter vesicles [117], and postsynaptically for clustering neurotransmitter receptors [115]. As change in the strength of synaptic connections may alter learning and memory formation [33,34], dysregulation of neuronal cholesterol homeostasis has been suggested to affect learning and memory through modulating the presynaptic and/or postsynaptic processes of neurotransmission [20]. Furthermore, neuronal cholesterol abnormality is known to alter enzymatic formation of A*β* peptides and amyloid plaques [26,27,28,29,30].

In this paper, a new hypothesis is proposed, which suggests that in most cases of LOAD, abnormally elevated neuronal cholesterol constitutes a major causative factor, which drives the pathogenic processes of LOAD. The elevated neuronal cholesterol (particularly mitochondrial cholesterol) will disrupt mitochondrial structure and metabolic activity, resulting in reduced synthesis of ATP and important neuroactive metabolic intermediates required for learning and memory formation. These metabolic changes are important initial events that will trigger a series of pathogenic changes culminating in the gradual development of LOAD (depicted in Figure 1). Specifically, elevated neuronal cholesterol will increase the formation of A*β*_42_ and A*β*_40_ (discussed in Section 6); neuronal ATP deficiency will enhance tauopathy (discussed in Section 7) and decrease the formation of cholinergic vesicles (discussed in Section 8). Additionally, elevated brain cholesterol will suppress macrophage response (due to reduced glial ATP levels), which contributes to the development of neuroinflammation in LOAD.

Offering partial support for this hypothesis, a recent study has shown that exposure of cultured neuronal cells to even very low concentrations of free cholesterol can disrupt mitochondrial structure and metabolic activity (for ATP synthesis) [32]. These observations agree with the results of an earlier study that used a genetic mouse model with a selective mitochondrial cholesterol overload to examine its effect on A*β* neurotoxicity and AD pathology [118]. Additionally, it was shown earlier [118] that the isolated mitochondria from the cortical neurons of NPC1-deficint transgenic mice exhibited mitochondrial cholesterol accumulation, glutathione (GSH) decrease, and increased susceptibility to A*β*_42_-induced oxidative stress and cell death. More importantly, in a transgenic AD mouse model, it was reported earlier that there were clear early deficits in synaptic mitochondrial functions [119], likely resulting from neuronal cholesterol dysregulation.

As mentioned earlier, learning and memory formation requires mevalonate, which serves as a precursor for the subsequent synthesis of geranylgeraniol (required for protein prenylation in neurons [51]). Both mevalonate and geranylgeraniol are important neuroactive metabolic intermediates formed during cholesterol biosynthesis [50] (Figure 1). Elevated neuronal cholesterol will inhibit the cholesterol synthesis pathway, which will then reduce the synthesis of these key neuroactive metabolic intermediates. In support of this hypothesis, an earlier animal study showed that the learning and memory function can be restored in an AD model by giving geranylgeraniol [50].

Based on the above discussion, it is understood that the upper half of the cholesterol synthesis pathway in brain neurons needs to be kept active during the normal process of memory formation as it produces the neuroactive metabolic intermediates required for the formation of new synaptic connections. As such, even if cholesterol is not needed by the neurons (for instance, when cholesterol is already adequately supplied by the neighboring astrocytes), the upper half of the cholesterol synthesis pathway in neurons may still need to remain active, which is not for the purpose of synthesizing more cholesterol, but for the synthesis of neuroactive metabolic intermediates (Figure 1).

In order to keep the upper half of the cholesterol synthesis pathway active in brain neurons, it is important to keep free cholesterol levels relatively low in these cells basically all the time. When neuronal cholesterol level is abnormally elevated, it will suppress the cholesterol synthesis pathway through feedback inhibition of HMGR. Notably, statins are known to exert their cholesterol-lowering effect by inhibiting HMGR. Based on the above discussion, it is understood that centrally active statins will not be as beneficial (most likely harmful) for AD as they will always concomitantly reduce the levels of both cholesterol and neuroactive metabolic intermediates (mevalonate and geranylgeraniol) in brain neurons. While reductions in neuronal cholesterol likely will be beneficial most of the time, the reduced synthesis of neuroactive metabolic intermediates in neurons will always be harmful for learning and memory formation. Based on the available clinical observations, the net effect of centrally active statins is mixed, likely depending on the statin doses used and the degree of neuronal HMGR inhibition produced. While most studies reported a lack of improvement in memory function with centrally active statins, it appears that the use of peripherally acting statins is more likely to have a beneficial effect. More detailed discussion on the complex effects of statins in LOAD is provided in Section 9.3.

Based on the above explanation, it is also understood that alterations in many factors that affect cholesterol metabolism and transport will also alter the risk for LOAD. For instance, CYP46A1, which is also called “cholesterol 24(*S*)-hydroxylase” [120,121], catalyzes the conversion of cholesterol to 24(*S*)-hydroxycholesterol (24*S*-OHC) [122,123], which is the most abundant oxysterol found in human brain [124]. This metabolic pathway plays a crucial role in regulating neuronal cholesterol disposition and homeostasis, and abnormalities are associated with an increased risk for LOAD [124,125]. Similarly, alterations in factors like ApoE4, ABCA1 and ApoE receptors, which are involved in cholesterol transport (particularly neuronal cholesterol efflux), and SOAT1, which catalyzes CE formation, will also alter the risk of LOAD. Provided below is a discussion of the roles of CYP46A1 and SOAT1 in cholesterol homeostasis and their association with LOAD, while the discussion of other relevant factors (such as ApoE4, ABCA1, lipoprotein receptors, etc.) and their respective pathogenic contribution to LOAD is provided separately in relevant later sections.

*Mechanistic explanation of the role of CYP46A1 in LOAD.* Neurons are the primary cell type in the brain that expresses CYP46A1 [125]. CYP46A1 oxidizes cholesterol to 24*S*-OHC and helps maintain cholesterol homeostasis in the brain [122]. It was suggested by some researchers that for cholesterol to be transported across the BBB, it needs to be first converted to 24*S*-OHC by CYP46A1 [126]. 24*S*-OHC then diffuses out of cells and crosses the BBB and is finally cleared in the liver [127]. In addition, it has also been suggested that 24*S*-OHC can be removed from brain neurons in an ABCA1-/ApoE-dependent manner (depicted in Figure 3, *left panel*). As discussed below, the newly proposed hypothesis (i.e., elevated neuronal cholesterol is a key pathogenic factor in LOAD) will help better understand the role of CYP46A1-mediated cholesterol metabolism in both AD animal models and LOAD patients.

***i.*** It was reported [128] that the adenovirus-mediated selective overexpression of *CYP46A1* in AD animal models reduced AD severity, such as memory and learning impairment and amyloid accumulation. Similarly, it is predicted that selective knockdown of *CYP46A1* will increase AD severity in experimental AD animal models (if we assume that everything else is unchanged). In fact, the observations made with rat and human primary brain neurons in culture agreed with the prediction that neuronal CYP46A1 level is associated with the risk of AD [129].

The mechanistic explanation for these experimental observations or predictions is quite straightforward. The protective effect of the CYP46A1-mediated cholesterol metabolism in AD animal models likely results from the following two mechanisms: one is CYP46A1-mediated metabolic disposition of excess neuronal cholesterol. As CYP46A1 is selectively expressed in brain neurons, animals with *CYP46A1* knockdown are expected to have a drastically reduced ability to metabolize neuronal cholesterol and thus will result in higher levels of cholesterol inside brain neurons. Alternatively, if the brain neurons have a high ability to metabolize cholesterol, then neuronal cholesterol levels will be reduced, which is beneficial for neuronal survival. The other mechanism is related to the activation of the nuclear receptor LXR system by 24*S*-OHC [130], which is formed by CYP46A1. Activation of LXR by 24*S*-OHC will regulate a number of genes associated with cholesterol homeostasis [130,131], such as reduced expression of HMGR (which reduces cholesterol synthesis) and increased expression of cholesterol efflux transporters and ApoE (which jointly mediate cholesterol efflux).

***ii.*** While the above explanation of the results from animal studies is quite straightforward and readily understood, the results from clinical studies are more complex than what is observed in animal models. Many earlier human studies reported that elevated 24*S*-OHC levels in cerebrospinal fluid (CSF) were associated with neurodegenerative diseases (reviewed in [132]). For instance, studies have shown that CSF 24*S*-OHC levels are higher in LOAD patients, in patients with vascular dementia, and in patients with mild cognitive impairment compared to the control subjects [133,134,135]. The higher CSF 24*S*-OHC levels in LOAD patients [133,135] were also correlated with elevated CSF levels of ApoE, cholesterol and tau [127,132]. In these clinical studies, there was a clear positive correlation between increased levels of 24*S*-OHC and the risk of LOAD, which gives a false impression that 24*S*-OHC is a causative factor in LOAD. For a better understanding of these human observations, a bit more explanation is needed here. In most LOAD patients, it is expected that their average neuronal cholesterol levels are higher than those of non-demented healthy individuals. The higher neuronal cholesterol levels will lead to increased metabolic disposition catalyzed by CYP46A1 (along with other metabolic pathways). As a result, the oxysterol metabolites are higher in their CSF as well as blood circulation, which actually reflects an enhanced effort of the LOAD patients’ bodies to dispose of excess neuronal cholesterol. Here, it should be noted that the observed higher levels of 24*S*-OHC found in the CSF and/or blood of LOAD patients compared to non-demented human subjects are largely because a majority of the LOAD patients are expected to have higher levels of neuronal (and blood) cholesterol to begin with (i.e., before the onset of clinical LOAD) compared to non-demented control subjects. If two groups of LOAD patients (or two groups of non-demented control subjects) with the same initial neuronal cholesterol levels are compared, it is quite certain that the expected observations will be opposite, i.e., those who have a higher capability to metabolically convert neuronal cholesterol to 24*S*-OHC (i.e., with higher neuronal CYP46A1 activity) will have a lower risk for developing LOAD.

***iii.*** In addition to CYP46A1-mediated formation of 24*S*-OHC, there are other enzymes also capable of catalyzing the conversion of cholesterol to other oxysterols (e.g., 27-hydroxycholesterol) and bile acids (Figure 2C). Based on the understanding that elevated neuronal cholesterol is a causative factor in LOAD, it is readily understood that increased metabolic conversion of neuronal cholesterol to other oxysterols and bile acids will also be beneficial for reducing AD risk in animal models, which will be similar to the beneficial effects of enhanced metabolic conversion of cholesterol to 24*S*-OHC seen in animal models (as discussed above).

However, clinical studies again reported an opposite finding, i.e., there appeared to be a “positive correlation” between brain 27-hydroxycholesterol level and LOAD risk [136]. The levels of 27-hydroxycholesterol in the blood, brain and CSF were found to be markedly elevated in individuals with LOAD [136]. These observations are very similar to the situation with 24*S*-OHC as discussed above. It is speculated that the increased blood levels of 27-hydroxycholesterol only indirectly reflect an enhanced effort of the LOAD patient’s body to metabolically dispose of elevated neuronal cholesterol.

In addition, alterations in brain levels of nonenzymatically produced oxysterols were also observed in LOAD, which include 7*α*-hydroxycholesterol (this oxysterol can also be formed enzymatically by CYP7A1) [137], 7*β*-hydroxycholesterol, 5*α*,6*α*-epoxycholesterol, 5*α*,6*β*-dihydroxycholestanol, and 5*β*,6*β*-epoxycholesterol (Figure 2D). These observations would reflect altered levels of free, unmetabolized cholesterol available for both enzymatic and nonenzymatic conversion to various oxysterol derivatives in these patients.

*Mechanistic explanation of the role of SOAT1 in LOAD.* In addition to the conversion of cholesterol to oxysterols, a fraction of cholesterol is esterified for storage by the following two enzymes: SOAT1 and LACT (lecithin:cholesterol acyltransferase) [138,139]. Notably, an earlier animal study showed that when a mouse model of AD was cross-bred with mice lacking *SOAT1*, the amyloid pathology was attenuated in these animals [140]. Similarly, human studies have shown that polymorphism in *SOAT1* gene was associated with reduced brain amyloid load and reduced CSF cholesterol content [141], whereas elevated *SOAT1* expression was associated with more severe amyloid load [142].

How to explain the observed relationship between SOAT1 inhibition and reduced amyloid load? As explained later in Section 6, enhanced cleavage of APP by *β*-/*γ*-secretases resulting in the production of A*β*_42_ and A*β*_40_ (major fragments contained in amyloid plaques) will mostly take place in the lipid raft regions of the cell membrane when neuronal cholesterol is elevated. The above experimental observations suggest that the form of cholesterol contained in the lipid rafts of neurons is mostly CEs rather than unmetabolized free cholesterol. When SOAT1 is absent, the content of CEs in the lipid rafts will not increase in proportion to cellular free cholesterol and thus will not lead to a proportional increase in APP cleavage through the *β*-/*γ*-secretase pathways and a more severe amyloid pathology. A net increase in the cellular free cholesterol pool will naturally lead to increased metabolic disposition by CYP46A1 (due to increased availability of the substrate), thus resulting in increased formation of 24*S*-OHC [140]. Increased formation of 24*S*-OHC will then activate the expression of other genes that will further help reduce neuronal cholesterol. In addition, an increase in cellular free cholesterol pool will serve as substrate for ABCA1-mediated lipid efflux [83]. However, it should be noted that learning and memory performance of SOAT1-deficient transgenic mice (which have a markedly reduced amyloid pathology) may not be proportionally better as the levels of free cholesterol inside their brain neurons will not be markedly improved. If free cholesterol level in neurons remains highly elevated, which will disrupt mitochondrial function and inhibit the synthesis of ATP and neuroactive metabolic intermediates, it is expected that learning and memory performance of these animals will still be impaired.

Notably, inhibition of SOAT1 enzymatic activity has received attention as a potential therapeutic strategy in LOAD as it can reduce amyloidogenic processing of APP through reducing the formation of CEs plus potentially increasing the conversion of unesterified cholesterol to 24*S*-OHC by CYP46A1 [140]. However, the real therapeutic benefits of SOAT1 inhibitors used alone are expected to be limited since, as discussed above, the free cholesterol level in brain neurons may not be proportionally reduced as a result of SOAT1 inhibition alone.

### 4.4. Section Summary

As cholesterol has important physiological functions in the brain, lack of adequate cholesterol supply will impede certain neuronal functions. However, from a pathogenic perspective, it is hypothesized that it is equally or even more important when cholesterol level in neurons is abnormally elevated, as cholesterol can readily disrupt mitochondrial structure and function and inhibit the synthesis of ATP and neuroactive metabolic intermediates [32]. In addition, elevated neuronal cholesterol is a major cause for increased formation of A*β*_42_ and amyloid plaques (discussed later in Section 6), and a reduction in neuronal ATP will lead to the development of tauopathy (discussed in Section 7.2) and reduced formation of cholinergic vesicles (discussed in Section 8.2). Therefore, a new hypothesis is proposed here which speculates that in most cases of LOAD, chronically elevated cholesterol inside brain neurons constitutes a major causative factor, which drives many key aspects of the pathogenic process (depicted in Figure 1). There is mounting evidence from human epidemiological studies as well as experimental studies using both animal models and cultured neuronal cells which jointly demonstrate that elevated neuronal cholesterol is associated with mitochondrial inadequacy, neuronal injury, and increased AD risk. In line with this mechanistic suggestion, earlier studies in a mouse AD model showed that, in the early stage of AD development, synaptic mitochondria exhibit significant functional deficits, and the degree of deficits correlates positively with the degree of A*β* accumulation [119,143].

Based on the proposed cholesterol-centered hypothesis, it is speculated that neuronal cholesterol is elevated in LOAD patients, which is an important initial change. Subsequently, elevated cholesterol will suppress the cholesterol synthesis pathway in neurons and will lead to increased metabolic formation of 24S-OHC, which will suppress astrocytic cholesterol synthesis, along with increased efflux of cholesterol out of neurons and across the BBB (discussed in Section 9.1). As a result of these feedback regulations, the net cholesterol level in the brains of LOAD patients often may not be overly too high compared to non-demented control subjects. Here, it is important to mention that while this might be the case, the neuroactive metabolic intermediates in these patients are likely markedly reduced compared to non-demented individuals. The main reason for their decrease is because their synthesis will be suppressed most of the time due to the presence of feedback inhibition by elevated neuronal cholesterol. A recent targeted metabolomic study demonstrated that the concentration of the cholesterol precursor lanosterol, but not free cholesterol, was clearly lower in the brains of LOAD patients than in the brains of control subjects [142]. Similar observations were made with the levels of major enzymes involved in cholesterol synthesis [142], indicating a decrease in these cholesterol-synthesizing enzymes in relevant brain regions of LOAD patients. Notably, the expression of these enzymes was not similarly altered in the substantia nigra of Parkinson’s patients, suggesting that these changes are relatively specific to the brain regions of LOAD patients [142].

Lastly, it is known that astrocytes are the main site for cholesterol synthesis in the brain. An earlier animal study reported that while selective loss of brain astrocytic cholesterol synthesis in vivo significantly alters brain development, the learning ability of these mice appeared to be largely unaffected (although their memory is reduced) [43]. This observation is intriguing and suggests that the lack of astrocytic cholesterol supply mostly affects brain development, a process that heavily involves myelination and requires a lot of astrocyte-supplied cholesterol but not learning ability. In fact, the lack of astrocytic supply of cholesterol will reduce neuronal cholesterol levels, which may actually help maintain a higher level of ATP and neuroactive metabolic intermediates in these neurons, and these changes supposedly would be beneficial for the learning process. On the other hand, the long-term memory formation in these animals is still affected, likely resulting from the lack of astrocytic supply of cholesterol which is required for learning-induced de novo myelination, an essential process in long-term memory formation [144].

## 5. How Do Different ApoE Isotypes Contribute to LOAD Pathogenesis?

### 5.1. A Brief Overview of ApoE in Normal Brain Function

Human ApoE is a 34-kD glycoprotein present in the CNS and periphery, serving as a lipid carrier [145,146]. It consists of 299 amino acids, encoded by the *APOE* gene (on chromosome *19q13.32*). Human ApoE is polymorphic, with three major alleles, *APOE-ε2* (ApoE2: cys112, cys158), *APOE-ε3* (ApoE3: cys112, arg158), and *APOE-ε4* (ApoE4: arg112, arg158) [147,148].

In the periphery, ApoE is synthesized and secreted mostly by hepatocytes and macrophages, and is associated with various lipoproteins, ranging from small plasma HDL particles (7–14 nm in size) [149] to the larger polyhedral VLDL particles (30–100 nm in size) [92], to the very large chylomicrons (75–1200 nm) [150,151,152]. Its main function is to transport lipids and regulate plasma lipid levels and is also involved in immune modulation [153,154].

In the CNS, ApoE is the most abundant apolipoprotein [145], although other apolipoproteins are also present, including the more abundant ApoA-I and the less abundant apolipoproteins such as ApoJ, ApoA-II, ApoA-IV, ApoD and ApoH [41]. The majority of ApoE is believed to be produced in astrocytes, while microglia, vascular mural cells, choroid plexus and neurons can also produce ApoE [145,155].

One of the best-studied functions of brain ApoE proteins is their transport of astrocyte-derived lipids (rich in cholesterol and CEs) to neurons through ApoE receptor-mediated endocytosis [145,156]. A number of ApoE receptors have been identified, and they belong to the LDL receptor family, such as the LDL receptor, LDL receptor-related protein 1 (LRP1) and ApoE receptor 2 (ApoER2) [157,158]. Once internalized, ApoE supplies cholesterol (and other lipids) to neurons, which are required for certain neuronal processes, such as synaptogenesis [37], elongation of axons [38,159,160] and synaptic plasticity [160,161]. In addition, it was reported that ApoE affects the functions of certain membrane receptors, channels, and transporters in postsynaptic neurons [162,163,164].

Offering support for the notion that the astrocyte-derived ApoE in the brains plays an important role in normal cognitive functions, earlier studies have shown that the ApoE knockout mice had learning and memory deficits [165,166,167]. This phenotype of defective memory function was confirmed in transgenic mice with selective astrocytic ApoE knockdown [168].

In addition to supplying neurons with astrocyte-derived cholesterol and CEs, another important function of the brain ApoE is to carry out the efflux of excess neuronal cholesterol. Earlier studies in cultured cells showed that ApoE2 can promote significantly more cholesterol efflux from both astrocytes and neurons than ApoE3, and ApoE3 can promote more cholesterol efflux than ApoE4 [169,170]. Differences in lipid- and lipoprotein-binding properties of ApoE isoforms were also observed in the periphery: while ApoE3 associates preferentially with protein-rich HDL particles, ApoE4 associates more effectively with lipid-rich VLDL particles [170,171,172]. These intriguing observations suggest that ApoE3 is more apt to efflux lipids (including cholesterol and CEs) out of neurons, which is akin to HDL’s function in the periphery, whereas ApoE4 appears to have a reduced ability to efflux lipids but a good ability to supply lipids to neurons, which is somewhat similar to the function of VLDL in the periphery.

At the molecular level, the three ApoE isoforms differ at positions 112 and 158 in the *N*-terminal domain, which are cysteine–arginine substitutions, altering the ability to form cysteine–cysteine dimers [148]. While ApoE4 contains no cysteine residues throughout the protein, ApoE3 contains one cysteine residue (cys112) and ApoE2 contains two cysteine residues (cys112 and cys158). ApoE3 can form ApoE–ApoE and ApoE–ApoA-II dimers through its cys112 residue, and similarly, ApoE2 can more readily form respective dimers as it has two cysteine residues; in comparison, ApoE4 cannot form dimers at all [173]. Since dimeric ApoE2 and ApoE3 can elicit higher lipid efflux than their monomeric forms [169], the inability of ApoE4 to form dimers significantly reduces its ability to efflux excess cholesterol out of neurons.

Consistent with the differential ability of the three ApoE isoforms in neuronal cholesterol efflux, in vivo human and animal studies have reported that they also have different levels of lipidation in the CNS [174,175,176]. While ApoE2 and ApoE3 are better lipidated (with ApoE2 best lipidated), ApoE4 is markedly less lapidated in humans [174] and in transgenic mice [176]. Analysis of the CSF collected from middle-aged and older cognitively normal individuals also showed that ApoE4 is less lipidated than ApoE2 and ApoE3 [174]. Selective expression of the human form of ApoE2, ApoE3 or ApoE4 in transgenic mice with viral constructs confirmed that ApoE4 forms less lipidated ApoE particles compared to ApoE2 and ApoE3 particles [175].

ApoE mediates lipid efflux in the CNS jointly with ATP-binding cassette (ABC) proteins, such as ABCA1, ABCG1, ABCG4 and ABCA7 [177,178]. These proteins are membrane proteins that transport lipid molecules into extracellular space, where they bind apolipoproteins such as ApoE, ApoJ and ApoA-I [178]. Like ApoE, the protein levels of ABCA1 and ABCG1 are increased following activation of the nuclear receptor LXR system, either directly [13] or indirectly [179], to promote ApoE-mediated lipid efflux [177].

### 5.2. Mechanistic Explanation: Why Is ApoE4 Pathogenic in LOAD?

*Review of human study results. APOE* gene was first identified in 1993 as a genetic risk factor for sporadic LOAD [180,181]. Later, extensive epidemiological, clinical and pathological studies have established *APOE* gene as the most important genetic risk factor for sporadic LOAD [182,183,184,185]. While ApoE3 is the most common isoform in the general population, ApoE4 occurs in 40–80% percent of all sporadic LOAD patients who possess at least one copy of the *APOE-ε4* allele [10]. People who inherit one copy of the *APOE-ε4* allele is about three times more likely to develop AD, and people who have two copies of the *APOE-ε4* allele (one from the mother and one from the father) are at least eight times more likely to develop AD than those who have two copies of the *APOE-ε3* allele [4,5,186]. Conversely, the ApoE2 variant appears to be protective in LOAD. People with one copy each of the *APOE-ε2* and *APOE-ε3* allele have only one-fourth the risk of developing AD as people with two copies of the *APOE-ε3* allele [186,187,188].

Human studies have shown that the *APOE4* genotype is associated with an earlier onset of LOAD [183]. The PET scan [189] and post-mortem analysis [3,190,191,192] have shown that AD patients with the *APOE4* genotype have an earlier appearance and more amyloid deposits in their brains.

Functional studies have shown that ApoE4 human individuals are unable to efficiently regulate cerebral glucose metabolism and oxygen utilization compared to ApoE4-negative individuals [193]. Similarly, the glucose uptake (based on FDG-PET scan) was lower in ApoE4 individuals in certain regions of the brain, such as the posterior cingulate, parietal, temporal and prefrontal cortex [194]. Post-mortem analysis of brains from young individuals showed that the *APOE4* genotype is associated with reduced levels of brain glucose and lactate transporters and mitochondrial electron transport proteins [195], clearly suggesting a reduced mitochondrial metabolic activity. Behavioral analysis also showed that the *APOE4* genotype is associated with reduced verbal memory [196] in asymptomatic carriers and reduced visual recall and memory retention even in children [197].

*Review of animal study results.* Some of the more convincing evidence on the pathogenic role of ApoE4 came from detailed analyses of the transgenic AD mice. These mice selectively expressed the human *APOE* genes from their endogenous *APOE* promoter [198], with the expected glial expression of human ApoE isoforms [199]. Compared to *APOE3* mice, *APOE4* mice were impaired in spatial learning [200,201,202,203,204,205]. They were also impaired in other memory-related functions [202,203,206]. More pronounced deficits in behavior and brain functions were observed in older *APOE4* mice [203,207], which are consistent with clinical observations.

The *APOE4* mice also had altered neuronal activity. Electrophysiological analysis of amygdala neurons showed a decreased excitatory transmission in the *APOE4* mouse brain [208]. Evoked release of acetylcholine from hippocampal neurons was also decreased in older *APOE4* mice [209]. The hippocampal neurotransmission also had fewer shortwave ripples and reduced slow gamma wave activity in aged *APOE4* mice [210].

Structural analysis showed that the brains of *APOE4* mice have a simpler structure than the brains of *APOE3* mice [208,211,212,213], including less branching or reduced spine densities. Decreased complexity of neurons in *APOE4* brains was observed in the entorhinal cortex [214,215], consistent with the altered functions in that brain region of human LOAD [216]. In older mice, the *APOE* brains appeared to have a lower vascular density, along with white matter damage [217] and smaller hippocampal regions [201].

*Mechanistic explanation for the pathogenic role of ApoE4 in LOAD*. Most previous studies have focused on the role of ApoE in delivering astrocytic cholesterol to neurons. For instance, it was speculated earlier that ApoE isoform-specific effects on learning and memory are partly due to the reduced ability of ApoE4 than ApoE3 in supplying astrocytic cholesterol to neurons and thus a decreased capacity in supporting synaptic functions [218,219]. Recently, an ApoE cascade hypothesis in AD pathogenesis was proposed [220]. Here, a different explanation is tendered. It is hypothesized that the differential ability of the ApoE isoforms in effluxing cholesterol out of neurons plays a more critical role in determining the pathogenesis of sporadic LOAD because a hampered neuronal cholesterol efflux will lead to elevated cellular cholesterol level, which will disrupt mitochondrial structure and function and will result in reduced formation of neuronal ATP and neuroactive metabolic intermediates, along with increased A*β* formation, tauopathy and acetylcholine deficiency. Provided below is a discussion of the available experimental and clinical observations that support the notion that the *APOE4* genotype is a key risk factor for LOAD on the basis of its decreased ability to efflux excess neuronal cholesterol.

***i.*** It is known that ApoE4 has a reduced ability to efflux cholesterol out of neurons in the CNS compared to ApoE3 and ApoE4 [156,169,221,222]. For instance, ApoE3 has 2.5- to 3.9-fold higher ability to efflux cholesterol than ApoE4. It is known that the dimeric ApoE can induce higher lipid efflux than its monomeric form [169]. As ApoE3 (and also ApoE2) can readily form dimeric forms, ApoE4 cannot form dimers at all. The inability of ApoE4 to form dimer is a major cause for its reduced ability to remove excess cholesterol from neurons.

Consistent with the reduced ability of ApoE4 to efflux cholesterol, individuals with the *APOE4* genotype also have smaller ApoE-containing lipid particles plus more lipid-depleted particles in their CSF, whereas those with the *APOE3* genotype have larger lipidated ApoE particles [174,223,224]. Similar observations were also made in the transgenic *APOE* mice [154,176,225]. While the *APOE4* mice were associated with smaller ApoE4 lipoprotein particles plus more lipid-depleted particles in their brains [175,202,226,227], the *APOE2* or *APOE3* mice were associated with larger lipidated ApoE particles [175].

***ii.*** Interestingly, studies have shown that humans with the *APOE4* genotype have lower levels of ApoE in their CSF than those with the *APOE3* genotype [174,223,224]. Similarly, the ApoE4 mice also have the lowest levels of ApoE in extracts from relevant brain regions, such as the frontal cortex and hippocampus, whereas the ApoE2 mice have the highest ApoE levels in these brain regions [176].

**Figure 4:**
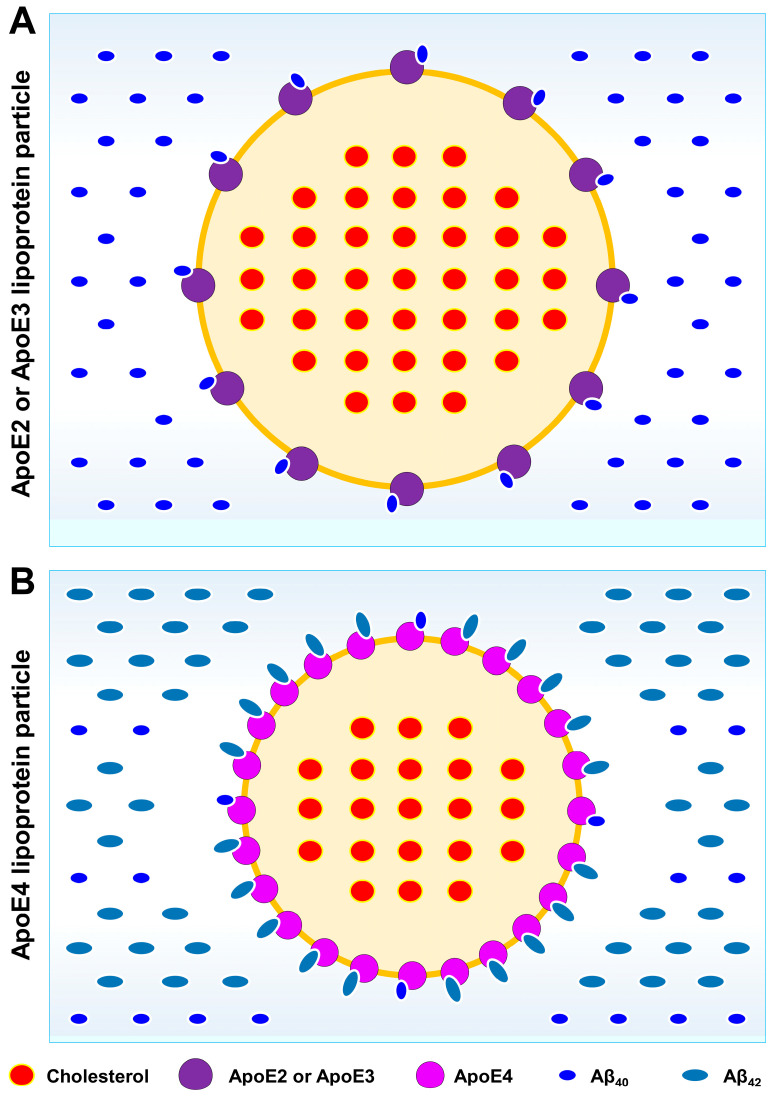
The relative size and density of ApoE2, ApoE3, and ApoE4 molecules in their respective lipoprotein particles. It is known that while the ApoE4 lipoprotein particles have smaller average size than the ApoE2- or ApoE3-containing particles, the ApoE4 particles contain approximately twice the amount of the ApoE4 molecules per particle compared to the E2 or E3 particles. Additionally, ApoE2 and ApoE3 have high binding affinity for A*β*_40_ but very low binding affinity for A*β*_42_ (**A**). In an opposite fashion, ApoE4 has very low affinity for A*β*_40_ binding but high affinity for A*β*_42_ binding (**B**). Because of these unique properties, each ApoE4 lipoprotein particle will bind more A*β*_42_ fragments. It is hypothesized that the higher density of A*β*_42_ bound to each ApoE4 lipoprotein particle will accelerate the deposition of the A*β*_42_-bound ApoE4 particles in the brain (discussed in Section 6), thereby resulting lower ApoE4 levels in the CSF.

To explain why people with the *APOE4* allele have low levels of ApoE4 protein in their CSF, earlier studies suggested that ApoE4 proteins can undergo more rapid degradation than ApoE2 and ApoE3 proteins in the brain [176,228]. The potential reasons for the rapid degradation of ApoE4 particles may include the following: first, despite the smaller size, each ApoE4 particle is known to contain approximately twice the amount of ApoE4 molecules compared to ApoE3-containing particles [229,230] (depicted in Figure 4A,B). As such, the relative density of ApoE4 molecules per lipoprotein particle is actually much higher. In addition, it is known that ApoE3 (and also ApoE2) has very high affinity for A*β*_40_ binding but low affinity for A*β*_42_ binding; in contrast, ApoE4 has very low affinity for A*β*_40_ binding but high affinity for A*β*_42_ binding [180,181,224,231,232]. As a result, while most ApoE3 or ApoE2 particles are bound with A*β*_40_ instead of A*β*_42_, the majority of ApoE4 particles are bound with A*β*_42_ instead of A*β*_40_ (compare Figure 4A with Figure 4B). Due to the higher ApoE4 density per particle, each ApoE4-containing particle will be bound with a lot more A*β*_42_ peptides. It is speculated that the higher density of A*β*_42_ bound to each ApoE4 particle will accelerate the deposition of the cytotoxic A*β*_42_ peptides in the brain (discussed later in Section 6). In the meanwhile, these A*β*_42_-bound ApoE4 lipoprotein particles will stimulate their removal and disposition by different types of cells in the brain, and may even stimulate the cross-BBB transport of these particles, as part of the mechanism that will help remove excess A*β*_42_ peptides (discussed in detail later in Section 9.1). As a result of these accelerated removal processes, people with the *APOE4* genotype actually will end up with lower levels of ApoE4 in their CSF than those with the *APOE3* (or *APOE2*) genotype.

***iii.*** It has been reported that in general, lower levels of ApoE in the brain are often correlated with an elevated risk of LOAD [233]. Based on the discussion provided above, this phenomenon can be quite readily understood. First, in the case of individuals with the *APOE4* genotype, it is already explained above that these individuals will be associated with lower levels of ApoE4 proteins in their CSF, and they are also associated with increased risk for LOAD. Second, if two individuals with the same *APOE3* (or *APOE2*) genotype are compared, it is reasonable to suggest that the one with a lower brain ApoE protein level will have a higher risk for LOAD as this individual will have a reduced capacity of ApoE-mediated cholesterol efflux, thus contributing to elevated neuronal cholesterol (assuming that everything else is the same in these two individuals). Offering partial support for this explanation, an earlier in vivo animal study [234] has shown that administration of bexarotene (an agonist of the RXR nuclear receptor), which can robustly increase ApoE content (likely along with other components of the cholesterol efflux machinery plus cholesterol-metabolizing enzymes) in the hippocampus and cortex of a mouse AD model, was associated with improved memory and cognition and reduced A*β* plaques in these animals. These beneficial effects were not observed in ApoE-deficient mice [234], which is readily understood.

Therefore, based on the mechanistic explanations provided above, it is clear that individuals with the *APOE4* genotype will have overall clinical manifestations highly similar to those with abnormally elevated neuronal cholesterol. With this understanding in mind, a brief discussion of a few other risk-modifying factors in LOAD, including ApoE2, ApoJ, cholesterol-efflux transporters (ABCA1, ABCG1, ABCG4 and ABCA7) and the ApoE receptors is provided below. The role of TREM2 in LOAD is discussed separately in Section 9.1.

### 5.3. Mechanistic Explanation: Why Is ApoE2 Protective in LOAD?

*Review of human study results.* The earlier clinical observations that the *APOE-ε2* allele was under-represented in LOAD patients [235,236] had led to the suggestion that the *APOE2* genotype likely is protective in LOAD patients. Compared to *APOE-ε3/ε3* homozygotes, the risk of LOAD in *APOE-ε2* carriers is ~50% less [188,237]. Moreover, LOAD patients who are *APOE-ε2* carriers exhibit slower cognitive decline compared with non-carriers [238].

Postmortem LOAD brains of *APOE-ε2* carriers had lower densities of amyloid plaques than the brains of *APOE-ε3/ε3* individuals [239,240,241]. PET imaging also showed that the amyloid load in non-demented brains accumulates at a slower pace during aging and has a later onset of amyloid positivity in *APOE-ε2* carriers than in *APOE-ε3/ε3* homozygotes [189]. *APOE-ε2* affects both global amyloid load and region-specific A*β* deposition [242]. In general, the protective effect of *APOE-ε2* is more pronounced in pathologically confirmed LOAD patients than in clinically diagnosed cases [243].

*Mechanistic explanation.* A number of mechanisms have been proposed in the past to explain the protective effect of the *APOE2* genotype. For instance, it was speculated that hyperlipidation of ApoE2 is a central mechanism for the protective effect of ApoE2, and the main reason for this suggestion is that ApoE2 is capable of transporting more astrocytic cholesterol to neurons. In addition, the lipidation state of ApoE2 may affect its binding of A*β* peptides [244,245,246] and thus may affect A*β* catabolism [247]. Additionally, it was previously suggested that the protective effect of ApoE2 may be achieved through ApoE2-activated neuroprotective signaling pathways [248,249,250].

In this article, a new mechanistic explanation is provided. It is hypothesized that individuals with the *APOE2* genotype will have a reduced level of neuronal cholesterol, which will be protective against the development of LOAD. This mechanistic explanation is rather straightforward and is supported by a number of experimental and clinical observations, as briefly elaborated below.

First, people with the *APOE2* genotype have a higher overall capability to efflux neuronal cholesterol in an ABC transporter-dependent manner, and this capability is jointly determined by the following two factors: ***i.*** While ApoE4 cannot form dimers, ApoE2 (like ApoE3) can form dimers [173], which is associated with a higher ability to carry out neuronal cholesterol efflux, in the rank order of ApoE2 > ApoE3 >> ApoE4 [156,169,251]. In addition, studies have shown that the *APOE2* and *APOE3* mice are associated with larger lipoprotein particles, with ApoE2 particles largest and *APOE4* smallest [252,253,254]. ***ii.*** Studies have shown that humans with the *APOE2* genotype have higher levels of ApoE in their CSF than those with the *APOE4* genotype [223,224]. Similar observations were also made in transgenic mice expressing different human ApoE proteins [176].

Second, in addition to the higher ability of ApoE2 to efflux neuronal cholesterol, ApoE2 appears to have a lower ability to deliver cholesterol to neurons compared to ApoE3 and ApoE4, for the following reasons: ***i.*** ApoE2 has far lower binding affinity (approximately 1%) for LDLR (the LDL receptor) than ApoE3 and ApoE4 [230,255,256]. Accordingly, it is speculated that the lipidated ApoE2 particles will have a markedly reduced ability than ApoE3 and ApoE4 to deliver lipids (including cholesterol and CEs) to neurons via LDLR-mediated endocytosis. ***ii.*** LRP1 is another ApoE receptor that mediates the endocytosis of lipidated ApoE particles, and ApoE2 also has a lower binding affinity for LRP1 than ApoE3 and ApoE4 [257]. In fact, the lipidated ApoE4 can bind to LRP1 with a high affinity [258], which further supports the speculation that ApoE4 is highly capable of delivering astrocytic cholesterol to neurons (likely more capable than ApoE3 and ApoE2). ***iii.*** A*β*_40_ has a much higher binding affinity for ApoE2 than for ApoE3 and ApoE4; in comparison, ApoE4 has much higher binding affinity for A*β*_42_ than for A*β*_40_ (depicted in Figure 4) [180,224,232]. As a result, ApoE2 lipoprotein particles will be mostly bound by A*β*_40_, whereas ApoE4 will be mostly bound by A*β*_42_ (Figure 4). As explained in detail later (Section 6.3), when the APP-binding site on ApoE2 is bound by A*β*_40_, ApoE2 is effectively blocked by A*β*_40_ and is thus no longer able to perform its cholesterol-delivering function through endocytosis, but the A*β*_40_-bound ApoE2 lipoprotein can still perform its cholesterol efflux function. As a result, a larger fraction of the ApoE2 particles will be in the cholesterol efflux mode, rather than in the cholesterol-delivery model (i.e., endocytosis mode). This property of ApoE2 also contributes to the reduced ability of the lipidated ApoE2 particles to undergo endocytosis to deliver cholesterol to neurons.

Because of these reasons, it is clear that ApoE2 not only has a higher ability to efflux cholesterol out of neurons, but it may also have a reduced ability to supply astrocytic cholesterol to neurons. A combination of these two properties will beneficially result in reduced brain neuronal cholesterol in individuals with the *APOE2* genotype compared with those with the *APOE3* and *APOE4* genotypes. In agreement with this speculation, it was reported earlier that aged *APOE2* mice had lower cortical cholesterol levels than aged *APOE3* or *APOE4* mice [259].

In light of the above explanation that ApoE2’s neuroprotection mainly results from reduced neuronal cholesterol levels, it is predicted that ApoE2 will be associated with improved mitochondrial metabolic activity (i.e., higher ATP level) and a higher level of neuroactive metabolic intermediates in brain neurons. In addition, the reduced neuronal cholesterol level will also favorably reduce the formation of A*β*_42_ (discussed in Section 6); and the improved neuronal ATP level will help reduce tauopathy (discussed in Section 7) and improve the formation of cholinergic vesicles (discussed in Section 8). Additionally, reduced neuronal cholesterol level will also have a reduced macrophage response in the brain [260,261], which may partly explain why *APOE2* genotype is associated with reduced neuroinflammation―another contributing factor in ApoE2’s protective function in LOAD.

Lastly, it should be noted that ApoE2 not only has a protective effect against LOAD [259,260,261,262,263], but it is also associated with longevity [264,265,266,267,268]. It is hypothesized that the improved mitochondrial function and cellular ATP level in neurons (as well as in other somatic cells) resulting from lower cellular cholesterol level are important underlying factors contributing to longevity. This notion is certainly in line with the long-held view that healthy mitochondrial function is critical for general health and longevity [269,270].

### 5.4. Mechanistic Explanation: Potential Role of APOJ Genotype in LOAD

Besides *APOE*, genome-wide association studies [271,272] have found a statistically significant association between a SNP of the *APOJ* gene and the risk of AD [273]. *APOJ* is not as strong a genetic risk factor as *APOE*; the polymorphic site in *APOJ* has an odds ratio of approximately 0.9 for the *APOJ* allele, compared with an odds ratio of approximately five for the *APOE-ε4* allele [274].

ApoJ is a component of the CNS lipoproteins [275]. It is functionally similar to ApoE and involved in neuronal cholesterol efflux [276] in an ABCA1-dependent manner [277]. It has been suggested that ApoE and ApoJ can interact with lipid debris in the brain and are involved in removing degenerating membranes after neuronal injury [278]. It is hypothesized that the polymorphic ApoJ has a reduced ability to efflux neuronal cholesterol and thus increases the risk for LOAD. Given that ApoJ likely plays a lesser role in CNS cholesterol efflux than ApoE, this may determine why the *APOJ* genotype is not as strong a genetic risk factor as the *APOE4* genotype.

Studies have shown that people with LOAD often have more ApoJ in their blood and its level affects the regional distribution of A*β* [279]. However, the ApoJ level alone cannot be used to predict the onset of LOAD [280]. These observations likely suggest that ApoJ may play a complementary role with ApoE in removing excess neuronal cholesterol. In people with AD conditions, which usually is associated with abnormally elevated neuronal cholesterol (and may also carry the *APOE4* genotype with a reduced overall ability to efflux cholesterol), it is understood that the body may try to increase ApoJ levels as a functional compensation. Understandably, for a LOAD patient with a faster cognitive decline, it likely means that this patient has much higher neuronal cholesterol than usual, and as a result, the body may try to express a lot more ApoJ proteins (along with other brain apolipoprotein isoforms) as a means to help efflux neuronal cholesterol.

Similarly, it was reported that the brain ApoJ level is elevated in proportion to the *APOE-ε4* allele dose level. The explanation for this phenomenon may be related to the fact that the higher *APOE-ε4* allele dose level is associated with lower CSF ApoE4 protein level (already discussed earlier), and that ApoE4 has a very low ability to efflux neuronal cholesterol, and as a result, a stronger induction of ApoJ in the *APOE-ε4* individuals is required to provide a bigger compensation to efflux neuronal cholesterol.

As both ApoE and ApoJ are involved in neuronal cholesterol efflux, they will certainly also jointly contribute to reducing A*β* deposition in the brain. For an individual with the *APOE4* genotype plus a mutant ApoJ, it is expected that this person likely will have a more severe neuronal accumulation of cholesterol and may result in more severe A*β* buildup. The above speculation is supported by earlier studies [281,282]. Additionally, earlier studies have suggested that ApoE and ApoJ may jointly modify A*β* clearance across the BBB (a detailed discussion is provided in Section 9.1).

### 5.5. Mechanistic Explanation: Potential Role of Brain Cholesterol Efflux Transporters in LOAD

The ATP-binding cassette transporters A1 and G1 (i.e., ABCA1 and ABCG1, respectively) are important ApoE-lipidating proteins in the CNS [283,284]. Other members in this transporter family (such as ABCG4 and ABCA7) may also be involved in this process. Earlier biochemical studies have shown that while ABCA1 preferentially activates the efflux of cholesterol and its binding to unlipidated ApoE, ABCG1 is more selective for partially lipidated lipoprotein complexes [285,286]. Studies have suggested that ABCA1 and ABCG1 may work jointly to control the efflux of cholesterol and its oxysterol metabolites [222,287,288].

Since these transporters are all involved in neuronal cholesterol efflux, it is understood that a reduced function of any of these transporters likely will affect neuronal cholesterol level, to varying degrees. As ABCA1 is a well-studied member of this class, a more detailed discussion of its potential role in LOAD will be given here as an example. Alterations in ABCA1 expression were found to be linked to LOAD (reviewed by [228]). Studies of mice with brain-specific deficiency of *ABCA1* manifested mild impairments in neurite morphology, synaptic structures, motor activity and memory formation [288,289,290,291]. As ABCA1 is required for normal acquisition of cholesterol by ApoE, it is expected that the lipidation state of ApoE particles will be reduced in *ABCA1*-knockout mice [283,284].

When *ABCA1* knockout mice are crossed with the mouse model of AD, it is expected that the AD conditions, including amyloid load and the learning and memory deficits, will be more severe as neuronal cholesterol efflux is more severely hampered [292]. On the other hand, overexpression of ABCA1 in these AD mice is expected to help alleviate amyloid deposition and improve brain function, as observed in an earlier study [292].

It is of note that Tangier disease is caused by mutations in the *ABCA1* gene [293]. In the periphery, these mutations prevent ABCA1 from effectively transporting cholesterol and phospholipids out of cells for pickup by ApoA-I in the bloodstream. Tangier disease is characterized by severe plasma deficiency or absence of HDL, apolipoprotein A-I (ApoA-I, the major HDL apolipoprotein) and accumulation of CEs in many tissues throughout the body. Additionally, the buildup of cholesterol in cells can be toxic, causing cell death or impaired functions. These combined factors lead to the peripheral symptoms of Tangier disease. In the CNS, the absence of ABCA1 is associated with poorly lipidated ApoE particles, along with elevated neuronal cholesterol, which impairs neuronal functions.

While it has been suggested that 24*S*-OHC will exit neurons and eventually reach peripheral circulation through simple diffusion (due to its slightly higher water solubility than cholesterol), it was also reported that 24*S*-OHC can be secreted by neurons in an ApoE- and ABCA1-dependent manner [222,287]. As the lipidated ApoE particles can be taken up by astrocytes for metabolic disposition, this process may help deliver 24*S*-OHC to astrocytes where it can exert its important regulatory functions. It is known that in astrocytes, 24*S*-OHC can down-regulate the expression of cholesterol synthesis genes while activate the expression of *APOE*, *ABCA1* and *TREM2* [273]. Mechanistically, the nuclear receptor LXR mediates the action of 24*S*-OHC to regulate the expression of these genes [131,294,295]. An earlier in vivo study has reported that treatment of APP/PS1-transgenic mice with bexarotene (an agonist of RXR) can stimulate the synthesis of ABCA1, ABCG1 and ApoE, which is associated with enhanced clearance of the brain A*β* and reversal of cognitive deficits [234].

As ABCA1 is involved in the efflux of 24*S*-OHC [222,287], it is expected that neuronal secretion of 24*S*-OHC will be reduced when ABCA1 is deficient, and as a result, astrocytic expression of ApoE (a process regulated by 24*S*-OHC-activated LXR) will be drastically reduced. This may explain why ApoE levels in the brain of ABCA1-knockout mice are 80% lower than in wild-type control mice [283,296].

Deletion of the *ABCA1* gene not only decreases the ApoE level [283] but also increases A*β* deposition [297] in the brain; in comparison, *ABCA1* overexpression in the AD mouse model decreases A*β* deposition [292]. These changes are readily understood on the basis of the expected changes in neuronal cholesterol level under these experimental conditions. Deletion of the *ABCA1* gene will be associated with increased neuronal cholesterol level, whereas *ABCA1* overexpression will reduce neuronal cholesterol level. The change in neuronal cholesterol level will then alter the content of CEs in the lipid rafts, which then alters the catalytic activity of *β*-/*γ*-secreatases as well as the formation of A*β*_42_ and A*β*_40_ (detailed explanation is provided in Section 6).

In addition to ABCA1, other ABC transporters (ABCG1, ABCG4 and ABCA7) may also be similarly involved in neuronal cholesterol efflux in the CNS [298,299]. In the light of the functional role of ABCA1 in neuronal cholesterol efflux and LOAD as discussed above, it is expected that abnormalities in the functions of other CNS-resident lipid transporters may also similarly lead to increased neuronal cholesterol levels, along with other accompanying effects. For instance, in vivo studies have shown that mice deficient in ABCG1 exhibit increased neuronal cholesterol level and memory deficits, similar to ABCA1-deficient mice [300,301]. In addition, ABCA7 has also been identified as one of the LOAD susceptibility genes [302], and as expected, studies have shown that deletion of *ABCA7* increases A*β* formation and accumulation in an AD mouse model [303,304].

### 5.6. Mechanistic Explanation: Potential Role of ApoE Receptors in LOAD

When ApoE performs its cholesterol delivery functions in the CNS, it needs to bind to its cell surface receptors, such as LDLR [305], VLDL receptor, LRP1, ApoER2 (also known as LRP8), or heparan sulfate proteoglycans (HSPGs) [64,306,307]. The interactions between ApoE and its receptors have clear isoform preference and are affected by ApoE lipidation status [246,308,309]. For instance, LDLR is only recognized by lipidated ApoE [246,308,309], indicating that LDLR is chiefly involved in the supply of lipids to brain neurons through endocytosis. This suggestion is consistent with the known functions of LDLR in the periphery, which is to deliver lipids to recipient cells. As mentioned in Section 5.3, earlier studies have shown that ApoE2 has a markedly weaker binding affinity for LDLR compared to ApoE3 and ApoE4 [255,256], indicating that lipidated ApoE2 lipoprotein particles have a lower ability to deliver lipids to neurons than ApoE3 and ApoE4.

In addition to LDLR, LRP1 is also known to mediate neuronal endocytosis of lipidated ApoE particles [310,311]. It is speculated that lipidated ApoE2 particles have a lower ability to deliver cholesterol to neurons via LRP1-mediated endocytosis. There is some experimental evidence offering partial support for this suggestion as follows: first, ApoE2 has a lower binding affinity for LRP1 compared to ApoE3 and ApoE4 [257]. Second, A*β*_40_ is known to have a much higher binding affinity (approximately 20-fold higher affinity) for ApoE2 than for ApoE3 and ApoE4 [180,181,224,232], and as such, the APP-binding site on ApoE2 (which is required for endocytosis; discussed later in Section 6) will be effectively blocked by A*β*_40_ and will thus reduce the chances for lipidated ApoE2 particles to undergo endocytosis. This explanation is mostly in line with the known functional profiles of LPR1 in mediating the endocytosis of various ApoE-containing chylomicron remnants in the periphery (reviewed in [157,312]).

At present, much less is known about the receptors involved in neuronal cholesterol efflux. It is speculated that LRP1 may be involved in the efflux of cholesterol, in addition to its ability to mediate endocytosis of ApoE-rich lipoproteins. Similarly, ApoER2 may also be involved in neuronal cholesterol efflux. These are purely speculations, and the experimental evidence for these speculations is mostly lacking at present, except the observations that polymorphisms in LRP1 [251,313,314] and ApoER2 [310] were associated with increased incidence of LOAD.

### 5.7. Section Summary

ApoE lipoproteins in the brain are responsible for carrying astrocyte-derived cholesterol (and other lipids) to neurons, and they are also responsible for effluxing excess neuronal cholesterol. Past studies have mostly focused on the function of ApoE in delivering astrocytic cholesterol to neurons. Here, a new hypothesis is proposed, which suggests that the differential ability of the ApoE isoforms in effluxing neuronal cholesterol contributes critically to the pathogenesis of sporadic LOAD, as a retarded cholesterol efflux will lead to cellular and mitochondrial cholesterol elevation, which will then disrupt mitochondrial structure and function, and reduce the synthesis of ATP and neuroactive metabolic intermediates, along with increased A*β* formation, tauopathy, and cholinergic deficiency.

ApoE4 is known to have a markedly lower ability than ApoE2 and ApoE3 to efflux cholesterol out of neurons, partly due to its inability to form dimers. The reduced ability of ApoE4 to efflux excess neuronal cholesterol will result in markedly elevated neuronal cholesterol and thus elevated LOAD risk. Based on this mechanistic explanation, it is also understood that individuals with the *APOE4* genotype will have overall clinical manifestations highly similar to those with abnormally elevated neuronal cholesterol.

As ApoE4 has a very low affinity for binding A*β*_40_ but a higher affinity for binding A*β*_42_, the ApoE4 lipoprotein particles will be bound mostly with A*β*_42_ instead of A*β*_40_. Additionally, due to the higher density of ApoE4 molecules per particle, each ApoE4-containing lipoprotein particle will be bound with a lot more A*β*_42_ peptides compared with an ApoE2 or ApoE3 particle. The higher density of A*β*_42_ bound to each ApoE4 particle will accelerate the deposition of A*β*_42_ peptides in the brain (discussed later in Section 6.3). By contrast, ApoE2 has a far better ability to promote cholesterol efflux from both astrocytes and neurons than ApoE3 and ApoE4. In addition, ApoE2 has a lower ability to deliver astrocyte-derived cholesterol to neurons than ApoE3 and ApoE4. As a result, the neuronal cholesterol level is expected to be lowest with the *APOE2* genotype, median with the *APOE3* genotype, and highest with the *APOE4* genotype. The low neuronal cholesterol level associated with the *APOE2* genotype is an important mechanism for its protective effect against the development of LOAD.

As the *APOE2* genotype is associated with lower neuronal cholesterol, it is readily understood that ApoE2 will be associated with improved mitochondrial metabolic activity (i.e., higher ATP levels) and higher levels of neuroactive metabolic intermediates in brain neurons. Additionally, lower neuronal cholesterol content will favorably decrease the formation of A*β*_42_ (discussed in Section 6); the improved neuronal ATP levels will help reduce tauopathy (discussed in Section 7) and improve the formation of cholinergic vesicles (discussed in Section 8). Lastly, the new mechanism proposed here also offers a good explanation for the observation that ApoE2 not only reduces the risk of LOAD, but it will also promote longevity.

ApoJ is another member of the CNS lipoproteins and is functionally similar to ApoE in neuronal cholesterol efflux. It is understood that the polymorphic ApoJ with a reduced ability to efflux neuronal cholesterol will be associated with an increased risk of LOAD. Similarly, as the ATP-binding cassette transporters (such as ABCA1 and ABCG1) are involved in neuronal cholesterol efflux, reduced functions of any of these transporters will also affect neuronal cholesterol level to varying degrees, thus affecting the risk of LOAD.

## 6. How Does ApoE4 Accelerate Amyloid Plaque Formation in the Brain?

### 6.1. A Brief Review of APP Structure and Function

In 1987, APP was cloned and found to be linked to the pathogenesis of familial EOAD [315]. APP is a type I transmembrane protein which has a large extracellular domain and a small cytoplasmic domain, resembling a transmembrane receptor for an unidentified ligand. Two proteins are highly homologous to APP, namely, the amyloid precursor-like proteins 1 and 2 (APLP1 and APLP2) [316].

**Figure 5:**
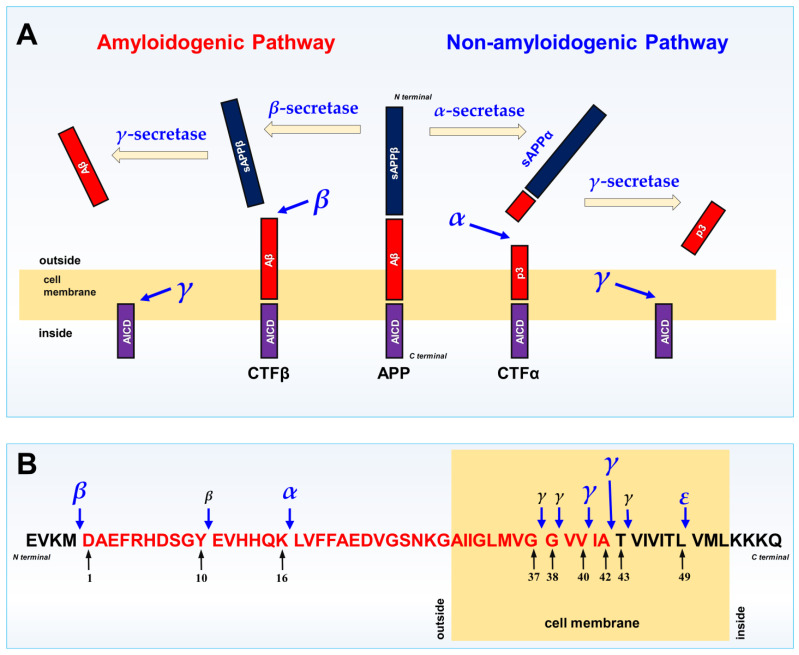
The proteolytic processing of APP. (**A**). The traditional model of APP proteolysis involves APP processing either in the non-amyloidogenic pathway, where sequential cleavage by α-secretase and the *γ*-secretase liberates sAPPα and p3, or in the amyloidogenic pathway, where sequential cleavage by *β*-secretase (BACE1) and *γ*-secretase liberates sAPP*β* and A*β*. Both pathways produce AICD, which can be proteolytically degraded or translocated to the nucleus, where it serves as a transcriptional regulator. (**B**). The exact sites where *α*-, *β*- and *γ*-secretases make the cuts. The major sites for cleavage by *α*-, *β*- and *γ*-secretases are labeled with a big blue-colored *α*, *β* and *γ*, whereas the minor sites for cleavage are labeled with a small black-colored *α*, *β* and *γ*. The amino acid residues constituting the A*β* fragment are labeled in red.

It has been characterized that APP is cleaved by a number of proteolytic enzymes which affect the release of the A*β* peptides (depicted in Figure 5A). The *α*-secretases cleave the extracellular domain of APP’s twelve amino acids away from the membrane and is the functionally most important proteolytic cleavage of APP, which releases the extracellular sAPP*α* domain, along with the membrane-bound *C*-terminal fragment CTF*α*. The *β*-secretase was identified as the *β*-site APP cleaving enzyme (BACE1) [317], which cleaves the extracellular domain of APP farther way from the membrane. While *α*-secretase works primarily at or near the cell surface, *β*-secretase works predominantly in endosomes, consistent with its low pH optimum [317]. The *β*-cleavage of APP results in the release of the soluble ectodomain sAPP*β*, along with the membrane-bound *C*-terminal fragment CTF*β*. The *γ*-secretase, which was identified as a complex of proteins containing the presenilins [318], further cleaves CTF*α* or CTF*β* (following cleavage of APP by *α*- or *β*-secretase).

As depicted in Figure 5A, the sequential cleavage of APP by *α*-secretase followed by *γ*-secretase will generate the following three fragments: sAPP*α*, a small p3 fragment, and the APP intracellular domain (AICD). The *α*-secretase pathway also operates in many non-neuronal cell types, generating shorter fragments that are thought to be nonamyloidogenic. sAPP*α* has been suggested to exhibit neuroprotective and synapse-promoting activities [319]. It was shown that mice that only produce sAPP*α* did not exhibit the various phenotypes caused by a full APP knockout, including disturbed LTP and memory function. This observation suggests that sAPP*α* may mediate some of the functions of the APP holoprotein [320].

The *β*- and *γ*-secretases cleave APP in the so-called amyloidogenic pathway (Figure 5A,B). *β*-secretase releases the ectodomain sAPP*β*, and the remaining CTF*β* is subsequently cleaved by *γ*-secretase liberating the A*β* peptide(s) and the AICD. *C*-Terminal heterogeneity is generated by the *γ*-secretase itself. This protease cleaves APP at different positions, generating a variety of peptides, of which A*β*_43_, A*β*_42_, A*β*_40_, A*β*_38_ and A*β*_37_ variants are detected in cell culture and body fluids (Figure 5B). A*β*_40_ is continuously and abundantly produced in both healthy and AD-afflicted brain tissues, whereas other A*β* peptides are produced at lower levels. A*β*_42_ is the most famous and best studied A*β* peptide and may serve as a core for the formation of amyloid plaques.

The causative mutations in the APP and presenilin genes which alter APP processing were identified many years ago. Moreover, transgenic APP animal models were successfully developed that can produce pathogenic A*β* deposits [321,322].

Interestingly, ApoE receptors also partly share with APP the pattern of proteolysis and undergo surface cleavage to generate soluble (shed) forms of the receptors [323,324]. Several ApoE receptors have been identified as substrates for *β*-/*γ*-secretases, including LRP1 [325,326], and apoER2 [327] and VLDLR [328]. Furthermore, some of the intracellular cytoplasmic adaptor proteins that interact with APP also interact with ApoE receptors [329,330].

### 6.2. Existing Evidence on Neuronal Cholesterol Regulation of Aβ Production

One of the best-known pathological characteristics of AD is the deposition of extracellular A*β* in neuritic plaques [331]. In vitro biochemical analysis has revealed that cholesterol can directly affect A*β* production and deposition [332]. The first evidence for the role of cholesterol in A*β* production in the brain came from an in vivo study demonstrating that dietary cholesterol increased A*β* accumulation in rabbits. Although it is difficult to study the phenomenon in humans, limited human results support the observations made in animal models. For instance, in autopsied AD brains, more severe A*β* deposition was found to be associated with higher levels of blood cholesterol measured earlier during life.

Mechanistically, studies have shown that the cholesterol content in the membrane (particularly in the lipid raft microdomains) is an important factor that regulates the catalytic activity of *β*- and *γ*-secretases [25,26,27,28,29]. This finding provides a mechanistic basis for the causal relationship between elevated neuronal cholesterol and increased A*β* production [26,28,30]. Below is a discussion of how ApoE4 drives A*β* and amyloid plaque formation.

### 6.3. Mechanistic Explanation: How Does ApoE4 Accelerate Amyloid Plaque Formation?

When neuronal cholesterol level is abnormally elevated (likely due to increased supply of astrocytic cholesterol), a number of regulatory mechanisms will be activated to help its removal. First, elevation in neuronal cholesterol will lead to increased metabolic disposition via CYP enzyme-mediated cholesterol oxidation to 24*S*-OHC and other oxysterols, simply due to more substrate molecules becoming available. Since 24*S*-OHC is an activator of LXR in astrocytes, neurons and other brain cells, its increased level will activate a number of regulatory pathways in different cell types, which will jointly help reduce neuronal cholesterol. For instance, 24*S*-OHC can increase the expression of its own metabolizing enzyme CPY46A1, lipid carrier protein ApoE, lipid transporter ABCB1, and others. In addition to 24*S*-OHC, other oxysterols formed in neuronal cells can also serve as LXR activators and will have similar regulatory functions.

**Figure 6:**
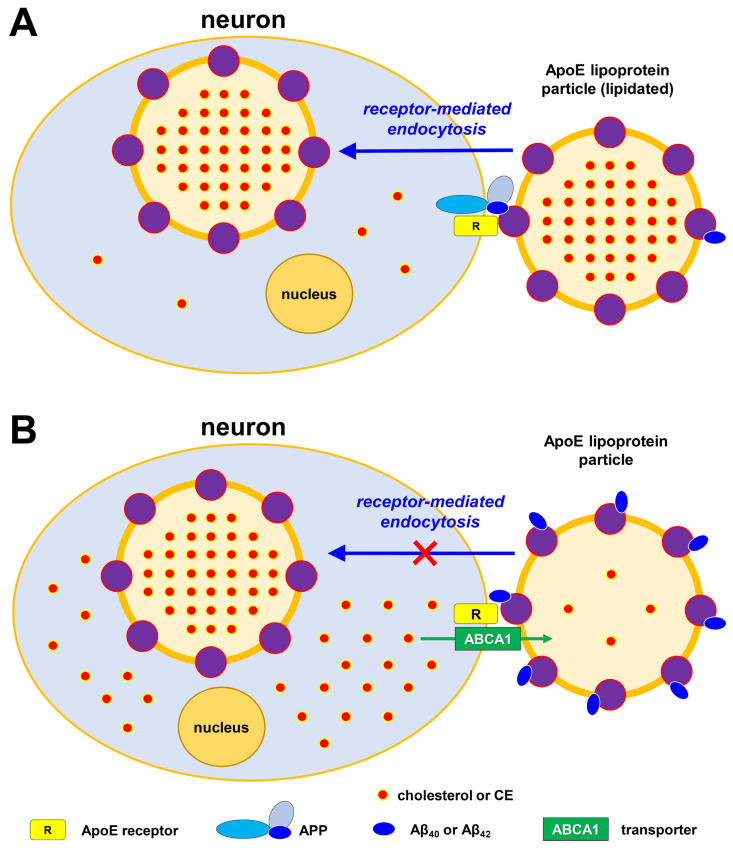
Role of ApoE-containing lipoprotein particles in neuronal transport (i.e., supply and efflux) of cholesterol. As depicted in (**A**), ApoE-mediated delivery of astrocytic cholesterol to neurons is carried out via endocytosis, which involves the binding of lipidated ApoE particles to specific receptors on neuronal surface. It is hypothesized that the intact APP is required for the endocytosis of ApoE particles into neurons, and it serves as a “permissive” signal to the neurons. (Here, it is of note that the cleaved CTFα fragment of APP or the APP-related proteins may also share a similar function as the intact APP in carrying out this “permissive” role.) As depicted in (**B**), when the ApoE molecules in the lipoprotein particle are already bound by A*β* fragments (A*β*_40_ or A*β*_42_), the particle can only bind to its receptor but cannot bind simultaneously to APP (due to competitive blockage). As a result, endocytosis will not be allowed to take place, but under this condition, the ApoE lipoprotein particle can still mediate the efflux of neuronal cholesterol. Therefore, the ApoE particle can be regulated in a precise manner such that it can effectively switch from the function of supplying astrocytic cholesterol to neurons (when neurons are in need of more cholesterol for physiological functions) to the function of removing excess cholesterol from neurons.

Another important mechanism to effectively reduce neuronal cholesterol is to activate its efflux transport via the ApoE lipoprotein particles in an ABCB1-dependent manner. As discussed in Section 5.2 and Section 5.3, it is known that the unlipidated ApoE2 and ApoE3 particles are highly capable of effluxing cholesterol out of neurons, but ApoE4 is markedly less capable in this respect, which is a crucial factor determining the pathogenic activity of ApoE4 in LOAD. Here, I will focus on discussing how changes in factors affecting ApoE-mediated neuronal cholesterol efflux will contribute to A*β* formation and deposition.

First, ApoE-mediated delivery of astrocytic cholesterol to neurons via endocytosis requires the binding of lipidated ApoE particles to their specific receptors present on neuronal surface (here I will use LRP1 as an example for explanation). ApoE is known to bind to APP [333]. It is hypothesized that the intact, uncleaved APP is involved in the endocytosis of a lipidated ApoE particle into a neuron by playing a “permissive” role, which means that only when the lipidated ApoE particle simultaneously binds to its specific receptor (LRP1) and APP will its endocytosis be permitted to take place. Notably, it is speculated that the cleaved CTFα fragment of APP may also share this permissive role. However, when the lipidated ApoE particle only binds to LRP1 but not simultaneously to APP (or CTF*α*), then the endocytosis will not be permitted to take place (as depicted in Figure 6A). In doing so, an ApoE particle can be effectively regulated in a precise manner that it can readily switch from the function of supplying cholesterol to a neuron to the function of removing excess cholesterol from a neuron. It is speculated that the APP-related proteins may share a similar function as APP, as the APP knockout mice do not display severe defects unless the APP-related protein APP1 is also concomitantly knocked out.

When neuronal cholesterol level is relatively low, the APP will be cleaved predominantly by *α*-secretase (which will then form A*β*_17–40_ fragment following further cleavage by *γ*-secretase). However, when elevated cholesterol level is present in a neuron, its APP will undergo enzymatic cleavages by *β*- and *γ*-secretases, resulting in increased production of A*β*_40_ and A*β*_42_ [25,26]. A*β*_40_ and A*β*_42_ peptides are known to bind to ApoE [180,181,232] (as depicted in Figure 5). Moreover, A*β*_40_ can bind effectively to both lipidated and non-lipidated ApoE3 or ApoE2 particles. As depicted in Figure 6B, it is speculated that the binding of A*β*_40_ to these ApoE3 and ApoE2 particles will prevent them from further binding to APP or APP-related proteins present on neuronal surface, thereby preventing these particles from undergoing endocytosis and also preventing the further rise in neuronal cholesterol level. However, the binding of A*β*_40_ to non-lipidated (or less-lipidated) ApoE2 orApoE3 particles will not affect their ability to carry out the cholesterol efflux function, which can help neurons to remove excess intracellular cholesterol. Cholesterol efflux from a neuron relies on the joint function of ABC transporter ABCA1, although other transporters (such as ABCD1 and ABCA7) may also aid in this process, likely to a lesser degree.

LOAD patients with the ApoE4 genotype are known to be associated with more A*β* plaques compared to ApoE2 and ApoE3 carriers. It was suggested earlier that ApoE is involved in A*β* deposition through direct protein–protein interactions, and lipidated ApoE4 binds preferentially to an intermediate, aggregated form of A*β* with higher affinity than lipidated ApoE2 or ApoE3 [246]. Here, a different explanation is provided. It is known that A*β*_40_ has a higher binding affinity for lipidated ApoE2 or ApoE3 particles than for lipidated ApoE4 particles [180,181,224,232]. In addition, ApoE2 dimer can form a complex with A*β*_40_ more efficiently than ApoE3 dimer [232]. By contrast, ApoE4 binds very poorly to A*β*_40_, but binds more tightly to A*β*_42_ [180,181,224,232]. It is known that for each individual ApoE-containing particle, the amount of ApoE4 molecules is about twice the amount of ApoE3 or ApoE2 [229,230]. Therefore, in the case of ApoE4 lipoprotein particles, when the same concentration of A*β*_40_ is present in the SCF, it is expected that there are still a lot of ApoE4 proteins not bound (i.e., not blocked) by A*β*_40_. These unblocked ApoE4 lipoprotein particles can still be endocytosed into neuronal cells, which may cause further rises in neuronal cholesterol level. As such, the neuronal cholesterol level will be higher with ApoE4 than with ApoE3 or ApoE2 (in the order of ApoE4 > ApoE3 > ApoE2). As elevated neuronal cholesterol will increase the expression of APP, this will lead to increased enzymatic cleavage of APP by *β*- and γ-secretases to form more A*β*_40_ within the CE-enriched lipid raft microdomains of the plasma membrane [25,26,28]. Increased formation and release of A*β*_40_ may partially compensate for its lower affinity for binding to the ApoE4 particles. Such regulatory mechanisms through increased expression of APP and increased formation of A*β*_40_ may or may not be sufficient to effectively bring down the elevated neuronal cholesterol level to a range that is physiologically healthy to neurons. However, if such regulatory measures are still inadequate to bring down neuronal cholesterol level to a healthy range, then it will result in further rises in neuronal cholesterol content (including in neuronal plasma membranes). Elevated cholesterol content in lipid raft microdomains will favor the binding of cholesterol to APP, which then increases the formation of A*β*_42_ instead of the usual A*β*_40_ [25,26,28]. In fact, there were both in vitro and in vivo studies showing that neuronal cholesterol accumulation can induce A*β*_42_ formation and accumulation in the brain [25,26,27]. Since A*β*_42_ has a significantly higher binding affinity than A*β*_40_ for lipidated ApoE4 particles [180,181,224,232], increased production of A*β*_42_ is expected to help more effectively block the binding sites on ApoE4 particles and thus prevent lipidated ApoE4 particles from being endocytosed into neurons. Based on the above explanation, it is understood that when ApoE4 is the apolipoprotein, brain neurons will be forced to produce a lot more A*β* peptides, including both A*β*_42_ and A*β*_40_ (particularly A*β*_42_), to help prevent ApoE4 particles from being endocytosed (Figure 4 and Figure 6B).

Here, it is of interest to note that when neuronal cholesterol level is low, A*β*_17–40_ is the major A*β* peptide formed and the amount of neuronal cholesterol exported by ApoE decreases. Based on this observation, it is tentatively speculated that A*β*_17–40_ may be able to bind preferentially to unlipidated ApoE, and the A*β*_17–40_-bound unlipidated ApoE may not be favored to bind to the ApoE receptors to perform the cholesterol efflux function. This is purely a speculation, and it will be of interest to experimentally examine this possibility in the future.

In addition to LRP1, other ApoE receptors, such as LDLR and VLDLR, can also mediate ApoE endocytosis. As these two ApoE receptors likely are not involved in ApoE-mediated cholesterol efflux, it is uncertain whether the endocytosis mediated by these receptors also requires the binding of APP (or similar proteins) to provide a “permissive signal”. If such a permissive signal is not needed, then it is speculated that these ApoE receptors should have highly effective mechanism(s) for their inactivation when neuronal cholesterol has reached an adequate level; only in this way the unwanted further rise in neuronal cholesterol can be prevented in a timely manner. One of the potential mechanisms may be through the secretase (or other proteinase)-mediated cleavage of these membrane-bound ApoE receptors in a manner that their functions can be very sensitively and effectively terminated when adequate amount of cholesterol is already supplied to neurons. There is some indirect evidence in line with speculation—it was shown earlier that the ApoE receptors can be enzymatically cleaved just like APP by secretases [328].

As mentioned above, the ApoE4-containing lipoprotein is selectively bound with a lot of A*β*_42_ peptides; the ApoE4-bound A*β*_42_ peptides are insoluble and may be highly cytotoxic. A number of cell types in the CNS were reported to have the ability to internalize ApoE-bound A*β*, including astrocytes [231], microglia [334,335,336] and neurons [337], albeit with varying capacities. Immunohistological studies of human AD brains using antibodies against various A*β* epitopes have identified the *N*-terminal-truncated fragments of A*β*_40_ and A*β*_42_ inside the lipofuscin-like granules of astrocytes [338,339]. Similarly, in vitro studies using primary astrocytes in culture showed that these cells can internalize and degrade both soluble [340,341] and insoluble A*β* [342]. Interestingly, the ability of astrocytes to engulf and degrade A*β* is compromised in *APOE* knockout astrocytes, or in wild-type astrocytes upon the addition of an antibody against A*β* or ApoE, or an LDLR family antagonist [231]. These results confirm an essential role of ApoE in initiating the receptor-mediated uptake of A*β*-bound ApoE particles by astrocytes. Primary hippocampal neurons were also reported to internalize A*β* in the presence of ApoE [343], but neurons appear to be less efficient in degrading A*β*, resulting in the formation of high molecular weight A*β* aggregates in their endosomal vesicles [344]. Additionally, A*β* can be taken up by smooth muscle cells delineating the arterioles or by endothelial cells that constitute the BBB [345,346,347,348]. It has been suggested that a fraction of the A*β* fragments can be transcytosed and secreted into the bloodstream [349], which may serve as an effective mean of clearance (discussed later in Section 9.1).

### 6.4. Section Summary

One of the best-known pathological characteristics of AD is the deposition of extracellular A*β* in neuritic plaques. The *β*- and *γ*-secretases cleave APP in the amyloidogenic pathway, resulting in the formation of A*β*_42_, which may serve as a core for the formation of amyloid plaques. Increase in neuronal membrane cholesterol content is known to regulate the catalytic activity of *β*- and *γ*-secretases and thus affect A*β* production and deposition.

A*β*_40_ and A*β*_42_ fragments are known to bind to ApoE, including both lipidated and non-lipidated ApoE3 or ApoE2 particles. It is hypothesized that the binding of A*β*_40_ to these ApoE3 and ApoE2 particles will prevent them from further binding to APP or APP-related proteins present on cell surface, thereby blocking these particles from undergoing endocytosis and thus limiting the further rise in neuronal cholesterol level. Notably, the binding of A*β*_40_ to non-lipidated (or less-lipidated) ApoE3 or ApoE2 particles will not prevent them from carrying out the cholesterol efflux function. Notably, A*β*_40_ has a higher binding affinity for lipidated ApoE2 or ApoE3 particles than for lipidated ApoE4 particles. While ApoE4 binds very poorly to A*β*_40_, it can bind more tightly to A*β*_42_. In addition, each individual ApoE4-containing particle contains about twice the amount of ApoE4 molecules compared to that of ApoE3 or ApoE2 particles. Therefore, in the case of ApoE4 lipoprotein particles, when the same level of A*β*_40_ is present in the SCF, there are a lot of ApoE4 proteins still unblocked by A*β*_40_. These unblocked ApoE4 lipoprotein particles can still be endocytosed into neurons, thus causing further rises in neuronal cholesterol. Elevated neuronal cholesterol will lead to increased expression of APP, which then leads to increased enzymatic cleavage of APP by *β*- and γ-secretases to form more A*β*_42_, which has a much higher affinity to bind and thus block ApoE4 than does A*β*_40_. Therefore, A*β*_42_ is formed as a preferred alternative product when ApoE4 is the apolipoprotein, and it is clear that neuronal cholesterol dysregulation in ApoE4 carriers is a key factor that drives increased A*β* formation and deposition.

Lastly, it should be noted that while increased formation of large amounts of A*β* is an important initial cause in familial EOAD, A*β* accumulation and plaque formation may only represent a secondary pathological change in most LOAD cases and is not the dominant force driving disease pathogenesis and progression.

## 7. What Causes Tauopathy in LOAD?

### 7.1. A Brief Review of the Tau Hypothesis and ATP-Dependent Tau Degradation

*Tau hypothesis.* It is generally believed that microtubules in neurons can serve as tracks, guiding the transport of subcellular organelles and macromolecules from the soma of a neuron to the ends of the axon and back. Additionally, microtubules are involved in other important neuronal functions, such as axonal and dendrite outgrowth, and perhaps neurotransmission. Tau, one of the important microtubule-associated proteins (MAPs), affects microtubule stability and dynamics [350]. A salient feature of many neurodegenerative diseases is the formation of pathological tau aggregates, leading to the formation of NFTs, which mostly contain hyperphosphorylated tau [351].

The tau hypothesis postulates that tau protein abnormalities initiate the AD cascade [352]. In this model, pathologically phosphorylated tau binds to normal tau and inhibits its functions [353,354]. Eventually, they form NFTs inside nerve cell bodies. When this occurs, the microtubules disintegrate, destroying the structure of the cell’s cytoskeleton which would collapse the neuron’s transport system. More recently, it was suggested that abnormal tau phosphorylation may subsequently trigger A*β* accumulation in LOAD [355].

While tau is mostly a cytosolic protein, it is also present in the nucleus [356]. Interestingly, the nuclear tau may have a DNA-protective function, which is impaired when tau aggregates [357,358,359]. In line with this suggestion, tau oligomerization is associated with increased DNA damage [360] and cell death [359]; furthermore, expression of a mutant tau is associated with chromosomal instability [361].

Notably, NFTs have been found in other neurodegenerative conditions including Pick’s disease, progressive supranuclear palsy, corticobasal degeneration, and others. The causal relationship between tau dysfunction and neurodegeneration is supported by the identification of over 40 mutations in the tau gene that cause familial frontotemporal dementia with Parkinsonism linked to chromosome 17 (FTDP-17) [362,363].

*Hyperphosphorylation and aggregation.* Tau in tangles is characterized by a high degree of phosphorylation on many residues (45 serines, 35 threonines and five tyrosines) [364]. In AD, tau phosphorylation is increased and the pattern of phosphorylation is altered, as opposed to normal physiological phosphorylation [365]. Phosphorylation of tau is carried out by multiple enzymes [366,367].

Phosphorylation of tau reduces its binding affinity for microtubules, which is associated with reduced microtubule stability and loss of synaptic functions [368]. In addition, hyperphosphorylated tau proteins are prone to aggregation [366], by a process that is not fully understood yet. Aggregated tau proteins would form oligomers, subsequently form fibrils, and eventually assemble into neurofibrillary tangles (NFTs), which are hallmarks of AD [369].

Many factors can induce tau hyperphosphorylation, such as dysregulation of kinase–phosphatase activity and glycation [370]. However, the mechanism underlying the induction of tau hyperphosphorylation is not clear at present.

*ATP-dependent Tau degradation.* The 26S proteasome catalyzes the majority (>80%) of protein degradation in mammalian cells [371], in an ATP-dependent manner. ATP consumption enables substrate unfolding and translocation into an isolated chamber within the proteasome. The rate of hydrolysis of ubiquitinated proteins by the 26S is proportional to its rate of ATP hydrolysis, and the total ATP consumed in degrading ubiquitinated proteins is surprisingly large [372]. In addition, substrate size is also a factor that determines the amount of time and energy needed for proteolysis. The breakdown of large multi-domain proteins will consume greater time and ATP for the 26S to unwind and degrade each successive domain [215].

Notably, protein hyperphosphorylation alters proteasome activity. It has been reported that the proteasome’s capacity to degrade ubiquitinated proteins is defective in neurodegenerative diseases [373,374]. As the proteasome function decreases, ubiquitinated proteins will accumulate in neurons along with phosphorylated tau.

### 7.2. Mechanistic Explanation: How Does Elevated Neuronal Cholesterol Cause Tauopathy?

Neurons require a well-developed cytoskeleton and motor proteins to facilitate the trafficking of various vesicles and organelles; these processes are heavily dependent on cellular energy supply (i.e., ATP). As discussed earlier, elevated neuronal cholesterol will lead to reduced neuronal biosynthesis of ATP. It is hypothesized that when there is a severe shortage of cellular ATP supply, the trafficking of cellular components may not be able to proceed normally. As a result, some of the functions of microtubules may have to be put on hold to save cellular energy for other, perhaps more important, neuronal processes.

As discussed above, tau protein degradation is mediated by the ubiquitin system present in neurons, which requires large amounts of cellular ATP to degrade unwanted proteins. High neuronal cholesterol level will lead to reduced synthesis of ATP, and it is hypothesized that when neuronal ATP is severely deficient, it will lead to a slowdown of ubiquitination-associated degradation of tau proteins. When tau proteins cannot be enzymatically degraded in a timely manner, they will accumulate inside neurons. In order to prevent these tau proteins from performing their normal cellular functions (which will further consume cellular ATP), they are extensively phosphorylated in certain ways, as hyperphosphorylated tau proteins will have reduced functions. However, hyperphosphorylation of tau proteins also facilitates their aggregation. Therefore, based on the proposed pathogenic mechanism, it is speculated that tau accumulation mostly results from the reduced ability of the ubiquitin-proteosome system to effectively degrade tau due to severe deficiency of neuronal ATP, which results from abnormal elevation of neuronal cholesterol levels.

In support of the above mechanistic explanation, an earlier in vitro study has reported that there is a buildup of cholesterol in tangle-bearing neurons, suggesting that increased cellular cholesterol promotes tau hyperphosphorylation [31,375]. Also, it was shown that synaptic accumulation of hyperphosphorylated tau oligomers in AD is associated with dysfunction of the ubiquitin-proteasome system [376].

Here, it is of interest to note that in an A*β* vaccination trial, there were two patients who were almost entirely devoid of A*β* deposition but displayed signs of NFT-associated severe pathology (Braak stage VI) [377]. As expected, the intellectual capacity of these patients was severely affected, and the clinical progression of mental decline was not significantly different from untreated AD patients [377]. This observation is consistent with the hypothesis that A*β* formation and accumulation often is a secondary event accompanying neuronal cholesterol elevation, which then results in deficiency in neuronal ATP and neuroactive metabolic intermediates. In these two patients, although their A*β* deposition is almost completely removed by A*β* vaccines, if their neuronal cholesterol remains at high levels, then their cognitive functions as well as tauopathy will remain very severe, which are determined by the severity of neuronal ATP deficiency and are largely independent of the severity of A*β* deposition.

In the light of the suggestion that NFT formation is the result of severe neuronal ATP deficiency, it is reasonable to suggest that the appearance and spread of NFTs throughout the brain will more closely reflect the degree of ATP deficiency in brain neurons, resulting from abnormally elevated neuronal cholesterol. In other words, the degree of tauopathy is expected to be significantly less severe if neuronal ATP synthesis is not severely inhibited in those AD cases. In agreement with this suggestion, earlier human studies have shown that dementia symptoms and neuronal loss of AD correlate better with the appearance and spread of NTFs throughout the brain than with the deposition of A*β* in senile plaques [378,379].

Based on the explanation provided above, it is further speculated that factors that can inhibit neuronal ATP synthesis likely will also induce tau hyperphosphorylation and subsequently certain degrees of tauopathy. In partial support of this speculation, earlier studies using cultured neuronal cells have shown that oxidative stress and environmental toxins (e.g., arsenite) that are capable of inhibiting mitochondrial metabolic function [380,381] can induce tau hyperphosphorylation [382,383]. Similarly, methanol has also been reported to induce tau hyperphosphorylation and decrease the cognitive ability in animal models [384]. Mechanistically, methanol is known to be metabolically converted to formic acid in the body, which is a strong inhibitor of mitochondrial oxidative phosphorylation [385], thus reducing cellular ATP synthesis. Notably, an earlier study reported that A*β* can increase tau hyperphosphorylation in neuronal cells that take up these secreted peptides [386]. This observation is likely due to the fact that endocytosis of A*β* fragments is usually associated with the endocytosis of A*β*-bound ApoE particles (enriched with cholesterol and other lipids), which will disrupt mitochondrial function and reduce ATP production in neurons.

It is known that the two classic lesions of AD (i.e., A*β* deposits and NFTs) can occur independently in other brain diseases. NFTs found in AD have also been described in many less common neurodegenerative diseases (e.g., frontotemporal dementia, subacute sclerosing panencephalitis and progressive supranuclear palsy) that essentially lack A*β* protein deposits. Conversely, diffuse A*β* deposits can be seen in aged “normal” brains with almost no NFTs. The fact that NFTs composed of aggregated forms of tau proteins occur in certain diseases in the absence of A*β* protein deposition is not surprising as severe ATP deficiency is a main driving force for the formation of NFTs in brain neurons. Besides elevated neuronal cholesterol levels, other factors can also cause neuronal ATP deficiency. However, when neuronal cholesterol is abnormally elevated, increased A*β* production and deposition may be more readily seen, often jointly with the accumulation of NFTs. During this process, the degree of memory and cognitive impairment will largely depend on the degree of deficiency of neuronal ATP and neuroactive metabolic intermediates, both of which are the consequences of elevated neuronal cholesterol. In those aged AD patients that lack significant tauopathy, it is expected that their neuronal cholesterol levels are only modestly elevated, which will slowly but chronically increase A*β* formation and deposition, and eventually may end up with rather heavy buildup of amyloid plaques (if their removal is not as efficient). Because neuronal ATP deficiency in these patients is not as severe, significant tauopathy may not develop as a result; usually these patients are also expected to have slightly better cognitive functions.

In line with the above explanations, earlier studies have noted significant perturbations in the neuronal proteolytic degradation machinery in AD. In fact, a striking morphological change in the AD brains is the accumulation of autophagosomes, autolysosomes, and lysosomal dense bodies in dystrophic neurites [387,388,389]. Similar changes were also observed in APP and/or PS transgenic AD mice [390], even before the onset of amyloid deposition. When autophagy was compromised in the brains of APP-overexpressing AD mice following inactivation of the Beclin-1/*BECN1* gene (*BECN1*^+/−^ mice), enhanced A*β* deposition and neuronal loss were observed, suggesting that decreased autophagy aggravates neurodegenerative progression [391]. However, overexpression of Beclin-1 with the lentiviral vectors attenuated the amyloid load in the injected brain regions.

### 7.3. Section Summary

Elevated neuronal cholesterol not only leads to increased formation of amyloid plaques, but also reduces the synthesis of ATP, and ATP deficiency will then lead to tau hyperphosphorylation and aggregation (i.e., formation of NFTs). Based on this mechanistic explanation, tauopathy will be more readily seen in LOAD as opposed to the familial EOAD. In EOAD, neuronal cholesterol elevation and ATP deficiency are not the initial driving causes, therefore it is speculated that hyperphosphorylation of tau will likely not be as severe initially compared to the situation in sporadic LOAD.

According to the proposed hypothesis, modest reduction in neuronal ATP level is a relatively early event resulting from elevated neuronal cholesterol. As tau accumulation is the result of ATP deficiency, this process will occur relatively early in AD pathogenic process, which is consistent with clinical observations. Notably, a more severe reduction in ATP supply is usually experienced in brain regions that have higher levels of neural activity and thus higher demands for energy supply, such as the medial temporal lobe [392,393]. In fact, this brain region is known to be an early site of tau accumulation (often in the absence of evident amyloid accumulation), and its dysfunction may underlie episodic-memory decline commonly seen in aging and dementia [392,393].

While elevated neuronal cholesterol level increases A*β*_42_ formation, there are also other factors affecting A*β*_42_ formation and deposition. These are the causes why the degree of mental functional decline often is not tightly correlated with A*β* pathology. On the other hand, mental functional decline is more directly associated with degree of neuronal ATP deficiency. Since tau accumulation is the result of neuronal ATP deficiency, it is not surprising that mental functional decline is more closely correlated with tau accumulation. Offering partial support for this suggestion, human [^18^F]flortaucipir PET studies have shown a strong association between regional tau and cognitive decline and neurodegeneration [394].

## 8. What Causes Acetylcholine Deficiency in LOAD?

### 8.1. A Brief Review of the Cholinergic Hypothesis

Acetylcholine is a critical neurotransmitter in the brain involved in memory formation. Moreover, this neurotransmitter is widely used by neurons in the hippocampus and cerebral cortex, regions mostly devastated by AD. In the 1970s, it was found that acetylcholine level falls during normal aging but drops by approximately 90% in people with AD [395,396]. Other neurotransmitters have also been implicated in AD. For example, the levels of serotonin, somatostatin and norepinephrine are also reduced in some AD patients, and deficits in these substances have been suggested to partially contribute to sensory disturbance, aggressive behavior, and neuronal death.

The cholinergic hypothesis is perhaps the oldest hypothesis concerning the potential causes of AD, which postulates that AD is caused by reduced synthesis of acetylcholine. In fact, some of the currently available drug therapies for AD are based on the cholinergic hypothesis [395,396]. Overall, medications that treat acetylcholine deficiency have not been very effective clinically.

### 8.2. Mechanistic Explanation: What Causes Cholinergic Deficiency in LOAD?

The synthesis of acetylcholine neurotransmitter is a single-step reaction catalyzed by the enzyme acetyl-CoA:choline *O*-acetyltransferase (ChAT):

choline + acetyl-CoA ⇌ acetylcholine + CoA

ChAT is found in the nervous system, specifically at sites where acetylcholine synthesis takes place. Within cholinergic neurons, ChAT is concentrated in nerve terminals. In subcellular fractionation studies, ChAT was recovered in the synaptosomal fraction, and within synaptosomes it was primarily in the cytoplasmic fraction. In comparison, acetylcholinesterase (AChE), the enzyme responsible for degradation of acetylcholine, is produced by cells that contain cholinoreceptive sites, although it is also produced by cholinergic neurons.

Under usual conditions, synthesis of acetylcholine is mostly determined by the intracellular availability of choline, which is a limiting step determined by the uptake of choline into the nerve ending. Choline is supplied to neurons either from blood circulation or through metabolism of choline-containing compounds. At least half of the choline used in acetylcholine synthesis comes directly from recycling released acetylcholine, which is hydrolyzed to choline by AChE. Another source of choline is through the breakdown of phosphatidylcholine. Choline derived from these two sources becomes available in the extracellular space and is then subject to high-affinity uptake into the nerve ending. In the CNS, these metabolic sources of choline are particularly important as it cannot penetrate the BBB. Thus, in the CNS, the high-affinity uptake of choline into cholinergic neurons may be saturated, and as such, acetylcholine synthesis can be limited by the supply of choline, at least during sustained neuroelectrical activity. This suggestion is in line with the observation that acetylcholine storage in the brain is subject to variation, whereas acetylcholine storage in other places such as ganglia and muscles remains relatively constant.

However, under conditions of AD, it is hypothesized that acetylcholine deficiency is mainly due to elevations of neuronal cholesterol level, which results in reduced mitochondrial metabolic activity as well as reduced ATP synthesis in neurons and nerve terminals. Since acetyl-CoA is formed as a metabolic intermediate inside the mitochondria, suppression of mitochondrial metabolic activity is expected to result in reduced formation of acetyl-CoA. In addition, since the transport of acetyl-CoA from mitochondria to cytoplasmic compartment is an ATP-dependent process, reduced mitochondrial ATP synthesis resulting from elevated neuronal cholesterol may also reduce the transport of mitochondrial acetyl-CoA to cytoplasmic compartment where acetylcholine synthesis takes place.

In addition to the above two factors, it is important to note that the cholinergic vesicles in nerve terminals not only store acetylcholine, but also ATP and Ca^2+^ [56]. ATP is a required component of cholinergic vesicles, and the release of ATP has been shown to accompany acetylcholine release from these vesicles during neurotransmission [56]. Under conditions of severe ATP shortage, formation of acetylcholine vesicles is expected to drop drastically.

Lastly, the normal process of synaptic neuroelectrical transmission itself also demands high ATP supply [57]. When there is an ATP shortage in cholinergic neurons or nerve terminals, the activity of neurotransmission will be significantly reduced, and the level of decrease likely will depend on the degree of ATP deficiency in these neurons and their nerve terminals.

## 9. The Proposed New Hypothesis Offers a Better Mechanistic Explanation for Relevant Clinical and Experimental Observations

### 9.1. Mechanistic Explanation: Cross-BBB Transport of CNS Lipidated ApoE Particles

It has been generally believed that due to the presence of BBB, cholesterol within the CNS does not readily equilibrate with cholesterol in peripheral circulation [59]. Similarly, it is believed that the peripheral and CNS ApoEs do not cross the BBB and do not exchange their ApoE-containing lipoproteins [397]. Despite these earlier suggestions, it was, however, estimated that a relatively small but significant fraction (up to 0.4%) of the total brain cholesterol pool is “excreted” from the brain every day [59].

To explain the cholesterol turnover in the CNS, it was reported earlier that knockout of *CYP46A1* gene in mice results in over 50% reduction in brain cholesterol excretion and ~40% reduction in brain cholesterol synthesis [122]. Based on this observation, it was suggested that CYP46A1 in mice may be directly responsible for ~40% brain cholesterol turnover [122]. While this explanation is not unreasonable, a different possibility is suggested here. It is speculated that the reduced brain cholesterol turnover in *CYP46A1*-knockdout mice may mostly result from reduced cross-BBB transport of cholesterol-enriched ApoE and ApoJ lipoprotein particles. It is known that the major components involved in this cross-BBB transport, including ApoE, ApoJ, ABCA1 transporter and TREM2, can be up-regulated by 24*S*-OHC, which is formed by CYP46A1. As such, when CYP46A1 is deficient in mice, it not only decreases CYP46A1-mediated metabolic disposition of cholesterol, but it also drastically decreases the cross-BBB transport of cholesterol, due to the absence of the key regulatory factor 24*S*-OHC.

The above explanation is reasonable from a theoretical point of view. It is speculated that neuron-expressed CYP46A1 may mostly serve the function of producing a very small quantity of the signaling molecule 24*S*-OHC, for the purpose of regulating neuronal cholesterol homeostasis. In other words, the actual metabolic capacity of CPY46A1 in brain neurons is likely very limited, as these cells are highly specialized for complex and important neuronal functions. If neuronal CYP46A1-mediated metabolic disposition of cholesterol per se were chiefly responsible for brain cholesterol turnover every day, it probably would be more reasonable that some other major cell types in the brain (such as microglia) play a dominant role in this task. This would be akin to the situation that astrocytes, rather than neurons per se, are largely responsible for the synthesis of lipids (including cholesterol) required to fulfill many neuronal functions of the brain. However, if the brain has the ready means to actively transport excess cholesterol across the BBB into peripheral circulation, then it is readily understood that the selective presence of CYP46A1 in brain neurons (but not in astrocytes) is mostly for the purpose of producing a signaling molecule in a very small quantity for regulating neuronal cholesterol homeostasis.

Here, it is of interest to note that there were earlier human observations that are somewhat in line with the above speculation [398,399,400]. It was observed that after ApoE particles are secreted by astrocytes, they are initially associated with smaller amounts of lipids forming small discoid particles (8–15 nm in diameter), then they increase in size, becoming spherical as they accumulate more lipids, and eventually they are enriched with lipids (12–20 nm with a fraction up to 30 nm) and flow into the CSF [398,399,400]. These observations appear to suggest that there may exist a specific mechanism by which the cholesterol-enriched ApoE particles in the CSF can be transported across the BBB into the peripheral circulation for disposal (note that these ApoE particles are usually richly bound with A*β* peptides to prevent them from endocytosis into neurons). Specifically, it is postulated that the BBB may routinely enable the transport of cholesterol-enriched, A*β*-bound ApoE particles (likely together with ApoJ) into the peripheral circulation in such a fashion that excess neuronal cholesterol and A*β* peptides can be effectively removed. Mechanistically, it is hypothesized that TREM2 present in microglia is involved in the efflux (transcytosis) of A*β*-bound, ApoE-/ApoJ-containing lipid particles across the BBB. Additionally, the LRP1 may also mediate the cross-BBB transport of A*β*-bound ApoE particles. Furthermore, under certain conditions, the BBB may even become selectively “leaky”, enabling some of the peripheral apolipoproteins to gain access into the CNS to aid in the removal of excess neuronal cholesterol. These questions are selectively discussed below, along with a discussion of the supporting evidence.

*Question 1: How does TREM2 contribute to cross-BBB transport of cholesterol?* Animal studies have clearly shown that microglial dysfunction is an important pathogenic factor in LOAD [401,402,403,404]. Microglia are actively involved in phagocytosis-mediated cholesterol clearance in the brain [405], which is perhaps one of the most important functions of microglia. Large-scale human genetic studies have uncovered variants in LOAD risk-associated genes that are highly expressed in microglia [406,407], one of which encodes the triggering receptor expressed on myeloid cells 2 (TREM2), a single-pass transmembrane immune receptor selectively expressed in brain microglia. Individuals carrying rare heterozygous variants of TREM2, such as R47H, have a higher risk for LOAD (average odds ratio of ~4.5) [408,409,410]. Consistent with human study observations, animal studies have clearly shown that TREM2 knockout mice have learning and memory deficits, and transgenic mice that overexpress TREM2 show significant improvements in these functions [411,412]. Additional animal studies have further revealed that the loss-of-function TREM2 variants are associated with increased AD risk [413].

**Figure 7:**
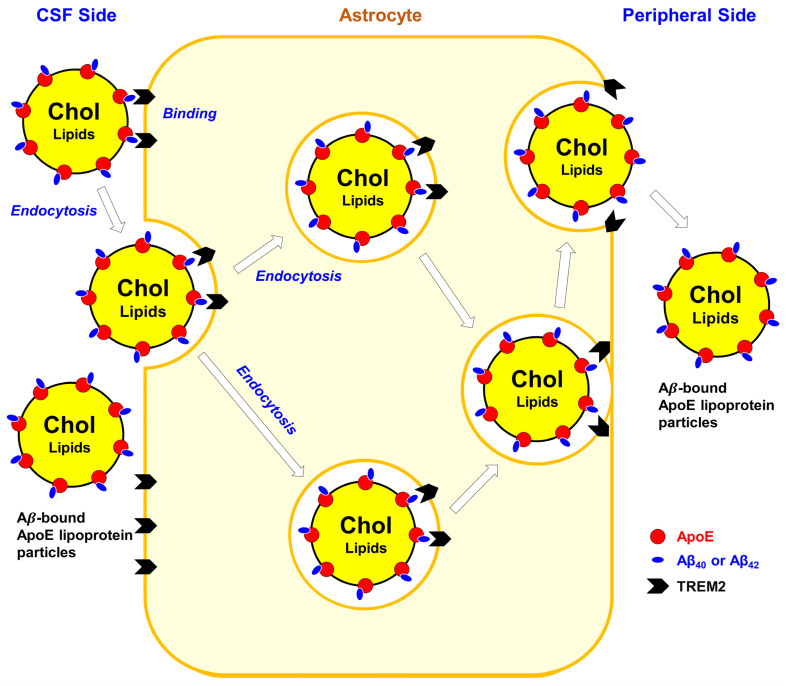
Role of astrocytic TREM2 in mediating the transcytosis of A*β*-bound ApoE lipoprotein particles from the cerebrospinal fluid (CSF) to the peripheral side. As depicted, the extracellular domain (ECD) of the TREM2 protein plays a crucial role in the binding interaction with the A*β* fragments that are tightly bound to the ApoE lipoprotein particles. The binding interaction between these two will initiate the internalization (endocytosis) of the A*β*-bound ApoE lipoprotein particles. In addition, TREM2 plays an even more crucial role in mediating the fusion of the ApoE lipoprotein particle with peripheral side of the cell membrane, i.e., carrying out the release of the particles to the peripheral side. When TREM2 has loss-of-function mutations, the exiting process will be stopped, and it will result in the accumulation of the A*β*-bound ApoE lipoprotein particles inside astrocytes. As these ApoE lipoprotein particles are loaded with cholesterol, it is expected that the astrocytes will have abnormally high levels of cholesterol inside, which will inhibit the ATP synthesis, and will also activate the development of the disease-associated microglia (DAM) phenotype, which is an AD signature phenotype. These changes will also facilitate neuroinflammation. (Note that in the diagram, only ApoE is drawn. It is believed that ApoE and ApoJ may be jointly present in the lipoprotein particles, and the presence of both ApoE and ApoJ may facilitate the process of transcytosis from the CSF side to the peripheral side).

Regarding the protective function of TREM2 in LOAD, it is hypothesized that the observed “microglial clearance” of ApoE and ApoJ [414,415,416] is a process associated with the cross-BBB transport of A*β*-bound ApoE/ApoJ lipoprotein particles. It is speculated that many of these ApoE/ApoJ particles are cholesterol-enriched, although TREM2 is capable of transporting both lipidated and unlipidated particles. A schematic representation of the proposed hypothesis is depicted in Figure 7. It is hypothesized that TREM2 plays a more important role in guiding the phagocytosed vesicle to re-merge with the cell membrane on the peripheral side. When this function of TREM2 is partially or fully lost (such as due to the loss-of-function mutations), the cross-BBB transport of cholesterol-enriched A*β*-bound ApoE/ApoJ particles will be significantly reduced, which will then lead to elevated brain cholesterol level, particularly inside microglia, subsequently increasing the risk of LOAD. As discussed below, there are experimental observations offering partial support for the above mechanistic explanation regarding the role of TREM2 in LOAD.

***i.*** The ectodomain (ECD) of TREM2 is a receptor for binding different lipoproteins (e.g., ApoE, ApoJ, ApoA1, and LDL), although ApoE has been the best-studied ligand [414,417,418]. This binding is independent of the ApoE isoforms [414,417,418] and their lipidation states [417,418]. Earlier studies suggested that ApoJ likely is involved in helping the transport of ApoE-enriched lipoprotein particles across the BBB [414,415,416,417]. It has been reported that AD-associated TREM2 variants (R47H and R62H) indirectly affect the binding of TREM2 with its ligands [410,414,417,418,419,420]. For instance, R47H mutation significantly reduces the binding of TREM2 with ApoE [418] (including all three isoforms [417] with varying lipidation states [414]), other apolipoproteins [414,418], and lipids [421,422]. It is expected that the reduced ability of TREM2 variants for ligand binding will increase the risk of LOAD [414,417,418,422] as these variants will increase cholesterol levels in the brain, especially in microglial cells.

Notably, studies have revealed that the mouse models lacking either ApoE or TREM2 display similar pathologies in amyloid plaques and microgliosis [423,424]. Their similarities are readily understood as both situations will lead to elevated cholesterol in neurons and microglia. Similarly, it is also understood that individuals simultaneously carrying the R47H variant and the *APOE4* genotype would have a markedly elevated risk for developing LOAD [425].

***ii.*** Studies have shown that TREM2 is normally located intracellularly [426] in association with the trans-Golgi network [427,428,429] and in exocytic vesicles [427]. These TREM2-containing vesicles continuously shuttle to the membrane, a process which can be rapidly induced by increases in intracellular Ca^2+^ [427]. TREM2 is recycled from the membrane in clatherin-coated vesicles in a beclin-1 [430] and Vps35-dependent manner [430,431]. Vps35 mediates recycling of TREM2 from the membrane via retromer complexes [431]. In the case of loss-of-function variants [419,432,433,434], TREM2 will have a reduced presence on cell surface but an increased association with lysosomes and is degraded [431].

Consistent with the hypothesis that TREM2 plays a key role in transcytosis of lipoprotein particles, animal studies have shown that microglia lacking TREM2 have a drastic increase in intracellular vesicles [431]. This observation was also made in humans, as microglia in AD patients carrying the loss-of-function TREM2 variants have more intracellular vesicles than microglia in AD patients with the common TREM2 variant. These observations support the notion that TREM2 plays an important role in guiding phagocytosed vesicles to re-merge with the cell membrane on the other side (as depicted in Figure 7). When this function of TREM2 is lost, then it will have a diminished presence on cell surface but an increased association with intracellular vesicles (such as lysosomes) and is quickly degraded [431]. As these vesicles are loaded with cholesterol, it is expected that the microglia with mutant TREM2 will have very high levels of cholesterol inside, which will then inhibit microglial functions and induce neuroinflammation.

***iii.*** It is known that TREM2 serves as a major sensor for extracellular lipids and mediates cholesterol clearance during demyelination. It detects demyelination by sensing the lipidic components of myelin and promotes myelin debris removal via lipid transport and catabolism [435]. Interestingly, it appears that chronic myelin phagocytosis in *TREM2*^−/−^ brain is not affected, but the lack of TREM2 causes accumulation of CEs and oxidized CEs in the *TREM2*^−/−^ brains. These observations agree with the known functions of TREM2, i.e., it plays a more important role in the transcytosis, i.e., the re-merging of the phagocytosed vesicles with the cell membrane on the peripheral side, whereas its role in mediating the initial phagocytic process is less important.

Studies from recent years have identified the disease-associated microglia (DAM) phenotype, which is a transcriptionally distinct microglial profile and closely associated with LOAD pathogenesis [401]. In addition to the expression of the microglial marker gene *TREM2*, this subpopulation of microglia is also defined by the expression of genes involved in lipid metabolism and transport, such as *APOE* [401]. In fact, increased expression of *TREM2* along with other components is widely considered a characteristic shift from the homeostatic phenotype of microglia to the neurodegenerative phenotype [402].

Mechanistically, some earlier studies have suggested that TREM2 may directly control microglial gene expression, resulting in the development of the disease-associated microglia phenotype. While this possibility cannot be ruled out, it is speculated the upregulation of gene expression involved in lipid transport and metabolism is most likely an indirect effect resulting from the functional deficiency of TREM2. It is hypothesized that when neuronal cholesterol is abnormally elevated, the astrocytes will synthesize more ApoE and ApoJ to help efflux cholesterol (and its metabolite 24*S*-OHC). Under conditions when the removal of excess cholesterol is still insufficient (such as due to the presence of ApoE4), more ApoE and ApoJ will be produced. In these situations, there will be a heightened reliance on TREM2-mediated microglial transport of A*β*-bound ApoE/ApoJ particles across the BBB. As such, microglia will be regulated by elevated cellular cholesterol and 24*S*-OHC, further stimulating the expression of TREM2, cholesterol transporters, apolipoproteins (such as ApoE and ApoJ), and metabolizing enzymes (such as SOAT1 and LCAT1), thereby manifesting as the DAM phenotype.

***iv.*** It is known that loss-of-function TREM2 variants can increase the risk of LOAD [423]. These variants under certain conditions (such as chronic demyelination) will lead to accelerated microglial accumulation of intracellular cholesterol in a storage form (i.e., CEs) without altering microglial phagocytic capacity [413]. CEs are known to accumulate in the brains of LOAD patients and AD mouse models [435,436,437,438,439], and the LOAD-linked *TREM2* variants [410] are associated with robust intracellular accumulation of CEs. It is of note that TREM2-deficient and ApoE-deficient microglia show similar abnormalities in cholesterol metabolism, including CE accumulation [423]. Mechanistically, it has been suggested that CE accumulation may mediate cytotoxicity via enzymatic or non-enzymatic formation of oxidized metabolites and lipid peroxides from the mono- and polyunsaturated fatty acid chains the CEs harbor [440,441].

Lastly, it is of note that *TREM2*^−/−^macrophages also exhibit a similar CE storage disorder, resulting in the formation of macrophage foam cells commonly seen in atherosclerotic lesions [442]. Earlier studies of foam cells revealed that cholesterol dyshomeostasis resulting in excess CE accumulation is closely associated with pro-inflammatory responses [440,442,443]. Therefore, it is speculated that abnormal accumulation of cholesterol and CEs in the brain (likely mostly in microglia) is an important factor in neuronal inflammation.

***v.*** Cyclocreatine is a potent bioenergetic agent that helps maintain higher cellular ATP levels during ischemia [444]. Interestingly, dietary administration of cyclocreatine in 5XFAD mice lacking TREM2 prevented the buildup of intracellular vesicles, increased microglia numbers, enhanced clustering around A*β* plaques, and mitigated plaque-associated neurite dystrophy [445]. This observation points to a central role of brain ATP deficiency (presumably caused by elevated neuronal cholesterol resulting from TREM2 deficiency) in microglial dysfunctions and their role in LOAD pathogenesis. This observation also suggests that there are other mechanisms which may compensate for the lack of TREM2 function if adequate microglial ATP supply is maintained.

*Question 2: How does TREM2 contribute to the cross-BBB transport of Aβ?* It is known that microglia can directly internalize and degrade A*β* [446]. Studies have shown that TREM2 can serve as a microglial receptor for A*β*, and the A*β* oligomers can bind to TREM2 with higher affinity than the A*β* monomers [447]. In the AD mouse model, elevated microglial *TREM2* gene dosing decreases the A*β* burden, along with improved memory performance [412]. On the other hand, *TREM2*^−/−^ and TREM2-R47H mutant microglia fail to surround and clear amyloid plaques in vivo, resulting in A*β* plaque buildup and accumulation of dystrophic neurites near plaques [410,420,448,449].

As discussed in the preceding sections, the microglial TREM2 is likely involved in transporting cholesterol-rich ApoE/ApoJ lipoprotein particles across the BBB. It is expected that when these ApoE/ApoJ particles are being transported across the BBB, they would also take along with them considerable amount of A*β* peptides that are tightly bound to these particles. In support of this suggestion, earlier animal studies have shown that ApoJ can bind directly to the soluble form of A*β* in a specific and reversible manner, forming complexes that can cross the BBB [450]. A high-affinity transport system for ApoJ (*K*_d_ of 0.2–0.5 nM) was identified at the BBB and choroid epithelium in vivo, and the ApoJ–A*β*_40_ complex had 2.4- to 10.2-fold higher affinity than ApoJ itself for the same transporter system [282].

*Question 3: How does LRP1/2 contribute to the cross-BBB transport of Aβ and Aβ-bound ApoE or ApoJ particles?* In addition to TREM2, earlier studies have indicated that LRP1 is also involved in the vascular transport of A*β* peptides across the BBB [64]. For instance, it was reported that intracerebrally microinjected ^125^I-A*β*_40_ was quickly removed from the brains of young mice (*t*_1/2_ ≤ 25 min), largely through vascular transport across the BBB. The efflux system for ^125^I-A*β*_40_ at the BBB was half-saturated at 15.3 nM, and the maximal transport capacity reached at 70–100 nM. ^125^I-A*β*_40_ clearance was substantially inhibited by the receptor-associated protein, and by antibodies against LRP1 and α_2_-macroglobulin (α_2_M). There was no evidence that A*β* was degraded into smaller peptide fragments prior to its transport across the BBB. Similarly, another study suggested that BBB-associated pericytes can clear A*β* aggregates via an LRP1/ApoE isoform-specific mechanism [451].

In addition, an earlier study [452] reported that A*β*_40_ binds to immobilized LRP clusters II and IV with high affinity (*K*_d_ = 0.6–1.2 nM) compared to A*β*_42_ and mutant A*β*. Transgenic mice expressing low LRP-clearance mutant A*β* developed robust A*β* cerebral accumulation much earlier than Tg-2576 A*β*-overproducing mice. A*β*_40_ was cleared rapidly across the BBB via LRP1, but A*β*_42_ was removed across the BBB at a slower rate (1.9-fold slower) compared with A*β*_40_.

The transport pathways for clearance of human A*β* and ApoE/ApoJ in the mouse CNS have been reported [453]. ApoE3 is cleared slowly across the BBB, and after lipidation its transport at the BBB becomes barely detectable. ApoJ is eliminated rapidly across the BBB via LRP2. A*β*_40_ binding to apoE3 reduced its cross-BBB efflux by 5.7-fold, but the binding of A*β*_42_ to ApoJ enhanced its cross-BBB clearance by 83%.

*Question 4: What is the potential functional benefit of a leaky BBB phenotype in AD?* An earlier study by Zlokovic and colleagues [454] reported that unlike normal mice, the ApoE knockout mice exhibit a leaky BBB phenotype. Interestingly, selective expression of human ApoE2 or human ApoE3, but not human ApoE4, rescued the leaky BBB phenotype of the mouse. It is speculated that the above experimental observations are likely related to the ability of ApoE2 and ApoE3 to remove excess cholesterol from neuronal cells through the ApoE-mediated cholesterol efflux. Notably, under pathogenic conditions of complete ApoE deficiency, the brains of these transgenic mice could not effectively remove excess cholesterol accumulated inside their CNS neurons. As a result, through certain feedback regulatory processes, the brain of these mice would selectively increase the “permeability” of their BBB such that some of the peripheral apolipoproteins may be allowed into the brain to help remove the excess cholesterol from neuronal cells. While the exact mechanism by which peripheral apolipoproteins are allowed into the brain is not clear at present, studies have shown that under certain conditions, the vascular endothelial cells of the BBB can permit the transcytosis of certain peripheral macromolecules (such as apolipoproteins) to cross the BBB [455]. However, when these ApoE-deficient mice are knocked in to express human ApoE2 or ApoE3, then the functional deficiency in neuronal cholesterol efflux is restored, and understandably, the “leaky BBB phenotype” is no longer needed. However, knock-in of a gene that selectively expresses the human Apo-E4 will not be as helpful as it has a far lower ability to efflux cholesterol out of brain neurons.

There are some experimental observations offering partial support for the above suggestion that the BBB can become selectively leaky under certain conditions. For instance, it is known that cells in the brain do not produce ApoA1, but the cerebrospinal fluid can acquire a significant amount of ApoA1 under certain conditions, most likely from the blood via unknown mechanism(s) [455].

### 9.2. Mechanistic Explanation: Why Is Hypercholesteremia Associated with Increased Risk of LOAD?

Cholesterol present in the CNS is mostly synthesized locally in astrocytes. It is widely held that the two pools of cholesterol (along with ApoE), namely, the one in the periphery and the one in the CNS, are separated, and there is no exchange between these two pools of cholesterol due to the presence of the BBB. However, epidemiological studies have clearly shown that hypercholesteremia is associated with an increased risk of AD [456]. Similarly, in animal studies, it was shown that a cholesterol-rich diet increases A*β* production in the brains of these animals, and an opposite effect was observed in some of the studies when the animals were treated with cholesterol-lowering drugs [457].

The mechanism by which hypercholesterinemia increases the risk of AD is not clearly understood at present. It was speculated earlier that the observed association between hypercholesterinemia and AD likely is because the impermeability of the BBB is compromised in individuals with AD such that cholesterol can be transported into the brain from the plasma [458]. A slightly different explanation is provided here. It is speculated that hypercholesterolemia may largely affect the removal of excess cholesterol from neuronal cells across the BBB into the peripheral circulation. In other words, when the plasma cholesterol level is elevated, it will slow down the net cross-BBB transport of excess cholesterol carried by brain ApoE particles into the peripheral circulation. A reduction in the cross-BBB transport of excess cholesterol will facilitate cholesterol buildup in the CNS (particularly inside neurons) and ultimately result in decreased neuronal synthesis of both ATP and neuroactive metabolic intermediates (mevalonate and geranylgeraniol), contributing to memory and learning deficits. In addition, elevated neuronal cholesterol is expected to facilitate the formation and deposition of A*β* fragments, and the reduced neuronal ATP level will increase tauopathy and decrease the formation of cholinergic vesicles. All these effects will jointly enhance the pathogenesis of AD, particularly LOAD.

### 9.3. Mechanistic Explanation: The Complex Effects of Statins in AD

Statins have been used successfully to treat patients with dyslipidemic cardiovascular diseases [459]. They work by reducing cholesterol synthesis through inhibiting HMGR, a rate-limiting enzyme involved in endogenous sterol synthesis [460]. Most statins can inhibit HMGR with a very high potency (*K*_i_ or *EC*_50_ < 1 nM) [460].

Because hypercholesterolemia is associated with increased incidence of AD (discussed in the preceding sections), the use of statins has been suggested as a potential treatment for AD. Studies using both in vitro and in vivo models have shown that statins can reduce the levels of A*β* peptides, including both A*β*_42_ and A*β*_40_ [457,461]. Based on these observations, many human studies had been initiated to investigate the effects of statins on dementia and cognitive functions. While some human studies have noted a decreased risk of dementia or AD in statin users [462,463,464,465,466,467,468], there were also studies reporting the lack of a clear relationship between the incidence of AD and statin use [469,470,471].

The potential benefits of statin use in AD have been controversial for the following reasons: ***i.*** Some researchers felt that the conclusion regarding a beneficial effect of a peripherally acting statin in AD itself is somewhat “conceptually” controversial, as it has been long held the view that the plasma lipoproteins do not cross the BBB, and that there is no exchange between the central and peripheral pools of cholesterol due to the presence of the BBB. ***ii.*** There are disagreements as to whether it is the peripheral or central lipid-lowering actions of statins that really contribute to the benefits seen in AD. Some researchers strongly felt that if the use of statins is indeed beneficial in AD, then the use of only those statins that can readily cross the BBB will be indicated, rather than the use of peripherally acting statins for this purpose. ***iii.*** The last and most important controversy centers around the question of whether the use of a statin drug is really effective or beneficial in AD, as there are many clinical and animal studies reporting opposite findings. Also, doubts are raised as to whether the potential benefits of statins in AD are really attributable to their cholesterol-lowering effect or might actually be due to some other potential actions of the statin compounds (such as anti-inflammatory and/or antioxidant properties).

As discussed below, the beneficial effects of the statin drugs in AD, and in particular the complexity of their actions in AD, might be more readily understood in the light of the new pathogenic hypothesis proposed in this paper.

First, based on the discussion provided in earlier sections, it is clear that selective inhibition of the peripheral cholesterol synthesis (such as in liver and other peripheral organs) with a statin drug will result in decreased cholesterol levels in circulation, which will be beneficial for the cross-BBB transport of excess neuronal cholesterol to the periphery. Based on this mechanistic understanding, it is clear that the use of a peripherally acting statin will be beneficial for reducing the risk of AD.

Second, when the HMGR in neurons and astrocytes is inhibited by a centrally active statin in addition to their inhibition of the peripheral HMGR, the net effect with respect to AD risk will be more complex [472], and the underlying reasons for the complexity are explained below.

It is clear that the elevated neuronal cholesterol is “bad” for AD, as it will reduce mitochondrial metabolic activity and ATP level in neurons. Additionally, inhibition of the neuronal HMGR by cholesterol will reduce the synthesis of neuroactive metabolic intermediates. In addition, elevated neuronal cholesterol will also increase A*β*_40_ and A*β*_42_ formation and deposition in the brain. These effects jointly contribute to the pathogenic effects of elevated neuronal cholesterol in AD. Based on this understanding, reducing neuronal cholesterol levels through reduced cholesterol uptake (influx), increased cholesterol efflux and/or increased cholesterol metabolism will all be beneficial for restoring altered neuronal functions.

However, reducing neuronal cholesterol level through the use of a centrally active statin drug will not achieve the same beneficial outcomes as it will constantly suppress cholesterol synthesis even when the neurons need it. Worst of all, the much-needed neuroactive metabolic intermediates that are formed along the cholesterol synthesis pathway will always be suppressed by the presence of a centrally active statin drug, which is detrimental to learning and memory formation. These combined effects of the centrally active statins are the underlying causes for the discordant effects on amyloid plaque formation and cognitive functions of the brain. It is expected that during the use of a centrally active statin, while a strong reduction in neuronal cholesterol level will decrease amyloid plaque formation, a decrease in the formation of the neuroactive metabolic intermediates will actually impair cognitive functions.

In line with the above prediction, many randomized, double-blind placebo-controlled clinical studies reported a lack of significant beneficial effect for most centrally active statins on the progression of AD symptoms despite significant decreases in plasma cholesterol levels [472,473,474], while some other studies suggested a potential benefit (such as reduced amyloid load) of the centrally active statins in AD [457,474].

The available results from animal studies have also reflected this complexity. For instance, while some studies showed that simvastatin administration ameliorated learning and memory deficits [475,476,477], opposing observations were also reported [478].

Third, it is of note that when statins were used at super-high concentrations (1000 times higher than the *K*_i_ value), they might have off-target effects that are independent of HMGR inhibition [479]. It has been suggested that neuroprotective effect of statins against AD may also be partly attributable to the anti-inflammatory and/or antioxidant properties of the statins [480,481].

Lastly, it is of note that different from statin drugs, the effect of methyl-*β*-cyclodextrin (which causes acute cholesterol depletion) [482] is expected to be different, since it only helps to reduce cellular cholesterol level without affecting the formation of the neuroactive metabolic intermediates in neurons.

### 9.4. Pathogenic Mechanism of NPC Disease: A Tentative Explanation

The NPC disease is a relatively rare autosomal recessive inherited disorder which causes progressive neurodegeneration and premature death and is often accompanied by hepatosplenomegaly and lung disease [111,483]. The histological feature of the NPC brains includes massive loss of neurons, particularly cerebellum Purkinje cells, which is consistent with impairment of motor function [112]; neurons in other parts of the brain are also affected to varying degrees.

It is speculated that the pathogenesis of NPC disease likely is due to the toxic levels of free cholesterol accumulating in neurons, which will inhibit mitochondrial function and respiration, significantly reduce ATP levels. Therefore, the most pronounced damage is expected to be seen in neurons with a very high density of mitochondria in their cell body. Indeed, studies using cultured neuronal cells and cell type-specific *NPC1* knockout mice have demonstrated that the primary cause of neurodegeneration in the NPC disease is due to NPC1 protein deficiency specifically in neurons, rather than in astrocytes or microglia [484,485]. It is known that the giant Purkinje cells in cerebellum receive an extremely high number of dendritic inputs, making them among the most highly innervated neurons in the mammalian brain. As expected, these neurons also demand a far high level of energy supply and contain an exceptionally high density of mitochondria [486], which are densely packed throughout their enormous, highly branched dendritic trees and proximal axons. Because of these functional and structural features, it is speculated that these large cerebellum neurons are especially vulnerable to the cytotoxicity of abnormal cholesterol accumulation.

At the synaptic level, it has been noted that the morphology and composition of synaptic vesicles are altered by NPC1 deficiency [112]. In addition, the *NPC1*^−/−^ neurons have a reduced exocytosis of synaptic vesicles [487]. It is speculated that the following two factors might be part of the reasons contributing to the observed changes in synaptic functions: one is the reduced cholesterol content in presynaptic membranes, as it is known that NPC1 deficiency will reduce the cholesterol content in the plasma membranes of a neuron, which will then affect the recycling and regeneration of the synaptic vesicles. The other factor is the severely reduced ATP biosynthesis in neuronal cells and particularly in the nerve terminals due to the highly elevated intraneuronal cholesterol levels resulting from NPC1 deficiency. As discussed earlier, neurotransmission and synaptic functions require large amounts of cellular ATP, and when cellular ATP is deficient, vesicular regeneration and release will be severely hampered.

As explained in Section 7.2, the formation of neurofibrillary tangles and tauopathy are closely associated with severe ATP deficiency in neuronal cells. As cholesterol will accumulate in brain neurons of NCP patients, the neuronal ATP levels likely are severely reduced, and as a result, the formation of neurofibrillary tangles and tauopathy will be markedly increased. Indeed, a number of studies have reported the formation of neurofibrillary tangles and tauopathy in many brain regions of the NPC disease [375,488,489]. These observations also agree with the suggestion that neurofibrillary tangles and tauopathy are caused by cholesterol-elicited neuronal ATP deficiency.

Based on the proposed mechanistic explanation, it is understood that amyloid pathology will not be as severe in most cases of NPC disease, which will be in contrast to the severe neurofibrillary tangles and tauopathy seen in this patient. It is known that elevated cholesterol content in the plasma membrane will lead to increased enzymatic formation of A*β*_40_ and A*β*_42_ and amyloid deposition in the brain. However, in NPC1-/2-deficient neurons, it is expected that while the cholesterol level inside the cells is elevated, its level in the plasma membrane will not be similarly elevated. As a result, the enzymatic formation of A*β*_40_ and A*β*_42_ and the subsequent amyloid deposition in the brain likely will also not be similarly elevated. In fact, this explanation fully agrees with the clinical observations.

Although the amyloid pathology may not be severe in NPC patients, the NPC patients still suffer severe deficits in memory and other cognitive functions [490]. Mechanistically, the reduced levels of neuronal ATP and neuroactive metabolic intermediates resulting from high intra-neuronal cholesterol levels in NPC disease are important causes for impaired learning and memory functions. In addition, deficiency in NPC1 results in failed localization of cholesterol in the synaptic membrane, which is involved in synaptic functions, is another contributing mechanism for memory impairments [490].

Additionally, as discussed in Section 9.1, abnormally elevated cholesterol inside brain microglia will lead to microglial activation (i.e., formation of a neurodegenerative phenotype); in addition, ATP deficiency in brain microglia will also trigger inflammatory responses in the CNS. Certainly, these changes will contribute to neurodegeneration and memory impairments in NPC patients. Indeed, studies have shown that like many other neurodegenerative diseases, neuroinflammation is pronounced in the brains of NPC patients as well as in the brains of mouse models of the disease [491].

At present, there is no effective treatment available for NPC disease. Recent experiments, however, have shown a single subcutaneous injection of the cholesterol-binding compound 2-hydroxypropyl-β-cyclodextrin into 7-day-old *NPC1*^−/−^ mice significantly slowed the neurodegeneration and extended the lifespan of the mice by ~50% [492]. Moreover, direct intra-thecal delivery of cyclodextrin into the brain of *NPC1*^−/−^ mice yielded the same beneficial effect on cholesterol as observed in neurons and glial cells isolated from *NPC1*^−/−^ mice [493,494].

Lastly, since the NPC1 and NPC2 proteins are ubiquitously present in all tissues in animals and humans, why only the brain tissue is most severely affected by their deficiency? It is speculated that brain neurons are especially vulnerable to abnormal cholesterol accumulation and its associated damages resulting from NPC1 and NPC2 deficiency; in comparison, the peripheral cells are less vulnerable to the damages caused by cholesterol accumulation as there might be other ways that can more readily help dispose accumulated cholesterol in peripheral cells.

### 9.5. Mechanistic Explanation: Why Is Age One of the Most Important Risk Factors in LOAD?

Age remains the most important risk factor for LOAD [495]. Aging is a complex progressive process involving every organ and cell in the body that can span decades. A number of factors that have been suggested to be potential contributors to brain aging and LOAD, which include glucose hypometabolism and mitochondria dysfunction, innate immune and inflammatory reactions, *β*-amyloid processing, dysregulation of cholesterol homeostasis, white matter degeneration and decline in regenerative capacity.

Based on the newly proposed pathogenic mechanism of LOAD, it is hypothesized that the age-associated hypercholesteremia is an important risk factor in LOAD. In partial support of this hypothesis, human epidemiological studies have reported that elevated levels of total serum cholesterol measured at midlife is associated with increased risk of late-life dementia or cognitive decline [496,497,498,499]. In a population-based human study in eastern Finland, higher cholesterol levels (measured at a mean age of 50) were found to be associated with an increased risk of dementia, mild cognitive impairment and AD during a 21-year follow-up [500]. In another observational study, the risk of late-life dementia is increased by 50% among subjects with midlife hypercholesteremia [501]. A recent human study showed that in twin pairs discordant for dementia, higher cholesterol levels are found in the twins who develop dementia [499]. Most cross-sectional studies showed a correlation between higher HDL levels and lower prevalence of dementia, better cognitive performance, and milder AD pathology [502,503,504]; in comparison, lower levels of serum HDL and Apo-AI are correlated with a more severe AD condition [502].

Lastly, it is speculated that while the need to remove excess cholesterol from the brain may increase with age as neuronal loss increases with age, the actual ability of the brain to remove excess neuronal cholesterol may reduce with age. The combination of these two factors may also contribute to the accumulation of brain cholesterol in the elderly.

### 9.6. Relative Importance of the Amyloid Hypothesis in LOAD Pathogenesis

In 1991, the amyloid hypothesis speculated that the extracellular A*β* deposits are the fundamental cause of AD and believed that these deposits play a crucial role in all cases of AD [6,505]. Specifically, it was postulated that A*β*_42_ may form aggregates which then initiate a pathogenic cascade ultimately resulting in neuronal loss and dementia. Genetic analysis of the rare familial autosomal dominant EOAD has led to the identification of mutations in three genes (i.e., the genes encoding APP, presenilin1 and presenilin2) which are associated with AD and can all increase A*β*_42_ production [4]. Studies have shown that A*β*_42_ is abundantly contained in amyloid plaques of both sporadic and familial AD patients [4,506,507], and it might provide a nidus for amyloid formation [508]. In addition, inheritance of ApoE4 polymorphism was suggested to enhance the stability of A*β* and aid in its accumulation. Based on the similarities in pathology and clinical presentations of familial EOAD and sporadic LOAD, it became widely accepted that A*β*_42_ accumulation also plays a central role in LOAD.

Regarding the mechanism by which A*β*_42_ causes AD, it was suggested, mostly based on in vitro cell culture studies, that A*β* peptides may enter the cells via multiple mechanisms. One mechanism depends on ApoE and the ApoE receptors (especially LDLR and LRP1). The A*β* peptides may bind to ApoE lipoproteins first [509,510], and then endocytosed into neurons and other cell types in the brain. Once inside the cells, the oligomeric A*β* peptides may cause functional disturbances, including alterations in mitochondrial morphology and oxidative stress [511,512,513]; alterations in Golgi morphology and functions [514,515]; alterations in mitochondria-associated membranes [516,517]; alterations in cholesterol metabolism [518]; and alterations in synaptic organelle transport [519]. In addition, A*β* peptides may bind to cholesterol directly [520]. Lastly, there were also studies reporting that at high concentrations, A*β* peptides (in particular A*β*_42_) are cytotoxic to neuronal cells in culture and in the brain in vivo [521,522,523,524].

Based on the above discussion, it is clear that A*β* or A*β* aggregates are cytotoxic to brain neurons when they are present at high concentrations. However, the real contribution of A*β* or A*β* aggregates to the pathogenesis of AD in vivo appears less convincing [525]. As discussed below, there were some clinical and animal studies suggesting that the abundance of amyloid deposition and plaque formation often do not correlate closely with the severity of memory deficits or neuronal toxicity:

***i.*** Human studies have shown that the number of A*β* plaques in the brain does not correlate with the severity of cognitive decrements in patients, and amyloid plaques are sometimes present many years before clinical symptoms are observed. In the process of normal human aging, large amounts of amyloid plaques often are also observed in their brains but with minor neuronal functional alterations, indicating that the relationship between A*β* accumulation and A*β* toxicity is not straightforward [526,527].

***ii.*** In transgenic AD mouse models that overexpress APP and/or presenilin, there lacked a clear relationship between amyloid plaques and cognitive alterations or neurodegenerative changes [528,529].

***iii.*** Animal studies have shown that the transgenic human ApoE4 elicits age-dependent learning and memory impairments in the absence of overt amyloidopathy [200,210,273]. On the other hand, while *APOE-ε2* has a protective effect against A*β* deposition in AD patients, non-demented *APOE-ε2* carriers over 90 years of age (oldest old) have a higher burden of neuritic plaques relative to non-carriers [530,531]. It appeared that *APOE-ε2* carriers are highly resilient to A*β* pathology than non-carriers, and the “oldest old” individuals can survive better from A*β* toxicity and have their cognitive function better preserved.

***iv.*** An earlier study has sought to determine whether A*β* deposition into plaques is the main mechanism by which ApoE isoforms affect AD. The researchers analyzed the murine ApoE-deficient transgenic mice expressing in their brains the human APP and A*β* together with ApoE3 or ApoE4 [532]. It was found that the cognitive decline in AD correlates better with decreases in synaptophysin-immunoreactive presynaptic terminals, choline acetyltransferase (ChAT) activity, and ChAT-positive fibers than with A*β* plaque load [532].

Based on the literature information reviewed above, it is quite clear that there lacks a clear relationship between the severity of A*β* accumulation in the brain and the severity of cognitive alterations in experimental and clinical situations. To better account for the apparent discrepancy between amyloid plaque load and mental functional decline, a modified amyloid hypothesis was later proposed by some researchers, which speculates that the oligomeric forms of A*β* peptides are probably the more toxic molecular species that cause synaptic loss [533,534]. Over 30 years ago, it was demonstrated in vitro that the nontoxic monomeric A*β* could be converted into a more toxic species after incubation for a few days in buffer [535]. The concept of “A*β*-derived diffusible ligands” (ADDL) [521] or “soluble toxic oligomers” [521,522,524] had received a lot of attention because it provided a potential explanation for the toxicity of the extracellular A*β* peptides and particularly for the lack of a correlation between the deposition of the insoluble A*β* plaques and neuronal loss [536]. Several different A*β* oligomeric assemblies have been reported, which were generated in vitro [537], or isolated from transfected CHO cells [538], or from transgenic mouse brains [522]. Different oligomeric species have also been isolated from the brains of AD patient and the smallest toxic isolate was suggested to have a dimeric structure [539]. It was reported that the A*β*_40_ dimers, trimers and tetramers are 3-, 8- and 13-fold more cytotoxic, respectively, than A*β*_40_ monomers. Despite all these earlier studies, no consensus exists as to which toxic A*β* assembly is most relevant in vivo, i.e., which oligomeric *Aβ* form contributes to AD development.

While many of the clinical and animal studies discussed above appear to question the amyloid plaque buildup in the brain as a direct driving force for AD development, there is no denial that A*β*_42_ fragments are involved in the pathogenic process. In fact, it is known that in familial EOAD, the aberrant formation and accumulation of A*β*_42_ are the single cause driving the pathogenic process. Here is the question: how does the increased formation and accumulation of A*β* initiate the pathogenic process of familial EOAD? It is known that in these familial AD cases, there is a marked increase in the formation of A*β*_42_ instead of A*β*_40_ due to mutations in related genes. Using individuals of the homozygous *APOE3* (*ε3/ε3*) genotype as example, it is known that A*β*_40_ has a very high binding affinity for lapidated ApoE3 lipoprotein particles but A*β*_42_ only has a low binding affinity for these particles. As such, it will lead to more ApoE3-containing particles not bound with A*β*_42_ peptides and thus still available for endocytosis by neuronal cells, which will lead to the supply of excess astrocyte-derived cholesterol. Consequently, neuronal cholesterol levels in individuals with familial EOAD will be abnormally elevated, which is believed to be an important causative factor that drives the development of AD symptoms, in addition to further enhancing A*β* formation and plaque formation. Since ApoE2 has a similar binding profile for A*β*_40_ and A*β*_42_ as ApoE3, similar outcomes as described above for the homozygous *APOE3* genotype are expected for individuals carrying the homozygous *APOE2* genotype (*ε2/ε2*) or the *APOE2/E3* mixed genotype (*ε2/ε3*).

However, if a familial EOAD patient happens to have a homozygous *APOE4* genotype (*ε4/ε4*), a different situation is expected. The increased formation of A*β*_42_ in this individual will lead to most ApoE4-containing lipoprotein particles (either lipidated and less lipidated) tightly bound with A*β*_42_. When the ApoE4 particles are bound with A*β*_42_, they cannot be efficiently endocytosed (discussed in Section 6.3). As a result, when the astrocytes need to supply cholesterol to neurons in need, they will have to produce more ApoE-containing particles in order to fulfill this purpose. It is speculated that the excess amount of astrocyte-produced ApoE4 lipoprotein particles will likely be associated with a greater amount of A*β*_42_ (produced by brain neurons) bound to these ApoE4-containing particles. Compared to ApoE3 or ApoE2, ApoE4 is a far more efficient anchor for A*β*_42_ and thus greatly facilitates the formation of A*β*_42_-enriched amyloid plaques in the brain. In addition, the heavy buildup of cholesterol-containing ApoE4 particles (produced by astrocytes) will overburden the brain microglia and vascular cells to transport these A*β*_42_-bound cholesterol-containing lipoprotein particles across the BBB. Depending on the excess amount of A*β*_42_ (and A*β*_40_) being formed, the efflux capacity of the brain will be overwhelmed to varying degrees. When that happens, a greater fraction of the A*β*-bound, cholesterol-enriched ApoE4 lipoprotein particles will be stuck in the CSF, which may cause further neuronal and microglial damage because of the excess cholesterol they carry.

Based on the above explanations, it is understood that in the familial EOAD individuals carrying the “worst genetic background” (i.e., carrying the homozygous *APOE4* genotype + gene mutations for increased A*β*_42_ formation), their neuronal cholesterol levels will actually be among the lowest compared to individuals with all other genetic background. As a result, these individuals may have exceptional I.Q. levels (particularly in terms of their memory function and related cognitive abilities). The underlying cause for this unique phenomenon is likely because the astrocyte-produced ApoE4 (along with the cholesterol it carries) cannot be efficiently delivered into the brain neurons of these individuals, and as a result, their brain neurons will have exceptionally low levels of cholesterol inside and thus higher levels of ATP and neuroactive metabolic intermediates. At present, although there lacks rigorous clinical data to offer a strong endorsement for the above speculation, many clinical practitioners have the impression that there is a fraction of the familial EOAD patients that have exceptional I.Q. levels before their AD diagnosis. Based on the above explanations, only those individuals who carry the so-called “worst combination” of genetic background (i.e., carrying the homozygous *APOE4* genotype + gene mutations for elevated A*β*_42_ formation) may have the exceptional I.Q. levels, but not the other cases.

In summary, if we put aside the assumption that *Aβ* plaque formation is the driving force in AD development, and if we add the component of abnormal neuronal cholesterol in AD pathogenesis, then most of the puzzle pieces will fit together far better, i.e., the mental functional decline is mostly caused by cholesterol-associated deficiency of ATP and neuroactive metabolic intermediates in brain neurons, whereas A*β* accumulation and plaque formation may often only represent a secondary accompanying event, rather than an initial driving force in the pathogenesis of most LOAD cases. As discussed above, even in familial EOAD cases, abnormal cholesterol buildup in brain neurons is still a key pathogenic determinant.

## 10. Potential Strategies for AD Treatment and Prevention

As the number of AD cases keeps rising worldwide, particularly in developed countries, the unmet medical needs for disease-modifying pharmacotherapy continue to grow. At present, there is no cure for AD; the available treatments can only offer relatively small symptomatic benefits but remain palliative in nature [540,541,542]. The commonly prescribed treatments for cognitive problems of AD include: acetylcholinesterase inhibitors (e.g., tacrine, rivastigmine, galantamine and donepezil) and NMDA receptor antagonists (e.g., memantine). These agents offer temporary relief for some of the AD symptoms for a period of time in a subset of patients, but they do not address the underlying pathological process or substantially slow down clinical progression. The overall benefits from their use are relatively small [540,541,542], and presently there is no medication that can clearly delay or halt the progression of the disease.

It is noteworthy that much of the past AD research has centered on the amyloid cascade hypothesis. The identification of *β*- and *γ*-secretases that generate A*β*_42_ spurred a race over the past two to three decades to develop selective chemical inhibitors, antibodies, and vaccines that can effectively target *β*-/*γ*-secretase-mediated A*β*_42_ production. However, based on the new understanding that elevated neuronal cholesterol is the key pathogenic factor in LOAD, these treatments, even if successfully developed and found to be safe, may not be as desirable as initially hoped, since A*β*_42_ may only represent a secondary alteration in the pathogenic cascade in LOAD.

Provided below is a discussion of some of the potential strategies for treating and/or preventing AD in light of the new pathogenic hypothesis developed in this paper.

### 10.1. Centrally Acting Nuclear Receptor Agonists and CYP46A1 Inducers or Activators

*LXR agonists.* ApoE, ABCA1 and related macromolecules work together to help remove excess cholesterol from neuronal cells. These macromolecules are regulated by the nuclear receptor system consisting of LXR and RXR in neuronal cells. As such, CNS-acting LXR/RXR agonists are attractive candidates for AD prevention and treatment [543,544]; these agents can activate the expression of ApoE, ABCA1 and ABCG1 [544,545], resulting in enhanced neuronal cholesterol efflux. Reduction in neuronal cholesterol will not only improve ATP levels but also improve the synthesis of neuroactive metabolic intermediates. Moreover, it is expected that reductions in neuronal cholesterol will help reduce the formation of amyloid/neuritic plaques. Indeed, studies have shown that LXR agonists (such as bexarotene) can effectively increase ApoE and ABCA1 levels in the AD mouse brains, which is coupled with improved synaptic plasticity [545] and behavior [546] as well as reduced A*β* levels [176,234,547]. As expected, these beneficial protective effects are only seen in animals that carry both *APOE* and *ABCA1* genes [548]. Similarly, studies have demonstrated that the RXR agonist bexarotene and its derivative OAB-14 each can effectively rescue impaired ApoE4 lipidation and reverse behavioral deficits in *APOE4* mice [202,549]. Based on these observations, it is quite evident that the brain-penetrable LXR/RXR agonists or modulators might be of therapeutic value for LOAD treatment and/or prevention.

Here, it is of note that while 24*S*-OHC is an endogenous ligand (activator) of the brain LXR and can effectively regulate the expression of ApoE, ABCA1 and other proteins involved in cholesterol efflux, it is ineffective in improving AD symptoms; instead it elicits deficits in learning and memory in a rat model [550]. This observation fully agrees with the proposed hypothesis as 24*S*-OHC is an endogenous cholesterol derivative and can strongly inhibit neuronal HMGR, which will then inhibit the formation of neuroactive metabolic intermediates in neurons and thereby inhibit cognitive function and memory formation.

*CYP46A1 inducers or activators.* CYP46A1 catalyzes the metabolic conversion of cholesterol to 24*S*-OHC [121,124]. Earlier studies have shown that overexpression of *CYP46A1* ameliorates amyloid pathology [128] and tauopathy [551,552] in two different mouse AD models. In addition, overexpression of *CYP46A1* in a mouse model for Huntington’s disease also decreased neuronal atrophy and improved motor neuron deficits [553]. It is hypothesized that the mechanistic basis for the neuroprotective benefits of *CYP46A1* overexpression is due to increased metabolic formation of 24*S*-OH, which then activates LXR and increases the expression of ApoE, ApoJ, ABCB1 and ABCB7, ultimately enhancing the efflux of neuronal cholesterol.

Based on the above discussion, it is reasonable to suggest that selective induction of neuronal CYP46A1 in humans by centrally active inducers may provide therapeutic benefits in AD and other related neurodegenerative diseases. While 24*S*-OHC is an endogenous inducer of CYP46A1 in human brains, this oxysterol is not suitable for this particular purpose as it can also inhibit neuronal HMGR. Theoretically, a centrally active synthetic inducer of CYP46A1 which does not directly inhibit the HMGR would be a useful drug candidate for this particular purpose.

In addition to inducing CYP46A1 expression, studies have shown that the catalytic activity of this neuronal CYP isoform can be activated by some chemicals. Efavirenz, a non-nucleoside inhibitor of reverse transcriptase [553], was reported to activate the enzymatic activity of CYP46A1 for cholesterol metabolism [554,555]. An interesting earlier study in iPSC-derived neurons showed that reducing CE levels through CYP46A1 activation by efavirenz can reduce both *p*-tau and A*β* secretion [556]. Efavirenz has also been evaluated in LOAD patients with mild cognitive impairment [557,558].

### 10.2. Peripherally Acting Cholesterol-Lowering Drugs

In light of the proposed hypothesis, and also according to many earlier clinical and animal studies (discussed in Section 9.3), it is suggested that the use of low-dose, peripherally acting statin drugs, which aims to improve peripheral hypercholesterolemia, will be of some benefit for reducing the risk of LOAD. In addition, it is expected that their use may also be of benefit for slowing down the progression of LOAD or improving the clinical symptoms. By contrast, the use of centrally active statin drugs should be avoided as these agents would constantly inhibit the formation of neuroactive metabolic intermediates which are critically needed for learning and memory formation. Moreover, to avoid the potential central effects of statins, lower doses of the peripherally acting statins be recommended.

### 10.3. Some of the Presently Approved AD Drugs

*Cholinergic replacement therapy.* The discovery that the acetylcholine neurotransmitter is drastically reduced in the AD brain has led to the earlier hypothesis that replacing acetylcholine will be of some benefit for improving AD symptoms. Many researchers have searched for compounds that can increase the levels of acetylcholine, replace it, or slow its breakdown in affected brain regions.

One obvious target is the cholinesterase, which breaks down acetylcholine after its release (discussed in Section 8). Many of the AD drugs developed to date are cholinesterase inhibitors, which are designed to suppress cholinesterase such that acetylcholine will not be degraded as quickly, thereby increasing its levels in the brain to compensate for its shortage [559]. There is considerable evidence for their clinical efficacy in mild to moderate AD cases [560,561], and some evidence for their use in advanced stages. At present, several acetylcholinesterase inhibitors (i.e., tacrine, rivastigmine, galantamine and donepezil) are approved for clinical use for mild to moderate AD, and donepezil is also indicated for advanced AD dementia [562]. The use of these drugs in AD patients with mild cognitive impairment has not shown any efficacy in delaying the progression of the disease [563]. The most common side effects are nausea and vomiting, both of which are linked to cholinergic excess. These side effects arise in 10–20% of users, are mild to moderate in severity, and can usually be managed by slowly adjusting medication doses [564].

*NMDA receptor antagonists.* Glutamate is an excitatory neurotransmitter of the nervous system, although excessive release in the brain can lead to cell death through a process called excitotoxicity resulting from overstimulation of glutamate receptors [565]. Memantine is a noncompetitive NMDA receptor antagonist first used as an anti-influenza agent. It acts on the glutamatergic system by blocking the NMDA receptor and reducing its overstimulation by glutamate [565]. Memantine is moderately efficacious in improving the symptoms of patients with moderate to severe AD [566]. Reported adverse events with memantine are infrequent and relatively mild, including hallucinations, confusion, dizziness, headache and fatigue [567]. The combination of memantine and donepezil was shown to have marginal effectiveness clinically [568].

### 10.4. Candidate Drugs Targeting the Formation and Aggregation of Aβ and Tau

As mentioned earlier, much of the past AD research has focused on the amyloid cascade hypothesis. As a result, developing mechanism-based treatments such as inhibitors of the *β*- and *γ*-secretases and immunotherapeutics (e.g., anti-A*β* monoclonal antibodies and vaccines) has become a major research focus in the past.

*β-Secretase inhibitors.* Studies have shown that knockout of *Bace1* (a gene for *β*-secretase) in mice drastically reduced A*β* production and reduced amyloid plaque load [569,570] and also improved AD-related symptoms in the AD mouse models [571,572]. Therefore, *β*-secretase was viewed as a drug target for AD. Orally effective *β*-secretase inhibitors have been reported earlier [573]. Notably, the relatively mild phenotypes in mice deficient for *β*-secretase compare quite favorably with the severe Notch-related phenotypes caused by broad spectrum *γ*-secretase inhibition (e.g., gastrointestinal bleedings, autoimmune phenotypes).

*γ-Secretase inhibitors.* In the past, several approaches to increase the therapeutic window, i.e., to find compounds that can efficiently block A*β* production yet without affecting the Notch signaling, have been explored. It was estimated that if the *γ*-secretase inhibitors could decrease A*β* production by 30–40%, they would likely not detrimentally interfere with the Notch signaling pathway.

A series of non-transition-state *γ*-secretase inhibitors have been described such as peptide-based inhibitors (DAPT), sulfonamides, and benzodiazepines (compound E). When these inhibitors are used at low concentrations, the *ζ*-site cleavage is not affected, but the *γ*-site cleavages are potently inhibited, which is associated with decreased formation of A*β*_40_ and A*β*_42_ [574].

An unexpected turn in the development of *γ*-secretase inhibitors is the finding that these inhibitors represent a viable therapeutic strategy for certain cancers in which the Notch signaling is overly activated, such as T-cell acute lymphoblastic leukemia [575], lung cancer [576] and precancerous adenoma [577].

*Aβ vaccines.* It was proposed earlier that an alternative potential approach to secretase inhibition is to use small molecules that can bind to A*β* monomers and prevent their aggregation into potentially neurotoxic oligomers. However, from a theoretical point of view, if an anti-aggregating compound solely blocks amyloid fibril formation, this might actually facilitate the accumulation of the intermediates, such as oligomers, and this effect could potentially aggravate neurotoxicity. Alternatively, an immunologic approach to lowering the levels of A*β* protein monomers, oligomers, and higher aggregates was thus proposed. Studies using the APP transgenic mice have shown that parenteral immunization of mice with synthetic human A*β*_42_ initially led to an antibody response associated with striking clearance of A*β* deposits [578]. Subsequent studies have confirmed and extended this approach by showing that A*β* immunization can indeed lower brain A*β* protein burden in mice and may also improve their learning deficits [578].

A number of potential mechanisms have been suggested for the beneficial effects of active immunization [578]. First, the anti-A*β* protein antibodies may cross the BBB in small amounts and bind to A*β* protein, followed by gradual clearing of the resultant A*β* protein-antibody complexes by local microglia. Second, high titers of anti-A*β* protein antibodies in peripheral circulation may bind and sequester A*β* protein in that compartment, resulting in a gradual redistribution of A*β* protein from brain parenchyma to CSF to plasma. Third, the anti-A*β* protein antibodies might bind to soluble A*β* protein oligomers in the brain and neutralize their synaptotoxic effects.

While no untoward antigen–antibody reactions were reported in active vaccination experiments in the mouse models [578], administration of a A*β*_42_ peptide vaccine (with an adjuvant) to humans with mild to moderate AD resulted in approximately 6% of the patients developing an inflammatory reaction in the CNS that resembled a postvaccinal meningoencephalitis [579,580].

Anti-A*β* monoclonal antibodies such as Aducanumab, Lecanemab and Donanemab were developed as a potential therapy for AD [579,580]. They were reported to reduce A*β* levels in animal and human subjects, and their use was associated with modest improvements in cognitive decline in AD patients. However, there are significant side effects associated with monoclonal antibody therapy. The inflammatory responses produced by monoclonal antibodies on brain vasculature are associated with the development of edema and hemorrhage within the parenchyma and sulcal spaces.

Based on the understanding that A*β* accumulation and aggregation may only represent a secondary accompanying change in most cases of AD, the expected benefits of *β*-secretase inhibitors or A*β* vaccines which aim at reducing the overall amyloid plaque load may actually have rather limited clinical benefits in improving the overall cognitive functions in AD patients.

*Tau inhibitors.* Since tau is a major player in AD pathology, research efforts to develop inhibitors of tau aggregation have also been actively pursued [581], in the hope that inhibiting tau aggregation may reduce NFT formation. Several small molecule inhibitors have shown promising results in laboratory conditions but produced mixed results in clinical trials [581]. Tau aggregation inhibitors can be divided into the following two classes: covalent and noncovalent. Covalent inhibitors include polyphenols from plants, such as oleocanthal [582]. Non-covalent inhibitors interact with tau through a different mechanism. Methylene blue, a dye, was found to inhibit tau aggregation, with a *K*_i_ of 120 nM in a cell-based aggregation system [583]. In a study performed in transgenic AD mice, treatment with methylene blue derivatives resulted in a reduction in tau pathology [584]. A phase II clinical trial reported significant improvements after 24 weeks of treatment [585].

Activity-dependent neuroprotective protein is a peptide that is essential for proper brain function. A small segment of this peptide called NAP (NAPVSIPQ) is thought to have a significant neuroprotective ability and is in phase II clinical trials for schizophrenia [586]. In a mouse model of AD, NAP treatment was shown to reduce the level of hyperphosphorylated tau and to improve behavioral symptoms [587]. NAP also protected microtubules against nocodazole-induced disassembly and stimulated the polymerization of microtubules in cultured cells [588]. Since NAP can reduce the hyperphosphorylated tau and protect microtubules against disassembly, NAP is considered a potential therapeutic agent for AD.

Similar to *β-*/*γ*-secretase inhibitors and A*β* vaccines, the expected potential benefits of tau aggregation inhibitors may be limited in AD patients given that tau aggregation is a consequence of severe neuronal ATP deficiency. If the situation of neuronal ATP deficiency (resulting from elevated neuronal cholesterol) persists, even a significant reduction in tau aggregation may not improve learning and memory functions of the AD brain.

### 10.5. Other Potential Candidate Drugs for AD

*ApoA1 mimetic peptides.* Earlier studies have shown that the synthetic ApoA1 mimetic peptides can mimic the effects of ApoE and ApoA1 in stimulating ABCA1-dependent cellular cholesterol efflux (reviewed in [589]). When one of these mimetic peptides, CS-6253, was directly injected into the brains of young ApoE4 mice, it increased the lipidation of the ApoE4-rich lipoproteins [226]. CS-6253 also significantly reversed ApoE4-associated pathology, including A*β* accumulation and tau hyperphosphorylation in hippocampal neurons, as well as synaptic impairments and cognitive deficits. These results suggest that increasing the lipidation of ApoE4-containing lipoprotein particles by using synthetic ApoA1 mimetic peptides may become a potential strategy to combating LOAD in *APOE4* patients.

*SOAT1 Inhibitors.* The CE levels in mouse and human brains under normal conditions are very low, making up <1% of the free unesterified cholesterol. However, in vulnerable brain regions (entorhinal cortex) from AD patients, CE levels are increased by 1.8-fold [437]. In the brains of three different AD mouse models (which express mutant human APP or mutant APP + mutant presenilin 1), the CE levels were elevated 3- to 11-fold compared to the control group [437,590]. In addition, under high-fat diet, the brain CE content in ApoE4 mice was significantly elevated over ApoE3 mice [591]. Together, these results suggest that elevated CE content correlates positively with AD development. In the mouse models for AD, both pharmacological [592,593] and genetic approaches [140,594] showed that inhibition of SOAT1 reduced amyloid plague load and restored cognitive deficits [595].

### 10.6. NSAIDs

Epidemiological studies have repeatedly indicated that the early use of nonsteroidal anti-inflammatory drugs (NSAIDs) is associated with a lower risk of AD in humans [596,597], but they have been unsuccessful in treating AD in clinical trials [598], or preventing AD in short-term prevention trials in the elderly [599]. Interestingly, the preventive effect of NSAIDs appeared to be more pronounced in those with the *APOE4* genotype [597,600], and before the appearance of overt neuropathological changes in LOAD [599]. Similarly, animal studies have also shown that ibuprofen can rescue the effect of *APOE4* genotype on reduced dendritic spine density [214].

The mechanism by which NASIDs protect against LOAD in humans is not understood at present. It has been suggested that some NSAIDs (such as ibuprofen) which have a protective effect against AD may exert its effect through inhibition of the *γ*-secretase-mediated processing of APP to A*β*_42_ [601]. Here, it is speculated that inhibition of the inflammatory responses resulting from cholesterol-induced neuronal ATP deficiency and cellular damage in microglia likely is a major mechanism underlying NASIDs’ neuroprotection in AD.

### 10.7. Nutritional Supplements and Other Natural Neuroprotective Compounds

*Neuroactive nutritional boosters.* Based on the proposed hypothesis, it is apparent that nutritional supplements that can safely supply or boost the synthesis of neuronal ATP and/or neuroactive metabolic intermediates (mevalonate and geranylgeraniol) will be of great value in helping reduce the risk of LOAD. These food supplements may be potentially used as an adjuvant therapy for LOAD. This is a potentially fruitful area of biomedical research that is presently under-explored.

It is of interest to note that dietary administration of cyclocreatine, a chemical capable of increasing cellular ATP level [444], was found to mitigate A*β* plaque-associated neurite dystrophy as well as other injuries in TREM2-deficient mice [445]. Similarly, an earlier study reported that impairments in learning and memory functions in an AD animal model can be restored by administration of geranylgeraniol, a neuroactive metabolic intermediate that can readily across the BBB [50].

*Acetylcholine synthesis enhancers.* The earlier finding of drastic acetylcholine deficits in LOAD also raised hope that dietary supplementation of choline and lecithin may help alleviate the conditions in LOAD patients. These two nutrients are used by the body to synthesize acetylcholine. Clinical trials with these two substances have been disappointing so far: while choline supplements showed no effect on cognitive function, lecithin only had a slight effect in a few patients. Researchers are still searching for other substances that may promote the synthesis and availability of acetylcholine in the brain of LOAD patients.

Studies have shown that acetyl-l-carnitine, a synthetic compound, may activate cholinergic neural transmission and enhance neuronal metabolism in the mitochondria [602] and may thus improve dementia [603] and prevent neuronal degeneration [604]. The exact mechanism by which acetyl-l-carnitine exerts its beneficial biological action is currently unclear [602]. Acetyl-l-carnitine may enhance the synthesis of acetylcholine [603,605] by facilitating the uptake of acetyl-CoA into the mitochondria during fatty acid oxidation [606,607]. Notably, it was reported that plasma l-carnitine levels are inversely associated with cognitive impairment in patients with acute ischemic stroke [608].

*Estrogens.* Gender is an important risk factor in LOAD, as two-thirds of the LOAD patients are women [609]. This proportion is partly attributed to the greater longevity of women over men, making them more susceptible to this and other age-associated diseases [610]. In addition, drastic decline in sex hormone levels in women at older age may be an important factor contributing to increased LOAD risk in elderly women [611].

In 1993, estrogen made headlines when researchers reported a possible link between estrogen and LOAD. In a study of thousands of women in a southern California retirement community, those who had taken estrogen after menopause had a lower incidence of LOAD than those who had not taken estrogen. However, the neuroprotective effect of estrogens has been controversial. Earlier studies that sought connections between estrogen and mental skills showed mixed results [612,613]. While some studies reported a beneficial effect on cognition in women receiving hormone replacement therapy at different ages after menopause [614,615], other studies reported a lack of beneficial effect on reducing LOAD risk [616,617]. Because of the inconsistent epidemiological findings, along with concerns over increased risk for thrombosis, estrogen-based hormone replacement therapy is presently not recommended as a preventive measure for cognitive decline and LOAD [618].

Mechanistically, it was reported that estrogens have neuroprotective effects [619,620] and may prevent mitochondrial dysfunction in neurons [620]. Recent studies showed that 4-hydroxyestrone, an endogenous estrogen metabolite selectively formed in the brain [621], has a stronger neuroprotective effect than its parent hormones 17*β*-estradiol and estrone [621,622]. The protective effects of endogenous estrogens and some of their metabolic derivatives (neuroestrogens) may partially explain their beneficial actions in LOAD. In addition, it is hypothesized that the strong cholesterol-modulating effects of estrogens may also contribute to their overall benefits in LOAD [623]. Here, it is of note that certain endogenous estrogen metabolites (e.g., 4-methoxyestrogens) are devoid of meaningful estrogenic activity in vitro and in vivo but still retain strong cholesterol-lowering activity [623].

*Antioxidants.* The body has lines of defense against reactive oxygen species. Enzymes like superoxide dismutase (SOD) and catalase can disarm the damaging oxygen radicals. Antioxidant vitamins C, E and *β*-carotene are small molecules and can also counter free radicals. The potential protective effects of various antioxidants in LOAD have received considerable interest in the past [624,625], however, their clinical effectiveness has been very limited or controversial. Notably, the lack of a consistent beneficial effect of various antioxidants in human AD appears to be consistent with the recent observations [32] showing that several cytoprotective antioxidants failed to meaningfully rescue cholesterol-induced mitochondrial and cellular toxicities in cultured hippocampal neurons.

*Vitamin D.* Vitamin D can be naturally synthesized in the body, and plays a crucial role in maintaining the health of bones and muscles. In addition, it is reported to prevent certain diseases such as cancer, diabetes, cardiovascular diseases, and autoimmune diseases. Epidemiological studies have suggested a link between low serum vitamin D levels (especially 25-hydroxyvitamin D) and LOAD risk [626,627,628]. Mechanistically, vitamin D is a sterol-derived hormone that regulates calcium metabolism and bone formation, and also plays a role in the brain in regulating neurotrophic factors, oxidative stress, immune system function, and neuroinflammation [629]. In the case of neuroinflammation, it is suggested that vitamin D deficiency activates the amyloidogenic pathway, resulting in elevation of BACE1 and *APP* cleavage and decrease in A*β* degradation [630,631]; vitamin D supplementation in elderly rats reduces BACE1 and A*β* formation. It has also been observed that vitamin D can activate macrophages for clearance of A*β* peptides [632,633]. In AD patients, mutations were observed in vitamin D receptor (VDR) gene, which favors the onset of the disease [634].

In this paper, it is hypothesized that vitamin D may exert its beneficial effects on LOAD by reducing blood cholesterol levels. Decreased blood cholesterol level would help reduce neuronal cholesterol in the brain, which is beneficial to the treatment and prevention of AD. Offering partial support for this hypothesis, there were studies reporting that high blood vitamin D levels are associated with lower LDL levels and higher levels of HDL, but the results from clinical and epidemiological studies are not uniform at present [635]. The mechanism by which vitamin D regulates blood lipid level is presently also unclear.

To date, there is no large randomized clinical trial that investigates the effect of vitamin D supplementation on the cognitive functions in AD patients. However, in smaller or cohort studies, the results on the use of high-dose vitamin D and cognitive improvement are divergent [636,637]. It is recommended that vitamin D deficiency should be screened in the elderly population, and vitamin D supplementation likely will be of greater value in hypercholesterolemia patients who have vitamin D insufficiency and are at elevated risk for both cardiovascular disease and AD.

### 10.8. Role of Physical and Mental Activities

Physical activity is crucial to health regulation since it is significantly linked to obesity, metabolic disease, and atherosclerotic cardiovascular disease [638,639]. The inverse relationship between physical activity and the risk of suffering cognitive decline has been widely documented. Regular physical activity is associated with significant reductions in the risk of developing AD, although there are also discrepancies. Mechanistically, it has been suggested that exercise may cause changes in the brain at the anatomic, cellular, and molecular levels that promote angiogenesis, neurogenesis, synaptogenesis, and stimulation of neurotrophic factors, thereby improving learning, memory, and brain plasticity [640]. It has been reported that exercise increases the gray and white matters of the brain [641], increases cerebral blood flow [642], and reduces A*β* formation and tau phosphorylation [643,644].

Clinical studies have shown that physical activity reduces the risk of LOAD by as much as 45% [645,646]. This protective effect is related to several mechanisms, such as reduction in blood pressure, obesity and proinflammatory activity besides the improvement in lipid profile and endothelial function. In addition, adaptations that occur in response to exercise can lead to a better cerebral blood flow and, consequently, better oxygenation of important areas for cognitive function [645]. It has also been suggested that physical activity can prevent LOAD by increasing neurotrophic factors such as BDNF (brain-derived neurotrophic factor), IGF-1 (insulin-like growth factor) and VEGF (vascular endothelial growth factor), stimulating neurogenesis and synaptic plasticity, and lowering free radicals in the hippocampus and increasing superoxide dismutase and eNOS [647]. Studies have shown that physical activities increase hippocampal volume, in addition to increasing plasma BDNF levels in healthy elderly, indicating a possible neuroprotective effect. It was also reported that in the LOAD elderly, physical activity correlates positively with levels of BDNF [648], a growth factor associated with neuronal survival [649]. Recently, it was reported that the liver-derived exercise factor (exerkine) may reverse aging- and AD-related memory loss by targeting the brain vasculature [650].

It is of interest to note that earlier studies reported that physical activity is associated with highly elevated release of norepinephrine and dopamine in the CNS [651]. Recent studies have revealed that these two catecholamines have a strong protective effect against oxidative ferroptosis in cultured hippocampal neurons [652,653,654]. Mechanistically, norepinephrine and dopamine are capable of covalently binding to protein disulfide isomerase to inhibit its catalytic activity, thus abrogating GSH depletion-associated oxidative neuronal death [652].

Lastly, physical activity may also exert its beneficial effect on LOAD partly through reducing blood cholesterol levels [655,656]. A decrease in total blood cholesterol levels would help reduce neuronal cholesterol in the brain, which is beneficial to the prevention and treatment of AD. This may be another reason for the beneficial effects of physical activity in AD.

### 10.9. Section Summary

Presently, there is still no cure for AD. The available treatments (e.g., cholinesterase inhibitors, NMDA receptor antagonists, and anti-A*β* antibodies) offer relatively small symptomatic benefits but remain palliative in nature. These agents temporarily relieve some of the AD symptoms in a subset of patients. There is no medication at present that can delay or halt the progression of the disease.

Based on the understanding that A*β* accumulation and aggregation may only represent a secondary change in AD, it is expected that *β*-/*γ*-secretase inhibitors or A*β* vaccines may have very limited clinical benefits in improving the overall cognitive functions of AD patients. Similarly, the expected potential benefits of tau aggregation inhibitors may also be limited in AD given that tau aggregation is a direct consequence of neuronal ATP deficiency.

On the other hand, strategies that aim at reducing neuronal cholesterol levels may be highly beneficial in AD prevention and treatment. For instance, agonists for nuclear receptor LXR may activate the expression of ApoE, ABCA1 and ABCG1, resulting in enhanced neuronal cholesterol efflux. CYP46A1 inducers may also have a similar beneficial effect.

It is suggested that the use of low-dose, peripherally acting statin drugs to improve hypercholesterolemia will be of benefit for reducing the risk for LOAD. Their use may also be of benefit to slow down the progression of LOAD or to improve its clinical symptoms. In this context, it is of note that vitamin D may also exert its beneficial effects on LOAD through reducing blood cholesterol levels.

Lastly, based on the proposed hypothesis, it is apparent that dietary supplements that can safely supply or booster the synthesis of neuronal ATP and/or neuroactive metabolic intermediates (such as mevalonate and geranylgeraniol) will be of great potential in reducing the risk of LOAD. These food supplements may even be used as an adjuvant in LOAD therapy.

## 11. Competing Mechanisms and Limitations

### 11.1. Competing and Complementary Mechanisms

While the cholesterol-centered hypothesis provides a scientifically sound pathogenic mechanism for AD (mostly LOAD), there are also competing or complementary pathways and mechanisms, such as impaired brain glucose metabolism, mitochondrial dysfunction, and neuroinflammation. It would be helpful to briefly discuss their pathogenic roles in AD and particularly their relations to the cholesterol-centered hypothesis on AD pathogenesis.

*Impaired brain glucose metabolism*. Studies have shown that impaired brain glucose metabolism is an early, fundamental contributor to LOAD progression, often emerging before clinical cognitive decline and amyloid–tau pathology [657]. As neurons heavily depend on glucose for ATP synthesis via glycolysis and mitochondrial oxidative phosphorylation, reduced glucose uptake and utilization will directly starve high energy-demanding brain regions such as the hippocampus and cerebral cortex. As abnormally elevated neuronal cholesterol can disrupt mitochondrial structure and function, it is believed that the impaired neuronal glucose metabolism as commonly observed in LOAD may, in most cases, only represent a complementary mechanism to cholesterol-centered neurotoxicity.

Here, it is also of note that brain insulin normally supports A*β* clearance, tau dephosphorylation, synaptic maintenance, and memory function. Under conditions of insulin resistance in the brain, it would disrupt insulin-mediated glucose uptake and signaling. As a result, A*β* clearance will decline, along with accelerated plaque formation, while tau hyperphosphorylation will increase, resulting in the formation of NFTs. In addition, dysfunctional glucose metabolism may amplify neuroinflammation by activating microglia and pro-inflammatory pathways, causing further damage to neurons. Therefore, impaired neuronal glucose metabolism under conditions of insulin resistance may represent an independent mechanism further contributing to LOAD pathogenesis.

*Mitochondrial dysfunction.* Mitochondrial dysfunction acts as both an early trigger and an amplifying factor of neurodegeneration [658,659,660]. First, impaired mitochondrial oxidative phosphorylation reduces ATP production, starving high energy-demanding neurons and synapses. This energy deficit weakens synaptic function, impairs signal transmission, and gradually erodes learning and memory functions. Damaged mitochondria may overproduce ROS, causing neuronal oxidative stress. Second, A*β* may inhibit mitochondrial function, including opening of the mitochondrial permeability transition pore and disruption of mitochondrial respiratory chain [54,55,659,660]. Additionally, hyperphosphorylated tau may disrupt axonal transport of mitochondria, trapping damaged organelles at synapses and preventing healthy mitochondrial replenishment. Lastly, in the absence of ATP, mitophagy will not be able to clear dysfunctional mitochondria, allowing toxic organelles to persist. These changes may promote the release of pro-inflammatory signals, which activate glial cells and neuroinflammation and accelerate neuronal loss.

While mitochondrial dysfunction has been widely recognized as a key pathogenic factor in LOAD [659,660], our recent study has demonstrated that elevated neuronal cholesterol can directly disrupt mitochondrial structure and function [32]. In light of this new finding, it appears that mitochondrial dysfunction, in most cases, may not be an independent etiological factor, but an important subcellular manifestation of cholesterol neurotoxicity. Certainly, this mechanistic explanation does not exclude genetically inherited mitochondrial defects as an independent causative factor in LOAD.

*Neuroinflammation.* Chronic neuroinflammation plays a crucial role in AD progression [661,662,663]. It is speculated that neuroinflammation is largely caused by the following two major pathogenic changes: one is neuronal injury and death, which results from elevated cholesterol and reduced ATP synthesis in brain neurons. The other major cause is the accumulation of cholesterol in other brain cells, such as microglial cells, astrocytes and cells, which are actively involved in the disposition of cholesterol-loaded, A*β*-bound ApoE particles. An increase in cholesterol levels in these cells is expected to cause mitochondrial dysfunction, energy deficiency and cell death; cell death is one of the root causes for initiation and propagation of neuroinflammation.

**Figure 8:**
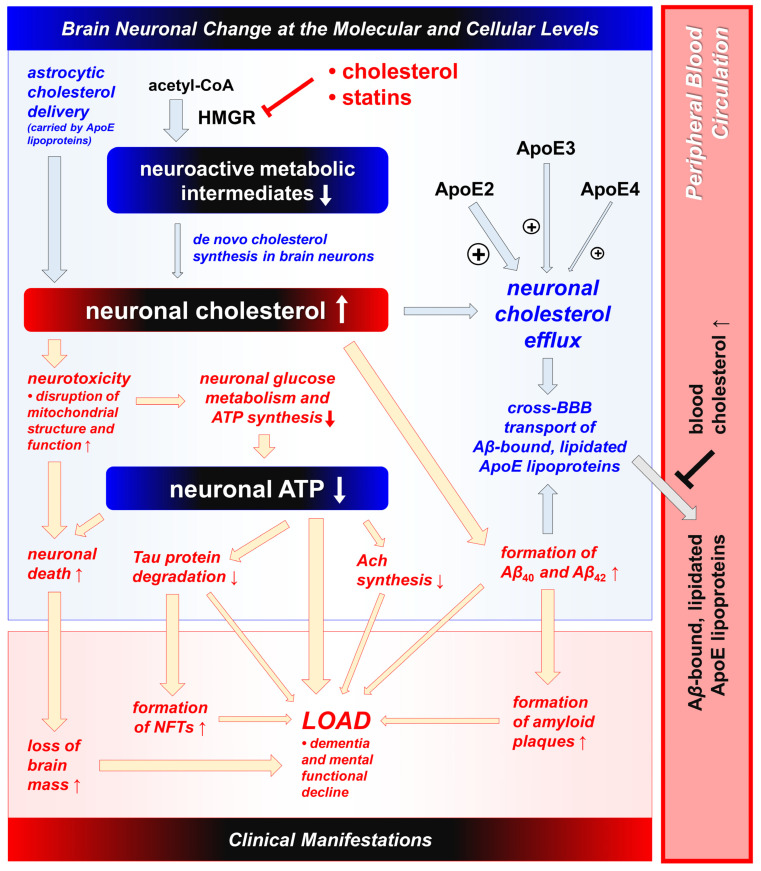
A summary diagram integrating the molecular and cellular mechanisms underlying the cholesterol-centered pathogenic hypothesis of LOAD and the corresponding clinical outcomes of the disease. The upper left panel illustrates molecular and cellular changes taking place inside brain neurons; the narrow right panel shows alterations in peripheral blood circulation (i.e., blood cholesterol levels). The lower left panel describes major clinical manifestations of LOAD and their associations with the cellular and molecular changes occurring in brain neurons. Note that italicized items represent molecular, cellular or pathogenic processes, whereas the non-italicized regular text denotes chemical entities (e.g., cholesterol, ATP, A*β* peptides, and ApoE lipoproteins).

In summary, the above mechanisms (i.e., impaired brain glucose metabolism, mitochondrial dysfunction, and neuroinflammation) are mostly direct or indirect manifestations of abnormally elevated neuronal cholesterol, although each of the mechanisms can also contribute independently to the pathogenesis of LOAD under certain conditions.

### 11.2. Limitations of the Proposed Cholesterol-Centered Hypothesis

The proposed cholesterol-centered hypothesis of AD pathogenesis also has some potential limitations. First, some results from human studies appear to be not in full agreement with the hypothesis. For instance, late-life serum and brain cholesterol levels in LOAD patients are often normal or reduced, not elevated. It is speculated that the low brain cholesterol levels in advanced LOAD may reflect disease-related metabolic change or malnutrition. Secondly, lifelong severe hypercholesterolemia (e.g., familial hypercholesterolemia) does not consistently increase LOAD risk. Here, it is of note that the intraneuronal cholesterol levels in patients with familial hypercholesterolemia may not be higher, but may actually be lower most of the time, likely resulting from decreased endocytosis (a LDLR-mediated process) of LDL particles that are loaded with cholesterol. Lastly, the proposed mechanism seems a bit oversimplified, and may have somewhat overlooked the role of non-cholesterol pathways, such as metabolic deficiencies, neuroinflammation, and selective synaptic degeneration. It is possible that these non-cholesterol pathways may interact dynamically with cholesterol-centered mechanisms and jointly contribute to disease progression in a complementary or competing manner.

## 12. Concluding Remarks

Few diagnoses in medicine are more dispiriting for patients and their families than AD. This disease, which causes insidious dissolution of one’s most human qualities, namely, reasoning, abstraction, language and memory, now affects approximately 50 million individuals worldwide [2]. Despite the fact that substantial consensus has been developed revealing that certain biochemical changes in hippocampus and association cortices occur many years before clinical symptoms, the study of AD is presently still fraught with mechanistic ignorance and therapeutic nihilism. Agreements on the temporal sequence of the molecular, biochemical and cellular events leading to dementia and which steps are most amenable to effective intervention have been difficult to achieve.

As summarized in Figure 8, a novel hypothesis is developed in this paper, which speculates that in most cases of sporadic LOAD, the abnormally elevated cholesterol in brain neurons represents a crucial causative factor that drives the pathogenic processes of LOAD. Specifically, the elevated neuronal cholesterol will disrupt mitochondrial structure and metabolic activity, resulting in ATP deficiency as well as reduced formation of neuroactive metabolic intermediates along the cholesterol synthesis pathway in brain neurons. In addition, the abnormally elevated neuronal cholesterol will cause direct neuronal damage as well as other pathogenic changes in the brain, including increased formation and aggregation of A*β* plaques and tauopathy as well as reduced formation of cholinergic vesicles. It is further hypothesized that A*β* accumulation and plaque formation in most LOAD cases only represent characteristic secondary pathological changes, and are usually not the dominant force that drives the pathogenic process of LOAD.

Genetic factors play a crucial role in the pathogenesis of sporadic LOAD. ApoE is now recognized as the most important genetic risk factor in sporadic LOAD. While the best-characterized function of the brain ApoE proteins is their delivery of astrocyte-derived cholesterol to neurons, another function of brain ApoE proteins is their ability to remove excess neuronal cholesterol. It is hypothesized that the differences in ApoE’s ability to efficiently remove excess neuronal cholesterol together with its differential ability to bind A*β*_40_ and A*β*_42_ jointly determines its pathogenic role in many LOAD cases. There are many good reasons for this suggestion. First, the markedly lower ability of ApoE4 to efflux excess neuronal cholesterol will result in elevated neuronal cholesterol, which suppresses mitochondrial metabolic activity and reduces the synthesis of ATP and neuroactive metabolic intermediates. These effects are detrimental to the normal functioning of neurons as well as their survival. Second, increase in neuronal membrane cholesterol or CE content will lead to activation of *β*-/*γ*-secretases, resulting in increased formation of A*β*_42_ and A*β*_40_ peptides and subsequently, increased deposition of amyloid plaques. Additionally, different ApoE isoforms have different ability to bind A*β*_42_ and A*β*_40_ peptides, which also affects amyloid deposition. Lastly, severe deficiency of neuronal ATP will reduce the production of cholinergic vesicles as well as the formation of tauopathy. These pathogenic effects jointly contribute to the development of LOAD. As discussed in considerable detail in this paper, the proposed pathogenic mechanism of AD offers a good explanation for the clinical observations of a higher AD risk for the *APOE4* genotype and a reduced risk for the *APOE2* genotype.

In addition, it is quite readily understood that other genetic risk factors also affecting neuronal cholesterol homeostasis may contribute, directly or indirectly, to the pathogenesis of LOAD (discussed in ref. [98]). The relationship between these genetic factors and cholesterol dyshomeostasis is really intriguing, which actually offers additional support for a pivotal pathogenic role of neuronal cholesterol dyshomeostasis in LOAD.

Notably, brain neurons are among a group of cells in the body that have the highest demand for oxygen and energy supply, and adequate supply of cellular ATP in neurons is vital for normal cognitive function and memory formation. Significant reductions in mitochondrial ATP synthesis, which will lead to neuronal energy deficit, are believed to play a crucial role in driving the pathogenic process of all forms of AD. Accordingly, it is speculated that AD usually begins with neuronal injury in brain regions that likely have the highest demand for energy supply (ATP synthesis), and then gradually spreads to other brain regions with a relatively lower demand for energy supply, and eventually to the whole brain. In line with this suggestion, it is known that AD usually begins in the entorhinal cortex and then proceeds to the hippocampus, a waystation important in memory formation. As the hippocampal neurons degenerate, short-term memory falters. Often the ability to perform routine tasks begins to deteriorate as well. It then gradually spreads to other regions, particularly the cerebral cortex which is the outer area of the brain involved in functions such as language and reasoning. In the diseased regions, the neurons degenerate, lose their connections or synapses with other neurons, and some of the neurons die as a result.

Since the introduction of the amyloid hypothesis in 1991, it has attracted enormous research interest and attention. It was postulated that A*β*_42_ can readily form aggregates which then initiate a pathogenic cascade, ultimately resulting in neuronal loss and dementia. Over the years, there were many clinical and animal studies showing that the abundance of amyloid deposition and plaque formation often does not correlate with the severity of memory deficits or neuronal toxicity. As discussed in this paper, if we put aside the notion that *Aβ* plaque formation is a driving force in AD development, and if we simply add the neuronal cholesterol as a key component in the pathogenic process, then most of the puzzle pieces appear to fit together much better, i.e., the mental functional decline is mostly caused by cholesterol-induced deficits in ATP and neuroactive metabolic intermediates in brain neurons, whereas A*β* accumulation and plaque formation often only represent a secondary event, rather than the initial driving force in LOAD pathogenesis. As discussed in this paper, even in familial EOAD cases, abnormal cholesterol buildup in brain neurons is still a key player in the pathogenic process.

It is known that increases in the content of cholesterol (particularly CEs) in neuronal membranes increase A*β* formation and deposition, and it is hypothesized that tau accumulation is mostly the result of neuronal ATP deficits. At present, it is difficult to precisely pinpoint which of these two events occurs earlier—it likely depends on the brain regions affected as they have different levels of energy demand. As elevated neuronal cholesterol can directly alter *β*-/*γ*-secretase activities, it is generally true that increased A*β* formation is usually seen very early on in most LOAD patients. Similarly, tau accumulation may also occur relatively early in selected brain regions where neurons have a particularly high demand for energy supply, and neuronal ATP deficiency resulting from elevated cholesterol will be preferentially experienced and thus will trigger the accumulation of tau. Overall, as mental functions are more closely associated with the degree of neuronal ATP deficiency, this is the main reason why mental functional decline often is better correlated with tauopathy rather than amyloid accumulation in the brain.

At present, there is still no cure for AD. The available treatments offer relatively small symptomatic benefits, but do not address the underlying pathological process or substantially slow down clinical progression. In light of the new understanding that A*β* accumulation may only represent a secondary change in most LOAD cases, *β*-/*γ*-secretase inhibitors and A*β* vaccines may have very limited benefits for improving the cognitive functions of LOAD. Similarly, tau inhibitors may also have a limited benefit, given that tau aggregation is a direct consequence of severe neuronal ATP deficiency. On the other hand, strategies aiming at reducing neuronal cholesterol levels may be of great benefit in AD treatment and prevention. For instance, the LXR agonists will activate the expression of ApoE, ABCA1 and ABCG1, resulting in enhanced neuronal cholesterol efflux. CYP46A1 inducers may also have a similar effect. It is suggested that the use of low-dose, peripherally acting statins, which aim to improve hypercholesterolemia, will be of benefit for reducing the risk of LOAD. Based on the proposed hypothesis, it is apparent that nutritional supplements that can safely booster the synthesis of neuronal ATP and/or neuroactive metabolic intermediates will be of great potential as adjuvant therapies in reducing the risk of developing LOAD.

In closing, it is of interest to note that during the initial characterization of the AD pathology by Dr. Alzheimer, he also noted the accumulation of “adipose inclusions”, likely neutral lipids, in glial cells from postmortem brain samples of patients with dementia (discussed in [664]). The presently proposed cholesterol-centered hypothesis on AD pathogenesis may finally shed a dawning light on the initial careful observations made by Dr. Alzheimer concerning the potential pathogenic role of the adipose-like inclusions in various brain cells of dementia patients.

## Figures and Tables

**Table 1:** Characteristics and major differences in LOAD vs. EOAD.

	LOAD (Late-Onset AD)		EOAD (Early-Onset AD)
**Definition**	● AD with symptom onset at ≥65 years of age		● AD with symptom onset at <65 years of age
**Prevalence**	● 90–95% of all AD cases		● 5–10% of all AD cases
**Inheritance** **pattern**	● Predominantly sporadic (non-familial); ● Rare autosomal dominant familial forms.		● ~50% familial (autosomal dominant); remaining 50% sporadic; ● Familial forms show near-complete penetrance.
**Genetic basis**	Polygenic and multifactorial: ● Strongest risk factor: *APOE ε4* allele; ● Low-effect susceptibility genes: *CLU*, *BIN1*, *PICALM*, *ABCA7*, etc.; ● Low penetrance; ● Environmental/lifestyle factors can modify risk.		● Familial EOAD: High-penetrance monogenic mutations in *APP*, *PSEN1* or *PSEN2*; ● Sporadic EOAD: Enriched for *APOE ε4* allele.
**Clinical** **phenotype**	Classic amnestic-predominant phenotype: ● Core initial symptom: Episodic memory loss; ● Early language impairment; ● Personality changes and behavioral symptoms appear late.		Predominantly non-amnestic atypical phenotypes: ● Core initial symptoms: Executive dysfunction, visuospatial impairment, poor judgment and planning; ● Common atypical variants: Posterior cortical atrophy (PCA), primary progressive aphasia (PPA), frontal variant AD; ● Higher rates of behavioral symptoms (agitation, apathy) early in disease.
**Neuropathology (shared)**	● Extracellular A*β* plaques (senile plaques) ● Intracellular tau neurofibrillary tangles (NFTs); ● Neuronal loss and synaptic dysfunction.		
**Neuropathology (distinct)**	● NFTs and cortical atrophy are predominantly in hippocampus and medial temporal lobe; ● High burden of mixed comorbid pathologies.		● Purer AD pathology with minimal age-related comorbid neurodegenerative/vascular lesions; ● Higher NFT density in frontal, parietal, and occipital cortices; ● More widespread neocortical A*β* deposition; ● Less hippocampal predominance of NFTs.
**Progression**	● Slow, insidious progression (gradual decline in cognition and function)		● Rapid progression (faster cognitive and functional deterioration)
**Disease duration**	● 5–8 years		● 3–5 years
**Risk factors and comorbidities**	● Strongly linked to age-related systemic conditions; ● Modifiable lifestyle factors have significant protective effects.		● Fewer metabolic/vascular comorbidities at onset; ● Lifestyle factors have weaker modifying effects.

## Data Availability

No new data were created or analyzed in this study. Data sharing is not applicable to this article.

## Abbreviations

The following abbreviations are used in this manuscript:

AD	Alzheimer’s disease
A*β*	Amyloid *β*
A*β*_40_	A*β* fragment containing 1–40 amino acid residues
A*β*_42_	A*β* fragment containing 1–42 amino acid residues
APP	Amyloid-*β* precursor protein
AICD	APP intracellular domain
ApoE	Apolipoprotein E
BACE1	*β*-site APP cleaving enzyme
ApoJ	Apolipoprotein J
CNS	Central nervous system
GSH	Glutathione
HMGR	HMG-CoA reductase
ATP	Adenosine triphosphate
ACAA	Acetyl-CoA acyltransferase
SOAT1	Sterol *O*-acyltransferase 1 (also called ACAT1)
ACAT1	Acyl-CoA:cholesterol acyltransferase
LCAT	Lecithin:cholesterol acyltransferase
SREBP2	Sterol-dependent transcription factor
ER	Endoplasmic reticulum
LDL	Low-density lipoprotein
LDLR	LDL receptor
VLDL	Very low-density lipoprotein
VLDLR	VLDL receptor
HDL	High-density lipoprotein
LRP1	LDL receptor-related protein 1
ApoER2	ApoE receptor 2
NPC1 or NPC2	Niemann–Pick type C1 or C2, respectively
NPC disease	Niemann–Pick type C disease
ABCA1	ATP binding cassette transporter A1
StAR protein	Steroidogenic acute regulatory protein
STARD3	StAR-related lipid transfer protein domain 3
CYP	Cytochrome P450
24*S*-OHC	24*S*-hydroxycholesterol
LXR	Liver X receptor
RXR	Retinoid X receptor
CSF	Cerebrospinal fluid
MAP	Microtubule-associated protein
NFT	Neurofibrillary tangle
ChAT	Acetyl-CoA:choline *O*-acetyltransferase
AChE	Acetylcholinesterase
BBB	Blood–brain barrier
TREM2	Triggering receptor expressed on myeloid cells 2
DAM phenotype	Disease-associated microglia phenotype
NSAIDs	Nonsteroidal anti-inflammatory drugs
BDNF	Brain-derived neurotrophic factor
IGF-1	Insulin-like growth factor
VEGF	Vascular endothelial growth factor
NOS	Nitric oxide synthase
